# Supplementary material for: Strengthening the clinical academic pathway: a systematic review of interventions to support clinical academic careers for doctors and dentists
Source: BMJ Open. 2022 Sep 8;12(9):e060281. doi: 10.1136/bmjopen-2021-060281 (PMC9462120; doi:10.1136/bmjopen-2021-060281)
Supplement: Supplementary data [file bmjopen-2021-060281supp001.pdf]

Appendix 1: Medline search strategy

MEDLINE(R) ALL

via Ovid http://ovidsp.ovid.com/
1946 to October 25, 2019
Searched on: 28th October 2019
Records retrieved search B: 13473
Records retrieved search A: 4241

1 ((doctor or doctors or physician$ or medic or medics) adj4 academi$).ti,ab. (1872)
2 ((doctor or doctors or physician$ or medic or medics) adj4 (professor$ or dean$ or program$ director$ or lecturer$ or research fellow$ or researcher$)).ti,ab. (1979)
3 ((doctor or doctors or physician$ or medic or medics) adj4 (doctora$ or predoctora$ or pre-doctora$ or postdoctora$ or post-doctora$ or postdoc or post-doc or postdocs or post-docs or PhD or PhDs)).ti,ab. (232)
4 ((doctor or doctors or physician$ or medic or medics) adj4 (universit$ or higher education or research institut$ or research centre$ or research center$)).ti,ab. (1435)
5 (medical adj (profession$ or practitioner$ or specialist$) adj4 academi$).ti,ab. (65)
6 (medical adj (profession$ or practitioner$ or specialist$) adj4 (professor$ or dean$ or program$ director$ or lecturer$ or research fellow$ or researcher$)).ti,ab. (154)
7 (medical adj (profession$ or practitioner$ or specialist$) adj4 (doctora$ or predoctora$ or pre-doctora$ or postdoctora$ or post-doctora$ or postdoc or post-doc or postdocs or post-docs or PhD or PhDs)).ti,ab. (5)
8 (medical adj (profession$ or practitioner$ or specialist$) adj4 (universit$ or higher education or research institut$ or research centre$ or research center$)).ti,ab. (59)
9 ((GP or GPs or general practioner$) adj4 academi$).ti,ab. (75)
10 ((GP or GPs or general practioner$) adj4 (professor$ or dean$ or program$ director$ or lecturer$ or research fellow$ or researcher$)).ti,ab. (96)
11 ((GP or GPs or general practioner$) adj4 (doctora$ or predoctora$ or pre-doctora$ or postdoctora$ or post-doctora$ or postdoc or post-doc or postdocs or post-docs or PhD or PhDs)).ti,ab. (4)
12 ((GP or GPs or general practioner$) adj4 (universit$ or higher education or research institut$ or research centre$ or research center$)).ti,ab. (59)
13 ((dentist or dentists) adj4 academi$).ti,ab. (63)
14 ((dentist or dentists) adj4 (professor$ or dean$ or program$ director$ or lecturer$ or research fellow$ or researcher$)).ti,ab. (73)
15 ((dentist or dentists) adj4 (doctora$ or predoctora$ or pre-doctora$ or postdoctora$ or post-doctora$ or postdoc or post-doc or postdocs or post-docs or PhD or PhDs)).ti,ab. (20)
16 ((dentist or dentists) adj4 (universit$ or higher education or research institut$ or research centre$ or research center$)).ti,ab. (72)
17 ((dental or dentistry) adj (profession$ or practitioner$ or specialist$) adj4 academi$).ti,ab. (24)
18 ((dental or dentistry) adj (profession$ or practitioner$ or specialist$) adj4 (professor$ or dean$ or program$ director$ or lecturer$ or research fellow$ or researcher$)).ti,ab. (22)
19 ((dental or dentistry) adj (profession$ or practitioner$ or specialist$) adj4 (doctora$ or predoctora$ or pre-doctora$ or postdoctora$ or post-doctora$ or postdoc or post-doc or postdocs or post-docs or PhD or PhDs)).ti,ab. (1)
20 ((dental or dentistry) adj (profession$ or practitioner$ or specialist$) adj4 (universit$ or higher education or research institut$ or research centre$ or research center$)).ti,ab. (23)
21 or/1-20 (6146)
22 ((consultant$ or registrar$ or associate specialist$ or staff grade$ or house officer$ or houseman or housemen or housestaff) adj4 academi$).ti,ab. (139)
23 ((consultant$ or registrar$ or associate specialist$ or staff grade$ or house officer$ or houseman or housemen or housestaff) adj4 (professor$ or dean$ or program$ director$ or lecturer$ or research fellow$ or researcher$)).ti,ab. (242)

24 ((consultant$ or registrar$ or associate specialist$ or staff grade$ or house officer$ or houseman or housemen or housestaff) adj4 (doctora$ or predoctora$ or pre-doctora$ or postdoctora$ or post-doctora$ or postdoc or post-doc or postdocs or post-docs or PhD or PhDs)).ti,ab. (18)
25 ((consultant$ or registrar$ or associate specialist$ or staff grade$ or house officer$ or houseman or housemen or housestaff) adj4 (universit$ or higher education or research institut$ or research centre$ or research center$)).ti,ab. (166)
26 ((medical or specialt$ or specialist$ or clinical or surgical) adj4 train$ adj4 academi$).ti,ab. (445)
27 ((medical or specialt$ or specialist$ or clinical or surgical) adj4 train$ adj4 (professor$ or dean$ or program$ director$ or lecturer$ or research fellow$ or researcher$)).ti,ab. (188)
28 ((medical or specialt$ or specialist$ or clinical or surgical) adj4 train$ adj4 (doctora$ or predoctora$ or pre-doctora$ or postdoctora$ or post-doctora$ or postdoc or post-doc or postdocs or post-docs or PhD or PhDs)).ti,ab. (116)
29 ((medical or specialt$ or specialist$ or clinical or surgical) adj4 train$ adj4 (universit$ or higher education or research institut$ or research centre$ or research center$)).ti,ab. (468)
30 ((FY1 or FY2 or SHO or JHO or FY train$ or CMT or CST) adj10 academi$).ti,ab. (4)
31 ((FY1 or FY2 or SHO or JHO or FY train$ or CMT or CST) adj10 (professor$ or dean$ or program$ director$ or lecturer$ or research fellow$ or researcher$)).ti,ab. (20)
32 ((FY1 or FY2 or SHO or JHO or FY train$ or CMT or CST) adj10 (doctora$ or predoctora$ or pre-doctora$ or postdoctora$ or post-doctora$ or postdoc or post-doc or postdocs or post-docs or PhD or PhDs)).ti,ab. (0)
33 ((FY1 or FY2 or SHO or JHO or FY train$ or CMT or CST) adj10 (universit$ or higher education or research institut$ or research centre$ or research center$)).ti,ab. (31)
34 or/22-33 (1774)
35 (facult$ adj5 (medical or medicine or dental or dentistry or clinical) adj5 (academi$ or research$ or scholar$)).ti,ab. (1313)
36 (facult$ adj5 (medical or medicine or dental or dentistry or clinical) adj5 (professor$ or dean$ or program$ director$ or lecturer$ or research fellow$ or researcher$)).ti,ab. (399)
37 (facult$ adj5 (medical or medicine or dental or dentistry or clinical) adj5 (doctora$ or predoctora$ or pre-doctora$ or postdoctora$ or post-doctora$ or postdoc or post-doc or postdocs or post-docs or PhD or PhDs)).ti,ab. (79)
38 or/35-37 (1705)
39 exp Physicians/ (133681)
40 exp Dentists/ (18486)
41 Faculty, Medical/ (12770)
42 Faculty, Dental/ (2398)
43 Academic Medical Centers/ (17857)
44 39 or 40 or 41 or 42 or 43 (180811)
45 Research Personnel/ (15675)
46 Universities/ (38946)
47 research/ or biomedical research/ or dental research/ (267864)
48 45 or 46 or 47 (313057)
49 44 and 48 (7101)
50 21 or 34 or 38 or 49 (15969)
51 (academic adj (medicine or dentistry or primary care)).ti,ab. (2568)
52 (academic adj2 (an?esthesi$ or an?estheti$ or oncolog$ or emergency medicine or radiolog$ or intensive care or intensivist$ or obstetric$ or gyn?ecolog$ or ophthalmolog$ or paediatric$ or pediatric$ or patholog$ or psychiatr$ or public health or surgery or surgeon$)).ti,ab. (4937)
53 51 or 52 (7395)
54 ((clinical or clinician$ or medical or dental or dentistry) adj academi$).ti,ab. (780)
55 ((clinical or clinician$ or medical or dental or dentistry) adj (lecturer$ or lectureship$)).ti,ab. (61)
56 ((clinical or clinician$ or medical or dental or dentistry) adj professor$).ti,ab. (177)

57 ((clinical or clinician$ or medical or dental or dentistry) adj fellow$).ti,ab. (325)
58 ((clinical or clinician$ or medical or dental or dentistry) adj research fellow$).ti,ab. (37)
59 in-practice fellow$.ti,ab. (8)
60 clinical research train$.ti,ab. (103)
61 physician$ scientist$.ti,ab. (854)
62 surgeon$ scientist$.ti,ab. (165)
63 ((clinical or clinician$) adj scientist$).ti,ab. (1193)
64 ((clinical or clinician$) adj scholar$).ti,ab. (175)
65 ((clinical or clinician$) adj researcher$).ti,ab. (2597)
66 ((clinical or clinician$) adj investigator$).ti,ab. (1649)
67 ((clinical or clinician$) adj educator$).ti,ab. (1011)
68 or/54-67 (8891)
69 50 or 53 or 68 (30102)
70 (integrated adj3 academic adj3 (train$ or career$ or path or paths or pathway$ or program$)).ti,ab. (33)
71 (IAT adj2 (career$ or path$ or program$)).ti,ab. (10)
72 Clinical Research Training Fellowship$.ti,ab. (10)
73 Academic Foundation Program$.ti,ab. (17)
74 (academi$ adj3 (clinical or clinician$ or medical or medicine or dental or dentistry) adj3 (career$ or path or paths or pathway$)).ti,ab. (383)
75 (research$ adj3 (clinical or clinician$ or medical or medicine or dental or dentistry) adj3 (career$ or path or paths or pathway$)).ti,ab. (330)
76 or/70-75 (751)
77 Career Choice/ (22485)
78 career mobility/ (11228)
79 Staff Development/ (9142)
80 (career$ or pathway$ or pipeline$).ti,ab. (1105747)
81 ((occupation$ or profession$ or job$ or staff or employee$ or personnel) adj3 (choice$ or choos$ or select$ or decid$ or decision$)).ti,ab. (7714)
82 ((occupation$ or profession$ or job$ or staff or employee$ or personnel) adj3 (mobility or ladder$ or route$ or trajector$ or structure$)).ti,ab. (2939)
83 ((occupation$ or profession$ or job$ or staff or employee$ or personnel) adj3 (progress$ or promot$ or develop$ or advanc$)).ti,ab. (26577)
84 or/77-83 (1166195)
85 69 and 84 (4314)
86 Personnel Selection/ (12625)
87 (recruit$ or hire$ or hiring).ti,ab. (355306)
88 86 or 87 (363489)
89 69 and 88 (1460)
90 Personnel Turnover/ (5027)
91 (retain$ or retention).ti,ab. (350726)
92 (resign$ or terminat$ or disenroll$ or withdraw$ or attrition).ti,ab. (247203)
93 90 or 91 or 92 (595449)
94 69 and 93 (887)
95 85 or 89 or 94 (5724)
96 76 or 95 (6051)
97 exp animals/ not humans/ (4637358)
98 96 not 97 (6040)
99 limit 98 to english language (5741)
100 limit 99 to yr="2004 -Current" (4471)
101 (editorial or letter).pt. (1554107)
102 100 not 101 (4241) **[Records downloaded for search A – clinical academics AND career]**
103 69 or 76 (30413)
104 103 not 97 (30248)

105 limit 104 to english language (27498)
106 limit 105 to yr="2004 -Current" (18930)
107 106 not 101 (17714)
108 107 not 102 (13473) **[Records downloaded for search B – clinical academics with search A results removed]**
