# Supplementary material for: Strengthening the clinical academic pathway: a systematic review of interventions to support clinical academic careers for doctors and dentists
Source: BMJ Open. 2022 Sep 8;12(9):e060281. doi: 10.1136/bmjopen-2021-060281 (PMC9462120; doi:10.1136/bmjopen-2021-060281)
Supplement: Supplementary data [file bmjopen-2021-060281supp002.pdf]

Appendix 2: Table of data for studies included in synthesis

First author (Year of publication; country)	Years of data collection	Study design	Grade/type of clinical academics included	Number of participants (Male: Female)	Ethnicity	Intervention components	Target population of intervention	Aimed at particular group	Reach
Studies included within quantitative analysis									
Brandt (2018; USA)[19]	NR- NR	Cohort study. Concurrent & historical control	Orthopaedic surgery residents, academic level NR	329 (NR)	NR	Mentorship programme - junior mentee/senior mentor, Protected research time	Trainees	No	Single centre
Chang (2016; USA)[21]	1988-2009	Cohort study. Concurrent control	Clinically mixed group, post-training, range of academic levels	65099, of which 3268 in intervention group	NR	Leadership training. Faculty career development program, not described in detail for individual programs. EWIM & MWIM programs focus on academic skills development; ELAM is leadership training	Junior Faculty, Managers/ leaders/ senior faculty	Gender	National
Daley (2006; USA)[22]	NR- 2005	Cohort study. Concurrent & historical control	Mixed group of faculty (medical, surgical, psychiatry, GP), mixed academic levels	112 (48:64)	15 URM	Mentorship programme - junior mentee/ senior mentor. Teaching sessions/ lectures, leadership training, faculty career development programme, networking/ collaboration/ social activities, academic performance counselling	Junior Faculty	Ethnicity, URM	Single centre
Dannels (2008; USA)[23]	2002-2006	Cohort study. Concurrent control	Post-training, post-doctoral. Speciality NR	250 (0:250)	NR	Leadership training	Managers/ leaders/ senior faculty	Gender	Single centre
Ehlers (2018; USA)[24]	2018-2018	Cohort study. Concurrent control	Pre-doctoral fellows in cardiovascular diseases, gastroenterology and hepatology/ oncology	140 (76:64)	NR	Mentorship programme - junior mentee/ senior mentor. Teaching sessions/ lectures, protected research time. Teaching is focused on research training. E.g. research methods, protocol development, grant writing and manuscript writing, statistics and epidemiology. Also complete a mentored research project	Trainees, Junior Faculty	No	Single centre
Emans (2008; USA)[25]	2006- NR	Cohort study. Historical control	Academic paediatric faculty. Academic level NR	238 (54%:46%)	NR	Mentorship programme - junior mentee/ senior mentor. Teaching sessions/ lectures, leadership training, networking/ collaboration/ social activities, 'Community	Junior Faculty	Gender, Ethnicity	Single centre

First author (Year of publication; country)	Years of data collection	Study design	Grade/type of clinical academics included	Number of participants (Male: Female)	Ethnicity	Intervention components	Target population of intervention	Aimed at particular group	Reach
						of mentors'. Work-life balance, cultural competency and diversity initiatives			
Goldenberg (2012; USA)[26]	NR- NR	Case control. Concurrent control	Trainees in academic haematology/ oncology. Academic level NR	20 (10 females)	NR	Mentorship programme - junior mentee/ senior mentor	Trainees	No	National
Grisso (2017; USA)[27]	2010- NR	Multifaceted cluster randomised intervention trial	Assistant professors, speciality NR	132 (0:132)	7.6% African American, 60.3% White, 27.5% Asian, 4.6% Hispanic/ other	Mentorship programme - junior mentee/ senior mentor. Mentorship programme - peers, grant writing programme, teaching sessions/ lectures, networking/ collaboration/ social activities, manuscript writing programme, also included a "Total Leadership Programme" which address work-life integration/ balance	Junior Faculty	Gender	Single centre
Harrison (2020; USA)[29]	2007-2017	Cohort study. Historical control	General surgery residents, academic level NR	203 (NR)	NR	Mentorship programme - junior mentee/ senior mentor. Teaching sessions/ lectures, annual milestones, research meetings and feedback on completing research projects	Trainees	No	Single centre
Joshua Smith (2014; USA)[30]	NR-NR	Cohort study. Concurrent control	General surgery trainees. Academic level NR	68 (NR)	NR	Research training; Protected research time	Trainees	No	Single centre
Khot (2011; USA)[31]	2007-2007	Cohort study. Concurrent control	Post-training, post-doctoral. Speciality not reported	1577 NIH associates (4 female) who entered academic medicine; 27821 non-associates	NR	Teaching sessions/ lectures, protected research time. Little detail reported	Trainees	No	National
Klimas (2017; Canada)[45]	2014-2014	Cohort study. Concurrent control	Trainees in internal medicine, family medicine, public health,	8 (3:5)	NR	Mentorship programme - junior mentee/ senior mentor. Teaching sessions/ lectures	Trainees	No	National

First author (Year of publication; country)	Years of data collection	Study design	Grade/type of clinical academics included	Number of participants (Male: Female)	Ethnicity	Intervention components	Target population of intervention	Aimed at particular group	Reach
			psychiatry, mix of academic levels						
Kohlwes (2006; USA)[32]	2001-2004	Cohort study. Concurrent control	Trainees in internal medicine. Academic level NR	32 (NR)	NR	Mentorship programme - junior mentee/senior mentor. Teaching sessions/ lectures, protected research time, small group journal clubs; work in progress sessions; conduct a clinical research project	Trainees	No	Single Centre
Kohlwes 2016; USA)[33]	NR- NR	Cohort study. Concurrent control	Post-training in internal medicine. Academic level NR	169 (88:81)	88 white, 1 black, 31 Asian Indian, 28 Asian Other, 12 Hispanic, 6 Other	Mentorship programme - junior mentee/ senior mentor. Teaching sessions/ lectures, protected research time, participants conduct research projects. Salary support provided for PRIME director and associate director	Trainees	No	Single Centre
Libby (2016; USA)[34]	2000-2011	Cohort study. Concurrent control	Faculty from many disciplines of medicine/surgery as well as disciplines outside medicine. Post-doctoral	25 CFSP scholars; 125 comparison faculty (NR)	NR	Mentorship programme - junior mentee/ senior mentor. Mentorship programme - peers, teaching sessions/ lectures, protected research time	Junior Faculty	No	Single centre
Löwe (2008; Western Europe)[48]	2005-2006	Cohort study. Concurrent control	Trainees from Internal Medicine, psychotherapy, psychosomatics, psychiatry, psychology. Academic level NR	44 (17:27)	NR	Mentorship programme - junior mentee/ senior mentor. Teaching sessions/ lectures, teaching sessions held as part of a 'clinical research methods' course. Participants also conducted an individual research project	Trainees	No	Single centre
Mandel (2018; USA)[35]	NR- NR	Cohort study. Historical control	Post-training plastic surgeons, academic level NR	28 (24:4)	NR	Teaching sessions/lectures, protected research time. 1-2 year research fellowship, senior mentorship	Trainees	No	Single centre
Merani (2014; Canada)[46]	1988-2012	Cohort study. Concurrent control	General surgery residents, mixed pre-doctoral and doctoral	323 (NR)	NR	Research training	Trainees	No	National

First author (Year of publication; country)	Years of data collection	Study design	Grade/type of clinical academics included	Number of participants (Male: Female)	Ethnicity	Intervention components	Target population of intervention	Aimed at particular group	Reach
Mills (2011; USA)[36]	Post 2007-NR	Cohort study. Concurrent control	Paediatric residents and fellows, academic level NR	526 (197:297)	NR	Departmental research day at which paediatrics trainees present submitted research and other scholarly work	Trainees	No	Single centre
Nasab (2019; USA)[37]	2018-2018	Cohort study. Concurrent control	Obstetricians & Gynecologists, trainees and post-training, range of academic levels	109 attended, 94 pre-course survey, 74 post course survey (NR)	63% white, 9% black, 5% Asian, 3% other, 20% did not disclose	Mentorship programme - junior mentee/ senior mentor. Teaching sessions/lectures, leadership training, networking/ collaboration/ social activities. Particular sessions included mock interviews, CV reviews, and grant writing support. 60-minute interactive sessions. Participants also given the opportunity to submit their grant proposals	Trainees, Junior Faculty	No	Single Centre
Ockene (2017; USA)[38]	NR- NR	Cohort study. Concurrent control	Post-doctoral, post-training, primary care physicians	32 (11:21)	NR	Mentorship programme - junior mentee/ senior mentor. Mentorship programme – peers. Teaching sessions/ lectures, networking/ collaboration/ social activities	Junior Faculty	No	Single centre
Patel (2018; USA)[39]	2011-2016	Cohort study. Historical control	General surgery residents and faculty mentors. Mentors post-doctoral	67 (NR)	NR	Mentorship programme - junior mentee/ senior mentor. Teaching sessions/ lectures, protected research time, completion of a defined clinical research project. Other didactic activities included: active participation in the department's weekly clinical research meeting; attend lab meetings with faculty research mentor. Webinars on research and grant writing	Trainees	No	Single centre
Ries (2009; USA)[40]	1988-2006	Cohort study. Concurrent control	Assistant professors in school of medicine. Speciality NR	839 (520-319)	52 URM	Mentorship programme - junior mentee/ senior mentor. Networking/ collaboration/ social activities, professional development workshops, performance counselling sessions	Junior Faculty	No	Single centre
Ries (2012; USA)[41]	Post 2005-2006	Case control. Concurrent control	Assistant professors from mixed specialities	315 (152:163)	26 URM	Mentorship programme - junior mentee/ senior mentor. Networking/ collaboration/ social activities, faculty career development programme, Performance counselling sessions	Junior Faculty	No	Single centre

First author (Year of publication; country)	Years of data collection	Study design	Grade/type of clinical academics included	Number of participants (Male: Female)	Ethnicity	Intervention components	Target population of intervention	Aimed at particular group	Reach
Sheridan (2010; USA)[42]	2000-2008	Cohort study. Concurrent control	Post-training. Speciality NR. Academic level NR	163 (NR)	NR	Recruitment training (e.g. to reduce bias in interviews)	Junior Faculty, Managers/ leaders/ senior faculty	Gender, Ethnicity, "Individuals from any group that has been historically under-represented on the faculties of academic health centres"	Single centre
Sweeny (2019; Australia/New Zealand)[47]	2015-2018	Cohort study. Historical control	Emergency Medicine, grade and academic level NR	33 Emergency departments (NR)	NR	Networking/ collaboration/ social activities, research support	Clinical researchers in hospital departments	No	National
Valantine (2014; USA)[43]	2011-2011	Cohort study. Concurrent control	Post-training, post-doctoral, speciality NR	Varying number of participants over several time periods	NR	Mentorship programme - junior mentee/ senior mentor. Funding award, teaching sessions/ lectures, leadership training, protected research time, networking/ collaboration/ social activities, McCormick faculty awards. Faculty Fellows leadership program - individualised career development planning, skill building workshops, and the Women's faculty networking program. Intervention involved a series of programmes	Junior Faculty, Managers/ leaders/ senior faculty	Gender	Single centre
Winn (2018; USA)[44]	2013-2016	Cohort study, case control. Historical control	Internal medicine and paediatrics trainees, academic level NR	48 (NR)	NR	Mentorship programme - junior mentee/senior mentor. Mentorship programme – peers. Teaching sessions/lectures, protected research time, "scholarly homes"	Trainees	No	Single centre
Studies included within qualitative analysis									

First author (Year of publication; country)	Years of data collection	Study design	Grade/type of clinical academics included	Number of participants (Male: Female)	Ethnicity	Intervention components	Target population of intervention	Aimed at particular group	Reach
Archibald (2017; Canada)[58]	2013-2013	Qualitative	Physicians in family medicine, academic level NR	14 (NR)	NR	Mentorship programme - junior mentee/ senior mentor. Funding award, protected research time. Assistants and coordinators provide resources and research support, networking/ collaboration/ social activities	All faculty staff	No	Single centre
Caffrey (2016; UK)[63]	2015-2015	Qualitative	Medical school staff, grade NR, mixed academic levels	31 (NR)	NR	Athena SWAN programme	Trainees, Junior Faculty, Managers/ leaders/ senior faculty, Relevant to whole of department	Gender	National
Comeau (2017; USA)[49]	2007-2014	Mixed methods. Quantitative work had no control	Trainees and post-training, mix of specialities, mix of academic levels	46 (16:30)	22 White, 24 Minority	Mentorship programme - junior mentee/ senior mentor. Funding award, teaching sessions/ lectures, protected research time	Junior Faculty	No	National
Darbyshire (2019; UK)[64]	2014-2014	Mixed methods. Quantitative work had no control	AFP Trainees (survey) and post-training (interviews), mix of specialities, pre-doctoral	34 survey; 7 interviews (NR)	NR	Research training programme; protected research time	Trainees	No	National
*DeCastro (2013a; USA)[50]	2010-2011	Qualitative	Post-training, mix of specialities, mixed academic levels	128 (52:76)	99 White, 7 Black, 3 Hispanic, 18 Asian	Mentorship programme - junior mentee/senior mentor. Protected research time, funding award	Junior Faculty	No	National
*DeCastro (2013b; USA)[51]	2010-2011	Qualitative	Post-training, mix of specialities, mixed academic levels	128 (52:76)	99 White, 7 Black, 3 Hispanic, 18 Asian	Mentorship programme - junior mentee/senior mentor. Protected research time, funding award	Junior Faculty	No	National

First author (Year of publication; country)	Years of data collection	Study design	Grade/type of clinical academics included	Number of participants (Male: Female)	Ethnicity	Intervention components	Target population of intervention	Aimed at particular group	Reach
Hayward (2011; Canada)[59]	2008-2008	Mixed methods. Quantitative work had no control	Trainees and post-training, mix of specialities, mix of academic levels	211 (survey); 13 (qualitative) (NR)	NR	Teaching sessions/ lectures, protected research time, mentorship programme - junior mentees/ senior mentors	Trainees	No	National
Helitzer (2016; USA)[52]	2011- NR	Qualitative (Part of a larger mixed methods study but only qualitative findings reported in this paper)	Post-training, mix of specialities, mixed academic levels	45 (0:45)	NR	Not described in detail for the three programmes separately. EWIM & MWIM held over three days and involved skills and confidence building. ELAM programme was leadership training. All three are faculty career development programmes	Junior Faculty, Managers/ leaders/ senior faculty	Gender	National
Iversen (2014; UK)[65]	2010-2010	Mixed methods. Quantitative work had no control	Post-training, mix of specialities, academic level unclear	147 mentees (67% male) & 77 mentors (81% male)	NR	Mentorship programme - junior mentee/ senior mentor	Junior Faculty	No	National
Jones (2019; USA)[53]	2018-2018	Qualitative study	Early-career or junior physician-scientists, range of specialities, academic level NR	28 (5:23)	11 Non-Hispanic white; 7 Asian; 7 Other; 3 NR	Funding award. Some institutions have also provided access to other forms of support, such as leadership development training, training in academic and career development skills, and/or networking.	Junior Faculty	No, but 85% of awardees are women and tailored more towards those with extra-professional caregiving demands	National
*Klimas (2017a; Canada)[60]	2015-2015	Qualitative	Fellowship in addiction medicine. Trainees from Psychiatry,	26 (12:14)	NR	Mentorship programme - junior mentee/ senior mentor. Funding award, teaching sessions/ lectures, specialty training	Trainees	No	Single centre

**First author (Year of publication; country)**	**Years of data collection**	**Study design**	**Grade/type of clinical academics included**	**Number of participants (Male: Female)**	**Ethnicity**	**Intervention components**	**Target population of intervention**	**Aimed at particular group**	**Reach**
			Internal Medicine, Family Medicine, Nursing & Social Work. Academic level NR						
*Klimas (2017b; Canada)[61]	2015-2015	Qualitative	Fellowship in addiction medicine. Trainees from Psychiatry, Internal Medicine, Family Medicine, Nursing & Social Work. Academic level NR	26 (12:14)	NR	Mentorship programme - junior mentee/ senior mentor. Funding award, teaching sessions/ lectures, specialty training	Trainees	No	Single centre
Kraemer (2018; USA)[54]	2014-2016	Mixed methods. Quantitative work had no control	Residents and fellows in medical specialities. Academic level NR	69 (41 residents, 28 fellows)	NR	Teaching sessions/ lectures. Faculty career development programme	Trainees	No	Single centre
Lin (2019; USA)[55]	Post 2009- NR	Qualitative	Post-training, post-doctoral, emergency medicine	17 (0:17)	12 Caucasian; 4 Asian American; 1 African American.	Mentorship programme - junior mentee/ senior mentor. Networking/ collaboration/ social activities, skill enhancement, e.g. negotiating skills	Junior Faculty, Managers/ leaders/ senior faculty	Gender	National
Moss (2008; Canada)[62]	2005-2005	Qualitative	Lecturers and assistant professors in Psychiatry	10 (6:4)	NR	Mentorship programme - peers	Junior Faculty	No	Single centre
Reader (2015; USA)[56]	2009-2009	Mixed methods. Quantitative work had no control	Post-training, speciality NR, mix of academic levels	10 (2:8)	NR	Mentorship programme - junior mentee/ senior mentor. Mentorship programme – peer. Teaching sessions/ lectures, protected research time. Faculty career development programme	Junior Faculty, Managers/ leaders/ senior faculty	Designed for “busy clinician educators”	Single centre

First author (Year of publication; country)	Years of data collection	Study design	Grade/type of clinical academics included	Number of participants (Male: Female)	Ethnicity	Intervention components	Target population of intervention	Aimed at particular group	Reach
Stubbe (2008; USA)[57]	NR- NR	Qualitative	Academic Child and Adolescent Psychiatry Residents. Academic level NR	5 (NR)	NR	Mentorship programme - junior mentee/ senior mentor, Teaching sessions/ lectures, Protected research time	Trainees	No	Single centre
Studies included within both quantitative and qualitative analysis									
Campion (2016; USA)[20]	2014-2014	Mixed methods. Quantitative work had concurrent control	Post-doctoral, clinical faculty and school of public health faculty	16 in intervention group (>60% women) 25 people in reference group	30% URM	Mentorship programme – peers. Teaching sessions/ lectures, protected research time, networking/ collaboration/ social activities	Junior faculty	No	Single centre
Guevara (2018; USA)[28]	NR- NR	Mixed methods. Quantitative work had concurrent control	Medical residents, junior staff and assistant profs. Academic level unclear	124 (65:59)	91 African American, 29 Hispanic Latino, 4 Native American	Funding award	Trainees, Junior Faculty	Ethnicity, URM	National

* Both papers by DeCastro et al.[50 51] and Klimas et al.[60 61] were based on data collected from a single sample of respondents.

Abbreviations: AFP – Academic Foundation Programme; CFSP – Clinical Faculty Scholars Program; CV – Curriculum Vitae; ELAM – Executive Leadership in Academic Medicine programme; EWIM – Early-career Women In Medicine program; GP – General Practitioner; MWIM – Mid-career Women In Medicine program; NR – Not reported; PRIME – Primary Medical Education program; RCT – Randomised control trial; UK – United Kingdom; URM – Underrepresented minority (study defined); USA – United States of America

BMJ Publishing Group Limited (BMJ) disclaims all liability and responsibility arising from any reliance placed on this supplemental material which has been supplied by the author(s)
