# Supplementary material for: Strengthening the clinical academic pathway: a systematic review of interventions to support clinical academic careers for doctors and dentists
Source: BMJ Open. 2022 Sep 8;12(9):e060281. doi: 10.1136/bmjopen-2021-060281 (PMC9462120; doi:10.1136/bmjopen-2021-060281)
Supplement: Supplementary data [file bmjopen-2021-060281supp003.pdf]

Appendix 3: Quality assessment details

Randomised control trial

The single eligible randomised control trial had a low risk of bias for random sequence generation, blinding of outcome assessment, and incomplete outcome data items.[27] Participant blinding showed a high risk of bias as participants knew which groups they were assigned to. A risk of recruitment bias was also evident due to the cluster-randomised design. Allocation concealment and selective outcome reporting were unclear.[27]

Case-control studies

One of the two identified case-control study was rated medium to high quality for all items.[41] The other case-control study was of lower overall quality as it demonstrated a high risk of selection bias for intervention participant selection, and no methodological or analytical adjustments were made for comparing intervention and control groups.[26]

Cohort studies

The majority of the 25 included cohort studies had representative intervention populations,[19 21 22 24 25 29-35 37-40 43 44 46-48] appropriate control groups,[19 21-25 29 30 32-40 42 44-47] high quality outcome assessment methods,[19 21 24 29-31 34 36-38 40 45-47] and long follow-up periods.[19 21-25 29-32 34-36 38-40 42-44 46 47] However, comparability was mostly deemed poor as groups were often unmatched.[19 22 25 29 31 32 35 37-39 42 45-47] Only one study demonstrated high quality on all items,[24] whilst two studies scored positively on eight out of nine items, but failed to provide cohort follow-up information.[21 40] There were additional identified risks in group selection as several studies incorporated pre-selected or highly motivated populations for intervention groups (for example, Dannels et al.[23]).

Qualitative methodology studies

The majority of qualitative studies were of high quality for demonstrating congruity between the research methodology used and the: i) research question,[49-56 58-62 64] ii) data collection methods,[49-56 58-61 64 65] iii) data analysis and presentation,[49-56 58 60 61 64] and iv) interpretation of results.[49-56 58-62 64 65] However, inadequate reporting of important information was frequent with studies failing to provide statements relating to the relationship between the researchers and research itself.[49 52 54-61 64 65] Only one study demonstrated high quality on all items of the quality assessment tool.[53]

Realist evaluation

The realist evaluation study was rated medium to high quality on the majority of the criteria.[63] Only three aspects of the evaluation were deemed inadequate, which all related to incomplete methodological reporting, especially surrounding data analysis.[63]

Mixed methods studies

The two controlled mixed methods studies were deemed high quality on the majority of criteria.[20 28] Notably, Campion et al.,[20] demonstrated a lack of reporting for complete outcome data and failed to provide high quality methodological approaches for both the qualitative and quantitative components of the study. Guevara et al.,[28] failed to provide a rationale for using a mixed methods design. Finally, it was unclear whether the intervention was conducted as intended in both studies.[20 28]
