# Supplementary material for: Strengthening the clinical academic pathway: a systematic review of interventions to support clinical academic careers for doctors and dentists
Source: BMJ Open. 2022 Sep 8;12(9):e060281. doi: 10.1136/bmjopen-2021-060281 (PMC9462120; doi:10.1136/bmjopen-2021-060281)
Supplement: Supplementary data [file bmjopen-2021-060281supp004.pdf]

Appendix 4: Table of outcome data for studies within quantitative analysis

First author (Year of publication)	Intervention group*	Control group*	Outcome	Sample size: intervention group	Sample size: control group	Estimate (95% CI/ p value/ SD)	Estimate adjusted for
**Aspiration**							
Dannels (2008)[23]	Programme participants	Two control groups: Applied but not accepted non-programme participants & general mid-career faculty women (also non-participants)	Aspiration to higher leadership position within an academic health centre (academic medicine)	55	27 & 178	76.4% vs 63.0% vs 49.4% (chi-squared= 12.903, p=0.002)	Unadjusted but faculty control group matched on academic rank, department chair status, race/ethnicity, discipline, degree type, basic sciences vs clinical department, age, medical school ownership, and awards ranking.
**Career Satisfaction**							
Campion (2016)[20]	Intervention participants	Reference group (non-participants)	Career satisfaction post-intervention	10	12	N=9 satisfied or very satisfied vs N=12 satisfied or very satisfied (NR)	Unadjusted
Grisso (2017)[27]	Intervention group	Control group (no intervention)	Work self-efficacy change scores	62	70	0.18 vs 0.24 (p=0.642)	Statistical tests were adjusted to account for correlation induced by the clustered design using generalized estimating equations. Both within-person factors (age, years in rank, race) and unit-level factors (intervention assignment) were modelled simultaneously
	Intervention group	Control group (no intervention)	Work-family conflict change (TWIF scores)	62	70	-0.13 vs -0.05 (p=0.541)	
	Intervention group	Control group (no intervention)	Work-family conflict change (SWIF scores)	62	70	-0.20 vs -0.23 (p=0.879)	
	Intervention group	Control group (no intervention)	CCWAS culture score change	Unclear; 109 in total	Unclear; 109 in total	0.03 vs 0.13 (p=0.274)	
Winn (2018)[44]	Academy participants	Pre-academy participants	Percentage of people who felt that the BCRP have been quite or extremely supportive in helping people to make career decisions	24	22	63% vs 45% (p=0.37)	Unadjusted
	Academy participants	Pre-academy participants	Percentage of people who agree or strongly	25	22	76% vs 63% (p=0.54)	

First author (Year of publication)	Intervention group*	Control group*	Outcome	Sample size: intervention group	Sample size: control group	Estimate (95% CI/ p value/ SD)	Estimate adjusted for
			agree that the BCRP guides and supports residents in pursuing and presenting scholarly projects				
**Skills & Knowledge**							
Campion (2016)[20]	Intervention participants	Reference group (non-participants)	Knowledge, Skills, and Attitudes Survey	9	11	No overall estimate. For 13 out of 32 items on the scale, participants showed greater gains than the reference group (NR)	Unadjusted
	Intervention participants	Reference group (non-participants)	Change in Sense of Community Index score (SCI-2 total score)	10	12	Mean change 10.6 vs 3.83 (p=0.2, SD=13.7 vs 9.40)	
Kohlwes (2006)[32]	Programme residents (PRIME)	Non-PRIME programme residents	Clinical competence score (average on a 9 point scale)	32	2294	8.23 vs 8.09 (p<0.001)	Unadjusted
Löwe (2008)[48]	Intervention residents	Control residents (from two different locations)	Mean change in total methodological research knowledge score from baseline	15	22	8.4 vs 1.2 (effect size =2.5, p<0.001, SD=2.9 vs 2.8)	Unadjusted
	Intervention residents	Control residents (from two different locations)	Mean change in total self-assessment research competence score from baseline	15	22	0.4 vs -0.4 (effect size=1.1, p=0.01, SD=0.9 vs 0.7)	

First author (Year of publication)	Intervention group*	Control group*	Outcome	Sample size: intervention group	Sample size: control group	Estimate (95% CI/ p value/ SD)	Estimate adjusted for
Winn (2018)[44]	Academy participants	Pre-academy participants	Percentage of people who agree or strongly agree that they have learned how to present a poster through BCRP activities	24	22	50% vs 29% (p=0.07)	Unadjusted
	Academy participants	Pre-academy participants	Percentage of people who agree or strongly agree that they have learned how to give a presentation/talk through BCRP activities	24	22	75% vs 36% (p=0.02)	
	Academy participants	Pre-academy participants	Percentage of people who agree or strongly agree with feeling prepared for non-clinical (scholarly) work after residency	24	22	63% vs 45% (p=0.37)	
**Funding**							
Goldenberg (2012)[26]	Programme trainees	Non-programme trainees	Annual grant dollars received (excluding fellowship award funding) at 3-4 years post-fellowship	11	9	Median $80,000 vs $23,000 (p=0.2)	Unadjusted
	Programme trainees	Non-programme trainees	Percentage of individuals who obtained K award within 5 years of beginning fellowship	9	8	33% vs 0% (p=0.21)	Unadjusted
Grisso (2017)[27]	Intervention group	Control group (no intervention)	Percentage of individuals with improved grants	62	70	Rate Ratio 0.75 (95% CI: 0.54-1.03, p=0.08)	Statistical tests were adjusted to account for correlation induced by the clustered design using generalized estimating equations.

First author (Year of publication)	Intervention group*	Control group*	Outcome	Sample size: intervention group	Sample size: control group	Estimate (95% CI/ p value/ SD)	Estimate adjusted for
							Both within-person factors (age, years in rank, race) and unit-level factors (intervention assignment) were modelled simultaneously
Guevara (2018)[28]	Interview applications scholars	Interview applications non-scholars	Total number of grants (mean)	76	48	2.0 vs 0.7 (p<0.001, SD=1.5 vs 1.0)	Adjusted for age, gender, race/ethnicity, application year, change of institutions, and rank at time of application
	Interview applications scholars	Interview applications non-scholars	Total grant dollars in thousands (mean)	76	48	$1,463 vs $567 (p=0.02, SD=$2,390 vs $1,507)	
Libby (2016)[34]	Programme participants (assistant professors)	Matched controls (non-programme participants, assistant professors)	Number of grant awards - change in mean before and after intervention	25	125	0.20 to 1.15 vs 0.30 to 0.49 (p<0.01)	Unadjusted but controls matched for key variables such as time in rank and dollars awarded
	Programme participants (assistant professors)	Matched controls (non-programme participants, assistant professors)	Amount of funding associated with grant awards - change in mean before and after intervention	25	125	$21,580 to $105,008 vs $26,742 to $53,716 (p<0.01)	
	Programme participants (assistant professors)	Matched controls (non-programme participants, assistant professors)	Percentage grant success rate (no. awards/no. proposals) - change before and after intervention	25	125	46.9% to 52.9% vs 42.5% to 51.7% (p=0.78)	
Löwe (2008)[48]	Intervention residents	Control residents (from two different locations)	Percentage of people who had grant applications accepted for funding	15	22	33% vs 0% (p=0.007)	Unadjusted
Sweeny (2019)[47]	After programme implementation participants	Before programme implementation participants	Number of grants awarded to new investigators	NR	NR	30 vs 20 (NR)	Unadjusted

First author (Year of publication)	Intervention group*	Control group*	Outcome	Sample size: intervention group	Sample size: control group	Estimate (95% CI/ p value/ SD)	Estimate adjusted for
**Research Participation**							
Brandt (2018)[19]	Research residents	Clinical residents	Percentage of participants actively engaged in research after intervention completion	43	284	42% vs 29% (p=0.04)	Unadjusted
Goldenberg (2012)[26]	Programme trainees	Non-programme trainees	Percentage of time spent in research post-fellowship	11	9	Median 80% vs 55% (p=0.01)	Unadjusted
Grisso (2017)[27]	Intervention group	Control group (no intervention)	Change in the number of total hours worked per week	62	70	-3.82 vs -1.39 (p=0.006)	Statistical tests were adjusted to account for correlation induced by the clustered design using generalized estimating equations. Both within-person factors (age, years in rank, race) and unit-level factors (intervention assignment) were modelled simultaneously
Harrison (2020)[29]	Participants after intervention implementation	Participants before intervention implementation	Total number of presentations (mean) - *just residents*	Unclear	Unclear	24.6 vs 3.0 (p=0.002, SD=10.24 vs 1.58)	Unadjusted
	Participants after intervention implementation	Participants before intervention implementation	Grant submissions (mean) - *just residents*	Unclear	Unclear	0.8 vs 0.0 (NR)	
	Participants after intervention implementation	Participants before intervention implementation	Total number of presentations (mean) - *just faculty*	Unclear	Unclear	40.0 vs 8.2 (p=0.025, SD=20.51 vs 1.30)	Unadjusted
Kohlwes (2016)[33]	Programme residents (PRIME)	Non-PRIME programme residents	Percentage of alumni who identified their primary role as a clinician investigator	71	98	35.2% vs 28.6% (chi-squared=0.843, p=0.358)	Unadjusted
	Programme residents (PRIME)	Non-PRIME programme residents	Mean percentage of professional time devoted	71	98	26.2% vs 20.6% (p=0.255)	

**First author (Year of publication)**	**Intervention group***	**Control group***	**Outcome**	**Sample size: intervention group**	**Sample size: control group**	**Estimate (95% CI/ p value/ SD)**	**Estimate adjusted for**
			to clinical or translational research				
Löwe (2008)[48]	Intervention residents	Control residents (from two different locations)	Percentage of people currently writing a journal article during 1 year program	15	22	86.7% vs 36.4% (p=0.003)	Unadjusted
	Intervention residents	Control residents (from two different locations)	Percentage of people currently writing a book article during 1 year program	15	22	6.7% vs 13.6% (p=0.63)	
	Intervention residents	Control residents (from two different locations)	Percentage of people who did presentations at scientific meetings during the last year	15	22	80% vs 40.9% (p=0.04)	
	Intervention residents	Control residents (from two different locations)	Percentage of people who submitted one or more grant proposal with first authorship in the last year	15	22	6.7% vs 0% (p=0.41)	
	Intervention residents	Control residents (from two different locations)	Percentage of people who submitted one or more grant proposal with co-authorship in the last year	15	22	46.7% vs 4.6% (p=0.004)	
**Mandel (2018)[35]	Graduates after residency programme transition	Graduates before residency programme transition	Percentage of graduates whose research activities were dedicated to clinical research during residency	19	9	84% vs 33% (p=0.013)	Unadjusted
	Graduates after residency programme transition	Graduates before residency programme transition	Percentage of graduates whose research activities were dedicated to full time basic	19	9	33% vs 22% (p=0.68)	

**First author (Year of publication)**	**Intervention group***	**Control group***	**Outcome**	**Sample size: intervention group**	**Sample size: control group**	**Estimate (95% CI/ p value/ SD)**	**Estimate adjusted for**
			science/translational research during residency				
	Graduates after residency programme transition	Graduates before residency programme transition	Percentage of graduates whose current practice included clinical research	19	9	79% vs 0% (p<0.001)	
	Graduates after residency programme transition	Graduates before residency programme transition	Percentage of graduates whose current practice included laboratory research	19	9	5% vs 0% (p=1)	
	Graduates after residency programme transition	Graduates before residency programme transition	Percentage of graduates whose current practice included academic practice	19	9	44% vs 0% (p=0.026)	
Sweeny (2019)[47]	After programme implementation participants	Before programme implementation participants	Number of research active emergency healthcare providers	NR	NR	181 vs 23 (NR)	Unadjusted
	After programme implementation participants	Before programme implementation participants	Number of presentations by emergency clinicians	NR	NR	61 vs 6 (NR)	
**Retention & Promotion**							
Chang (2016)[21]	Attendants from 1 of 3 career development programmes (all female)	Matched non-participants (all female)	Retention as assistant professor role (since first year of appointment)	732	4962	Log rank chi-squared 640.95, df=1 (p<0.001)	Adjusted for years in academic medicine, age, tenure track status, degree type, department type
	Attendants from 1 of 3 career development programmes (all female)	Matched non-participants (all female)	Likelihood of departure from academic medicine - *just assistant professors*	Unclear	Unclear	HR 0.85 (95% CI: 0.74-0.98)	

**First author (Year of publication)**	**Intervention group***	**Control group***	**Outcome**	**Sample size: intervention group**	**Sample size: control group**	**Estimate (95% CI/ p value/ SD)**	**Estimate adjusted for**
	Attendants from 1 of 3 career development programmes (all female)	Matched non-participants (all female)	Likelihood of departure from academic medicine - *just associate professors*	Unclear	Unclear	HR 0.76 (95% CI: 0.64-0.93)	
	Attendants from 1 of 3 career development programmes (all female)	Matched non-participants (all female)	Likelihood of departure from academic medicine - *just full professors*	Unclear	Unclear	HR 0.68 (95% CI: 0.50-0.92)	
	Attendants from 1 of 3 career development programmes (all female)	Matched non-participants (all female)	Departure from academic medicine after 10 years - *just assistant professors*	732	4962	20% vs 33% (NR)	
	Attendants from 1 of 3 career development programmes (all female)	Matched non-participants (all female)	Departure from academic medicine after 10 years - *just associate professors*	429	884	10% vs 17% (NR)	
	Attendants from 1 of 3 career development programmes (all female)	Matched non-participants (all female)	Departure from academic medicine after 10 years - *just full professors*	215	172	8% vs 17% (NR)	
	Attendants from 1 of 3 career development programmes (all female)	Matched non-participants (all female)	Long term retention in academic medicine - *just assistant professors*	732	4962	Intervention participants were less likely to leave academia in the periods 0-13 years after their appointment and	

First author (Year of publication)	Intervention group*	Control group*	Outcome	Sample size: intervention group	Sample size: control group	Estimate (95% CI/ p value/ SD)	Estimate adjusted for
						22 years after or longer (NR).	
	Attendants from 1 of 3 career development programmes (all female)	Matched non-participants (all female)	Long term retention in academic medicine - *just associate professors*	429	884	Intervention participants were less likely to leave academia in the periods 0-10 years after their appointment (NR)	
	Attendants from 1 of 3 career development programmes (all female)	Matched non-participants (all female)	Long term retention in academic medicine - *just full professors*	215	172	Intervention participants were less likely to leave academia in any period after their initial appointment (NR)	
Daley (2006)[22]	Junior faculty after implementation - intervention participants	Junior faculty before implementation - non-intervention participants	Percentage of URM junior staff retained at the faculty	15	12	87% vs 58% (z statistic=1.69, p=0.091)	Unadjusted
	Junior faculty after implementation - intervention participants	Junior faculty before implementation - non-intervention participants	Percentage of URM junior staff retained in academic medicine	15	12	93% vs 75% (z statistic=1.33, p=0.184)	
Ehlers (2018)[24]	Programme participants	Matched non-programme participants	Enter private practice post-programme	70	70	40% vs 38.6% (p=0.862)	Time since matriculation into fellowship was included as a covariate. Matching criteria included fellowship program (CV or GI), gender, years of post-MD graduate training (±1 year for 65 pairs and ±3 years for 5 pairs), age at the time of starting fellowship
	Programme participants	Matched non-programme participants	Enter academic practice post-programme	70	70	60% vs 61.4% (p=0.862)	

First author (Year of publication)	Intervention group*	Control group*	Outcome	Sample size: intervention group	Sample size: control group	Estimate (95% CI/ p value/ SD)	Estimate adjusted for
							training (±3 years), and site of fellowship (2 pairs needed to be matched across sites)
Emans (2008)[25]	Faculty 3 years after implementation	Faculty 3 years before implementation	Number of promotions - *male faculty only*	Unclear	Unclear	17% increase (NR)	Unadjusted
	Faculty 3 years after implementation	Faculty 3 years before implementation	Number of promotions - *female faculty only*	Unclear	Unclear	56% increase (NR)	Unadjusted
	Faculty 3 years after implementation	Faculty 3 years before implementation	Number of promotions - *URM faculty only*	Unclear	Unclear	60% increase (NR)	Unadjusted
	Faculty 3 years after implementation	Faculty 3 years before implementation	Number of promotions - *Asian faculty only*	Unclear	Unclear	60% increase (NR)	Unadjusted
Guevara (2018)[28]	Interview applications scholars	Interview applications non-scholars	Percentage of people who received any kind of position promotion	76	48	67.1% vs 58.3% (p=0.32)	Adjusted for age, gender, race/ethnicity, application year, change of institutions, and rank at time of application
	Interview applications scholars	Interview applications non-scholars	Retention in academic position (% of people)	76	48	84.2% vs 75% (p=0.21)	
Kohlwes (2006)[32]	Programme residents (PRIME)	Other program residents (UCSF internal medicine programs)	Percentage of people asked to be a chief resident	32	185	21.8% vs 9.2% (p=0.03)	Unadjusted
Kohlwes (2016)[33]	Programme residents (PRIME)	Non-PRIME programme residents	Percentage of alumni with an academic appointment	71	98	71.4% vs 67% (chi-squared=0.370, p=0.5430	Unadjusted
	Programme residents (PRIME)	Non-PRIME programme residents	Influence of research experience during residency on career choice (% strongly agree	71	98	63.4% vs 46.4% (chi-squared=4.757, p=0.029)	

First author (Year of publication)	Intervention group*	Control group*	Outcome	Sample size: intervention group	Sample size: control group	Estimate (95% CI/ p value/ SD)	Estimate adjusted for
			or agree on questionnaire)				
	Programme residents (PRIME)	Non-PRIME programme residents	Influence of success with research during residency on career choice (% strongly agree or agree on questionnaire)	71	98	36.6% vs 23.5% (chi-squared=3.458, p=0.063)	
Ockene (2017)[38]	Programme participants	Non-programme participants	Percentage of people promoted to associate professor	29	188	72% vs 32% (p<0.001)	Adjusted for equivalent time in rank
Ries (2009)[40]	NCLAM programme participants	Non-NCLAM programme participants hired during same period	Faculty retention after 8-year probationary period (univariate analysis)	120	719	HR=1.77 (95% CI: 1.20-2.61, p=0.004)	Unadjusted
	NCLAM programme participants	Non-NCLAM programme participants hired during same period	Faculty retention after 8-year probationary period (multivariate analysis)	120	719	HR = 1.67 (95% CI: 1.11-2.50, p=0.01)	Date of hire, gender and URM faculty were included as covariates
Ries (2012)[41]	NCLAM programme participants (1999-2006)	Matched non-NCLAM programme participants (1999-2006)	8-year probationary period retention rate (% of people retained)	113	202	67% vs 56% (p=0.04)	Matched for: (1) gender, (2) academic series (research versus clinical primary job description), (3) initial academic rank/step (academic experience when hired), (4) hire date (within 12 months, to control for temporal changes in internal and external institutional variables), and (5) department
**Recruitment**							
Brandt (2018)[19]	Research residents	Clinical residents	Percentage of people who entered first job in academic practice	43	284	34% vs 20% (p=0.0001)	Unadjusted
	Research residents	Clinical residents (before	Percentage of people	43	87	20% vs 14% (NR)	Unadjusted

**First author (Year of publication)**	**Intervention group***	**Control group***	**Outcome**	**Sample size: intervention group**	**Sample size: control group**	**Estimate (95% CI/ p value/ SD)**	**Estimate adjusted for**
	(after implementing programme)	implementing programme)	who currently or once held a full-time academic appointment				
Dannels (2008)[23]	Programme participants	Two control groups: Applied but not accepted non-programme participants & general mid-career faculty women (also non-participants)	Highest rank/ position professor	53	25 & 172	69.8% vs 48% vs 68.6% (NR)	Unadjusted but faculty control group matched on academic rank, department chair status, race/ethnicity, discipline, degree type, basic sciences vs clinical department, age, medical school ownership, and awards ranking.
	Programme participants	Two control groups: Applied but not accepted non-programme participants & general mid-career faculty women (also non-participants)	Highest rank/ position department chair/ director	52	27 & 160	25% vs 18.5% vs 15.6% (NR)	
	Programme participants	Two control groups: Applied but not accepted non-programme participants & general mid-career faculty women (also non-participants)	Highest rank/ position dean and above	52	27 & 160	11.5% vs 11.1% vs 4.4% (NR)	
	Programme participants	Two control groups: Applied but not accepted non-programme participants & general mid-career faculty women (also non-participants)	Academic title of chair or above	52	27 & 160	63.5% vs 37.0% vs 22.5% (chi-squared=29.96, p<0.001)	
Emans (2008)[25]	Faculty 5 years	Faculty year of	Percentage of professors	Unclear	Unclear	12% vs 14% (NR)	Unadjusted

First author (Year of publication)	Intervention group*	Control group*	Outcome	Sample size: intervention group	Sample size: control group	Estimate (95% CI/ p value/ SD)	Estimate adjusted for
	after programme implementation	programme implementation	- *female only*				
	Faculty 5 years after programme implementation	Faculty year of programme implementation	Percentage of associate professors - *female only*	Unclear	Unclear	30% vs 21% (p=0.023)	
	Faculty 5 years after programme implementation	Faculty year of programme implementation	Percentage of assistant professors - *female only*	Unclear	Unclear	44% vs 34% (NR)	
	Faculty 5 years after programme implementation	Faculty year of programme implementation	Percentage of instructors - *female only*	Unclear	Unclear	58% vs 53% (NR)	
	Faculty 5 years after programme implementation	Faculty year of programme implementation	Percentage of professors - *URM only*	Unclear	Unclear	3% vs 3% (NR)	
	Faculty 5 years after programme implementation	Faculty year of programme implementation	Percentage of associate professors - *URM only*	Unclear	Unclear	3% vs 2% (NR)	
	Faculty 5 years after programme implementation	Faculty year of programme implementation	Percentage of assistant professors - *URM only*	Unclear	Unclear	4% vs 5% (NR)	
	Faculty 5 years after programme implementation	Faculty year of programme implementation	Percentage of instructors - *URM only*	Unclear	Unclear	8% vs 8% (NR)	
	Faculty 5 years after programme implementation	Faculty year of programme implementation	Percentage of professors - *Asian only*	Unclear	Unclear	2% vs 0% (NR)	
	Faculty 5 years after programme implementation	Faculty year of programme implementation	Percentage of associate professors - *Asian only*	Unclear	Unclear	6% vs 3% (p=0.023)	
	Faculty 5 years after programme	Faculty year of programme	Percentage of assistant professors - *Asian only*	Unclear	Unclear	16% vs 9% (p=0.001)	

First author (Year of publication)	Intervention group*	Control group*	Outcome	Sample size: intervention group	Sample size: control group	Estimate (95% CI/ p value/ SD)	Estimate adjusted for
	implementation	implementation					
	Faculty 5 years after programme implementation	Faculty year of programme implementation	Percentage of instructors - *Asian only*	Unclear	Unclear	12% vs 12% (NR)	
Goldenberg (2012)[26]	Programme trainees (academic practice physicians only)	Non-programme trainees	Time from fellowship entry to assistant professor	11	9	Median 3.5 years vs 7 years (p<0.001)	Unadjusted
Joshua Smith (2014)[30]	Two intervention groups: Residents with protected research time and completing degree, & residents with just protected research time	Residents without protected research time	Percentage of residents for which their first job is an academic appointment	15 & 24	23	93.3% vs 58.3% vs 30.4% (p=0.046 comparing research time, p<0.001 comparing no research time)	Unadjusted
Khot (2011)[31]	NIH associates who entered academic medicine	Non-associates who entered academic medicine (identified from faculty roster database)	Academic rank achieved - full professor	1577	27821	Ratio 1.57 (95% CI: 1.49-1.66, p<0.001)	Unadjusted
	NIH associates who entered academic medicine	Non-associates who entered academic medicine (identified from faculty roster database)	Academic rank achieved - chair	1577	27821	Ratio 2.0 (95% CI: 1.78-2.24, p<0.001)	
	NIH associates who entered academic medicine	Non-associates who entered academic medicine (identified from faculty roster database)	Academic rank achieved - dean	1577	27821	Ratio 2.97 (95% CI: 2.21-3.98, p<0.001)	

First author (Year of publication)	Intervention group*	Control group*	Outcome	Sample size: intervention group	Sample size: control group	Estimate (95% CI/ p value/ SD)	Estimate adjusted for
	NIH associates who entered academic medicine	Non-associates who entered academic medicine (identified from faculty roster database)	Active faculty appointments in the top 10 schools	626	6038	Ratio 1.34 (95% CI: 1.15-1.56)	
	NIH associates who entered academic medicine	Non-associates who entered academic medicine (identified from faculty roster database)	Active faculty appointments in the top 20 schools	626	6038	Ratio 1.47 (95% CI: 1.33-1.63)	
Sheridan (2010)[42]	Intervention-participating departments	Non-intervention participating departments	Percentage of female faculty hired before and after workshop implementation	17	9	Precise estimate not reported - see Figure 1 in paper for details (p<0.05)	Unadjusted
Sweeny (2019)[47]	After programme implementation participants	Before programme implementation participants	Number of new principal investigators recruited	NR	NR	55 vs 17 (NR)	Unadjusted
Valantine (2014)[43]	University faculty where programme was implemented	Faculty at 6 peer universities	Change in female faculty as a percentage of total faculty; *including assistant, associate, and full professors* (2004-2010)	1219	17000	5.8% vs 4.5% (p=0.011)	Unadjusted
	University faculty where programme was implemented	Faculty at 6 peer universities	Change in female faculty as a percentage of total faculty; *just assistant professors* (2004-2010)	420	7362	4.7% vs 5.0% (p=0.573)	
	University faculty where programme was implemented	Faculty at 6 peer universities	Change in female faculty as a percentage of total faculty; *just associate professors* (2004-2010)	370	4546	5.8% vs 5.1% (p=0.385)	

First author (Year of publication)	Intervention group*	Control group*	Outcome	Sample size: intervention group	Sample size: control group	Estimate (95% CI/ p value/ SD)	Estimate adjusted for
	University faculty where programme was implemented	Faculty at 6 peer universities	Change in female faculty as a percentage of total faculty; *just full professors* (2004-2010)	429	5092	5.2% vs 4.0% (p=0.003)	
	University faculty where programme was implemented	National faculty cohort	Change in female faculty as a percentage of total faculty; *including assistant, associate, and full professors* (2004-2010)	1219	116996	5.8% vs 4.0% (p=0.001)	Unadjusted
	University faculty where programme was implemented	National faculty cohort	Change in female faculty as a percentage of total faculty; *just assistant professors* (2004-2010)	420	56509	4.7% vs 3.6% (p=0.340)	
	University faculty where programme was implemented	National faculty cohort	Change in female faculty as a percentage of total faculty; *just associate professors* (2004-2010)	370	27842	5.8% vs 4.1% (p=0.868)	
	University faculty where programme was implemented	National faculty cohort	Change in female faculty as a percentage of total faculty; *just full professors* (2004-2010)	429	31645	5.2% vs 3.7% (p=0.001)	
**Publications**							
Brandt (2018)[19]	Research residents	Clinical residents	Publication productivity over career	43	284	Median 14 vs 4 (p<0.0001)	Unadjusted
	Research residents	Clinical residents	Publication productivity during residency	43	284	Median 10 vs 2.5 (p<0.0001)	
Ehlers (2018)[24]	Programme participants	Matched non-programme participants	Number of publications at 5-years post-fellowship	70	70	Median 8 vs 5 (p=0.041)	Matching criteria included fellowship program (CV or GI), gender, years of post-MD graduate training (±1 year for 65
	Programme participants	Matched non-programme participants	Number of first-author publications at 5-years	70	70	Median 4 vs 2 (p=0.002)	

First author (Year of publication)	Intervention group*	Control group*	Outcome	Sample size: intervention group	Sample size: control group	Estimate (95% CI/ p value/ SD)	Estimate adjusted for
			post-fellowship				pairs and ±3 years for 5 pairs), age at the time of starting fellowship training (±3 years), and site of fellowship (2 pairs needed to be matched across sites)
	Programme participants	Matched non-programme participants	H-Index (as of year when studies was conducted - January 2018)	70	70	Median 11 vs 7 (p=0.013)	Time since matriculation into fellowship was included as a covariate. Matching criteria included fellowship program (CV or GI), gender, years of post-MD graduate training (±1 year for 65 pairs and ±3 years for 5 pairs), age at the time of starting fellowship training (±3 years), and site of fellowship (2 pairs needed to be matched across sites)
Goldenberg (2012)[26]	Programme trainees	Non-programme trainees	Annual number of peer-reviewed publications 3-4 years post-fellowship	11	9	Median 3.5 vs 1 (p=0.01)	Unadjusted
Grisso (2017)[27]	Intervention group	Control group (no intervention)	Increase in total no. of publications 2009 to 2012	62	70	Rate Ratio 0.80 (95% CI: 0.63-1.02, p=0.07)	Statistical tests were adjusted to account for correlation induced by clustered design using generalized estimating equations. Both within-person factors (age, years in rank, race) and unit-level factors (intervention assignment) were modelled simultaneously
	Intervention group	Control group (no intervention)	Increase in first author publications 2009 to 2012	62	70	Rate Ratio 1.00 (95% CI: 0.67-1.50, p=0.99)	
	Intervention group	Control group (no intervention)	Increase in total no. of peer review publications 2009 to 2012	62	70	Rate Ratio 0.95 (95% CI: 0.68-1.33, p=0.78)	
	Intervention group	Control group (no intervention)	Increase in first author peer review publications 2009 to 2012	62	70	Rate Ratio 1.06 (95% CI: 0.58-1.95, p=0.85)	

First author (Year of publication)	Intervention group*	Control group*	Outcome	Sample size: intervention group	Sample size: control group	Estimate (95% CI/ p value/ SD)	Estimate adjusted for
Guevara (2018)[28]	Interview applications scholars	Interview applications non-scholars	Total no. of publications (mean) from point of application to December 2013	76	48	27.2 vs 33.0 (p=0.47, SD=30.0 vs 58.8)	Adjusted for age, gender, race/ethnicity, application year, change of institutions, and rank at time of application
	Interview applications scholars	Interview applications non-scholars	H-Index (as of December 2013)	76	48	12.5 vs 10.9 (p=0.32, SD=7.9 vs 10.2)	
Harrison (2020)[29]	Participants after intervention implementation	Participants before intervention implementation	Mean number of publications (in 5 years) - *residents only*	Unclear	Unclear	7.0 vs 4.4 (p=0.263, SD= 4.47 vs 1.82)	Unadjusted
	Participants after intervention implementation	Participants before intervention programme	Mean number of publications (in 5 years) - *faculty only*	Unclear	Unclear	16.6 vs 12.8 (p=0.197, SD=4.39 vs 4.15)	Unadjusted
Joshua Smith (2014)[30]	Two intervention groups: Residents with protected research time and completing degree, & residents with just protected research time	Residents without protected research time	Number of publications (mean)	17 & 27	24	10.3 vs 5.30 vs 1.29 (p=0.001 comparing to research time, p<0.001 comparing to no research time)	Unadjusted
	Two intervention groups: Residents with protected research time and completing degree, & residents with just protected research time	Residents without protected research time	Number of first-author publications (mean)	17 & 27	24	4.06 vs 2.30 vs 0.46 (p=0.017 comparing to research time, p<0.001 comparing to no research time)	
	Two intervention groups: Residents with protected	Residents without protected research time	Impact factor of publications (mean)	17 & 27	24	32.3 vs 17.8 vs 2.69 (p=0.001 comparing to	

First author (Year of publication)	Intervention group*	Control group*	Outcome	Sample size: intervention group	Sample size: control group	Estimate (95% CI/ p value/ SD)	Estimate adjusted for
	research time and completing degree, & residents with just protected research time					research time, p<0.001 comparing to no research time)	
	Two intervention groups: Residents with protected research time and completing degree, & residents with just protected research time	Residents without protected research time	Adjusted impact factor of publications (mean)	17 & 27	24	65.4 vs 36.1 vs 5.17 (p=0.005 comparing to research time, p<0.001 comparing to no research time)	Adjusted for level of authorship
Klimas (2017)[45]	Programme participants	Non-programme participants	Total number of published papers at the end of the one-year fellowship	4	4	7 vs 1 (p=0.1)	Unadjusted
Kohlwes (2016)[33]	Programme residents	Non-programme residents	Percentage of alumni who published research they had started during residency (first-author peer-review article)	71	92	64.3% vs 40.2% (chi-squared=9.213, p=0.002)	Unadjusted
Löwe (2008)[48]	Intervention residents	Control residents (from two different locations)	Percentage of people who completed one or more original publications with first authorship in the last year	15	22	46.7% vs 22.7% (p=0.13)	Unadjusted
	Intervention residents	Control residents (from two different locations)	Percentage of people who completed one or more original publications with co-authorship in the last year	15	22	60% vs 18.2% (p=0.01)	

First author (Year of publication)	Intervention group*	Control group*	Outcome	Sample size: intervention group	Sample size: control group	Estimate (95% CI/ p value/ SD)	Estimate adjusted for
	Intervention residents	Control residents (from two different locations)	Percentage of people who completed one or more review or meta-analysis with first authorship in the last year	15	22	0% vs 9.1% (p=0.5)	
	Intervention residents	Control residents (from two different locations)	Percentage of people who completed one or more review or meta-analysis with co-authorship in the last year	15	22	0% vs 9.1% (p=0.5)	
	Intervention residents	Control residents (from two different locations)	Percentage of people who completed one or more book article with first authorship in the last year	15	22	26.7% vs 18.2% (p=0.69)	
	Intervention residents	Control residents (from two different locations)	Percentage of people who completed one or more book article with co-authorship in the last year	15	22	20% vs 4.6% (p=0.28)	
Merani (2014)[46]	Additional degree trainees: Masters trainees, & PhD trainees	Clinical-only trainees	Percentage of surgeons involved in research publication during training with any authorship role	72 & 33	218	81.9% vs 100% vs 38.1% (p<0.05)	Unadjusted
	Additional degree trainees: Masters trainees, & PhD trainees	Clinical-only trainees	Percentage of surgeons involved in research publication during training with principal author role	72 & 33	218	58.3% vs 100% vs 27.1% (p<0.05)	
	Additional degree trainees: Masters	Clinical-only trainees	Percentage of surgeons involved in research	72 & 33	218	19.4% vs 12.1% vs 5% (p<0.05)	

First author (Year of publication)	Intervention group*	Control group*	Outcome	Sample size: intervention group	Sample size: control group	Estimate (95% CI/ p value/ SD)	Estimate adjusted for
	trainees, & PhD trainees		publication during training with senior author role				
	Additional degree trainees: Masters trainees, & PhD trainees	Clinical-only trainees	Percentage of surgeons involved in research publication post-training with any authorship role	69 & 32	201	81.2% vs 90.6% vs 44.3% (p<0.05)	
	Additional degree trainees: Masters trainees, & PhD trainees	Clinical-only trainees	Percentage of surgeons involved in research publication post-training with principal author role	69 & 32	201	56.5% vs 71.9% vs 26.4% (p<0.05)	
	Additional degree trainees: Masters trainees, & PhD trainees	Clinical-only trainees	Percentage of surgeons involved in research publications post-training with senior author role	69 & 32	201	37.7% vs 46.9% vs 14.9% (p<0.05)	
Mills (2011)[36]	Programme participants	Non-programme participants	Percentage of participants with publication after graduation	232	295	69% vs 34% (p<0.001)	Unadjusted
	Programme participants	Non-programme participants	Median number of publications after graduation	232	295	2 vs 0 (p<0.001)	
	Programme participants	Non-programme participants	Likelihood of publishing in the future	232	295	OR=3.7 (95% CI: 2.5-5.6)	Adjusted for gender and publications prior to programme
Nasab (2019)[37]	Programme participants	Non-programme participants (before implementation of programme)	Number of published papers during course period (2015-2018) - *just junior faculty members*	14	26	Mean 9.21 vs 4 (p=0.003, SD=8.1 vs. 5.21)	Unadjusted
	Programme participants	Non-programme participants (before implementation of	Number of published papers during course period (2015-2018) - *just*	35	11	Mean 4.31 vs 2 (p=0.008, SD=3.44 vs. 1.67)	

First author (Year of publication)	Intervention group*	Control group*	Outcome	Sample size: intervention group	Sample size: control group	Estimate (95% CI/ p value/ SD)	Estimate adjusted for
		programme)	*fellows*				
	Programme participants	Non-programme participants (before implementation of programme)	Journal impact factor during course period (2015-2018) - *just junior faculty members*	14	26	Mean 3.09 vs 4.57 (p=0.67, SD=1.54 vs. 5.03)	
	Programme participants	Non-programme participants (before implementation of programme)	Journal impact factor during course period (2015-2018) - *just fellows*	35	11	Mean 5.46 vs 3.03 (p=0.776, SD=9.81 vs. 1.72)	
Patel (2018)[39]	Programme cohorts (2011/2012 - 2014/2015)	Pre-programme cohorts (2007/2008 - 2010/2011)	Likelihood of publishing one or more clinical research articles (%)	35	36	77% vs 44% (chi-squared=7.9, p=0.005)	Unadjusted
	Programme cohorts (2011/2012 - 2014/2015)	Pre-programme cohorts (2007/2008 - 2010/2011)	Number of published clinical research articles during residency (mean)	35	36	2.09 vs 1.56 (NR)	

*Groups are reported as described by authors. **Inconsistent data reported and referred to in tables and text; data represented here is extracted from text.

Abbreviations: BCRP - Boston Combined Residency Programme; CCWAS - Culture that is conducive to women's academic success; NCLAM - National Center of Leadership in Academic Medicine; NR - Not Reported; PRIME - Primary Medical Education programme; SWIF - Strain-based work interference with family; TWIF - Time-based work interference with family; UCSF - University of California San Francisco; URM - Underrepresented Minorities.
