# Supplementary material for: Strengthening the clinical academic pathway: a systematic review of interventions to support clinical academic careers for doctors and dentists
Source: BMJ Open. 2022 Sep 8;12(9):e060281. doi: 10.1136/bmjopen-2021-060281 (PMC9462120; doi:10.1136/bmjopen-2021-060281)
Supplement: Author's manuscript [file bmjopen-2021-060281.draft_revisions.pdf]

BMJ Open is committed to open peer review. As part of this commitment we make the peer review history of every article we publish publicly available.

When an article is published we post the peer reviewers' comments and the authors' responses online. We also post the versions of the paper that were used during peer review. These are the versions that the peer review comments apply to.

The versions of the paper that follow are the versions that were submitted during the peer review process. They are not the versions of record or the final published versions. They should not be cited or distributed as the published version of this manuscript.

BMJ Open is an open access journal and the full, final, typeset and author-corrected version of record of the manuscript is available on our site with no access controls, subscription charges or pay-per-view fees (http://bmjopen.bmj.com).

If you have any questions on BMJ Open's open peer review process please email
info.bmjopen@bmj.com

BMJ Open

Strengthening the clinical academic pathway: a systematic review of interventions to support clinical academic careers for doctors and dentists

Journal:	*BMJ Open*
Manuscript ID	bmjopen-2021-060281
Article Type:	Original research
Date Submitted by the Author:	17-Dec-2021

Complete List of Authors:	Raine, Gary; University of York, Centre for Reviews and Dissemination<br>Evans, Connor; University of York, Centre for Reviews and Dissemination<br>Uphoff, Eleonora; University of York, Centre for Reviews and Dissemination<br>Brown, Jennifer ; University of York, Centre for Reviews and Dissemination; University of York, Mental Health and Addiction Research Group, Department of Health Sciences,<br>Crampton, Paul; Hull York Medical School, Health Professions Education Unit<br>Kehoe, Amelia; Hull York Medical School, Health Professions Education Unit<br>Stewart, Lesley; University of York, Centre for Reviews and Dissemination<br>Finn, Gabrielle; Manchester University, School of Medical Sciences<br>Morgan, Jessica; University of York, Centre for Reviews and Dissemination; Leeds Children's Hospital, Department of Paediatric Haematology & Oncology

Keywords:	EDUCATION & TRAINING (see Medical Education & Training), MEDICAL EDUCATION & TRAINING, HEALTH SERVICES ADMINISTRATION & MANAGEMENT

SCHOLARONE™
Manuscripts

I, the Submitting Author has the right to grant and does grant on behalf of all authors of the Work (as defined in the below author licence), an exclusive licence and/or a non-exclusive licence for contributions from authors who are: i) UK Crown employees; ii) where BMJ has agreed a CC-BY licence shall apply, and/or iii) in accordance with the terms applicable for US Federal Government officers or employees acting as part of their official duties; on a worldwide, perpetual, irrevocable, royalty-free basis to BMJ Publishing Group Ltd ("BMJ") its licensees and where the relevant Journal is co-owned by BMJ to the co-owners of the Journal, to publish the Work in this journal and any other BMJ products and to exploit all rights, as set out in our licence.

The Submitting Author accepts and understands that any supply made under these terms is made by BMJ to the Submitting Author unless you are acting as an employee on behalf of your employer or a postgraduate student of an affiliated institution which is paying any applicable article publishing charge ("APC") for Open Access articles. Where the Submitting Author wishes to make the Work available on an Open Access basis (and intends to pay the relevant APC), the terms of reuse of such Open Access shall be governed by a Creative Commons licence – details of these licences and which Creative Commons licence will apply to this Work are set out in our licence referred to above.

Other than as permitted in any relevant BMJ Author's Self Archiving Policies, I confirm this Work has not been accepted for publication elsewhere, is not being considered for publication elsewhere and does not duplicate material already published. I confirm all authors consent to publication of this Work and authorise the granting of this licence.

For peer review only

Strengthening the clinical academic pathway: a systematic review of interventions to support clinical academic careers for doctors and dentists

Gary Raine[1], Connor Evans[1], Eleonora Uphoff[1], Jennifer V E Brown[1,2], Paul E S Crampton[3], Amelia Kehoe[3], Lesley A Stewart[1], Gabrielle M Finn[4], and Jessica E Morgan[1,5]

Affiliations

1. Centre for Reviews and Dissemination, University of York, York, UK, YO10 5DD
2. Mental Health and Addiction Research Group, Department of Health Sciences, University of York, York, UK, YO10 5DD
3. Health Professions Education Unit, Hull York Medical School, University of York, York, UK, YO10 5DD
4. Division of Medical Education, School of Medical Sciences, Faculty of Biology, Medicine and Health, The University of Manchester, M13 9NT
5. Department of Paediatric Haematology & Oncology, Leeds Children's Hospital, Leeds, UK, LS1 3EX

Corresponding author: Jessica E Morgan, Centre for Reviews and Dissemination, University of York, York, UK, YO10 5DD, email: jess.morgan@york.ac.uk

Word count: 5268

For peer review only

Structured abstract

Objective:

Evaluate existing evidence on interventions intended to increase recruitment and retention to clinical academic (CA) careers, with a focus on addressing inequalities.

Design: Systematic review.

Data sources: MEDLINE, Embase, Cochrane Controlled Register of Trials (CENTRAL), PsycINFO, and Education Resource Information Center (ERIC) searched October 2019.

Study selection: Eligible studies included qualified doctors, dentists and/or those with a supervisory role. Outcomes were defined by studies and related to success rates of joining or continuing within a CA career.

Data extraction and synthesis: Abstract screening was supported by machine learning software. Full text screening was performed in duplicate, and risk of bias assessed. Narrative synthesis of quantitative data was performed. Qualitative data were thematically analysed.

Results:

148 studies included in the general overview, 28 in the quantitative synthesis, 17 in the qualitative synthesis, and two in both. Studies lacked methodological rigour and/or were hindered by incomplete reporting. Most were from North America. No study included in the syntheses evaluated interventions aimed at CA dentists.

Most quantitative evidence was from multi-faceted training programmes. These may increase recruitment, but findings were less clear for retention and other outcomes. Qualitative studies reported benefits of supportive relationships, including peers and senior mentors. Protected time for research helped manage competing demands on CAs. Committed and experienced staff were seen as key facilitators of programme success. Respondents identified several other factors at a programme, organisational or national level which acted as facilitators or barriers to success. Few studies reported on the effects of interventions specific to women or minority groups.

Conclusions:

Existing research is limited by rigour and reporting. Better evaluation of future interventions, particularly those intended to address inequalities, is required. Within the limits of the evidence, comprehensive multi-faceted programmes of training, including protected time, relational and support aspects, appear most successful in promoting CA careers.

Systematic Review Registration: Open Science Framework: https://osf.io/mfy7a

Strengths and limitations of this study

- This was a rigorous, systematic, and transparent review conducted by a highly experienced research team.
- Machine learning methodology facilitated very high-volume title and abstract screening to identify the most relevant records earlier.
- Limitations in the reporting of the existing literature made synthesis challenging.
- It is unclear to what extent findings, which derived mostly from studies conducted in the USA, can be applied to other contexts. Multiple factors, including inter-country differences in organisational structures and practices, potentially limit the generalisability of findings.

For peer review only

Introduction

Clinical academics, individuals who work in both clinical and research roles, are a key part of the academic and healthcare workforce, combining expertise from both roles for the benefit of patients. The proportion of clinicians who choose a clinical academic career has fallen over time.[1] Furthermore, there are inequalities within this career path – with both gender and ethnicity differences being more pronounced than in either clinical or academic settings alone.[2 3] Thus, interventions are needed which both increase the numbers of clinical academics and facilitate increased equality of opportunity for those who wish to pursue this career.

A previous systematic review (searches up to 2017) summarised quantitative evaluations of interventions to improve gender equality in any academic discipline.[4] It found that the evidence base was restricted by poor quality, and that interventions were limited to approaches that required time and effort from the women they intended to support ('bottom-up' approaches).[4]

Career pathways for clinical academics frequently separate doctors and dentists from other members of the healthcare workforce. Therefore, our systematic review aimed to identify, critically appraise, and synthesise research on existing interventions to increase recruitment, retention, and career progression in clinical academic medicine and dentistry. Whilst we evaluated interventions focused on gender equality, we also sought interventions to address inequalities related to characteristics other than gender. Our research aims to inform regulators and funders of the most effective interventions to support and promote clinical academic careers, with a view to increasing recruitment and retention within this key group of healthcare professionals. Clinical academics may also use our findings to negotiate their roles and advocate for support from those providing oversight to their careers.

Methods

The systematic review protocol was registered (https://osf.io/mfy7a) and published.[5]

Search and information sources

The following databases were searched: MEDLINE (including MEDLINE Epub Ahead of Print, MEDLINE In-Process & Other Non-Indexed Citations, and MEDLINE Daily), Embase, Cochrane Controlled Register of Trials (CENTRAL), PsycINFO, and Education Resource Information Center (ERIC). Subject headings and free-text terms were used. Searches were limited to human studies published in English, from 2004 onwards (the introduction of the Athena SWAN initiative, a high-profile national programme aiming to improve gender equality across higher education).

We conducted two searches. One broad, sensitive search including terms relating to clinical academics. The other search was more specific and identified a subset of records from the broad search, by using terms relating to clinical academics and career development, recruitment, retention, and attrition. Details about the search process and the performance of the two searches will be reported separately.

Inclusion and exclusion criteria

We included studies of qualified doctors, dentists and/or those with a supervisory role over these careers.[5] Studies of medical and dental students were not included. We included quantitative and qualitative studies evaluating interventions to increase recruitment to, and improve retention in, clinical academic careers, using study-defined outcome measures relating to rates of joining or continuing within clinical academia. Conference abstracts were excluded. Studies were limited to those performed in high-income countries, according to the World Bank classification,[6] in recognition of the cultural and organisational setting in which the research findings are to be applied.

Given the large numbers of potentially relevant studies identified during the screening process, additional exclusion criteria were introduced at the full-text screening stage to focus on the most relevant evidence. We excluded studies published before 2005, studies where the majority of the data was collected before 2004, and studies conducted in high-income countries with considerable differences in culture and/or health care

For peer review only

provision compared with the UK, for example Singapore. The analysis plan was adjusted to enable a time and resource efficient reporting process.

Study selection and data extraction

We used the systematic review software Rayyan[7] to support the study selection process and employed our two-staged search process to train the built-in machine learning-based prioritisation function, with a view to more rapidly identifying potentially relevant records. Title and abstract screening took place in three stages and stopped once the rate of potentially eligible records identified by the machine learning algorithm had fallen sufficiently from baseline.[8]

Full-text screening was undertaken independently and in duplicate. At all stages, disagreements were resolved by a third reviewer or in team discussions.

Data were extracted by one researcher using standardised data extraction forms and independently checked by a second researcher. We extracted outcome data for only those studies that reported quantitative data and included a control group.

Only quantitative studies with a control group and qualitative studies using data derived predominantly from verbal data collection methods were included in a detailed synthesis of data.

Quality assessment

Quality assessment of studies included in the synthesis used the Cochrane risk of bias tool for RCTs,[9] the Newcastle-Ottawa scale for non-randomised studies,[10] the Qualitative Assessment and Review Instrument Checklist for qualitative studies,[11] the Mixed Methods Appraisal Tool for mixed methods studies,[12] and the RAMESES II Quality Standards for Realist Evaluation.[13] Each study was individually assessed and checked by a second reviewer, and any conflicts resolved via discussion.

Data Synthesis

Data were summarised in narrative and tabular form. For accuracy of reporting, we retained the terminology used by included papers to describe their participants (which most often uses US terms). The frequently used American academic ranks of assistant professor and associate professor approximate those of lecturer and senior lecturer, respectively, in the UK.

Due to the heterogeneous nature of the studies, quantitative data were synthesised narratively, and qualitative data were synthesised based on the principles of thematic analysis.[14] All relevant qualitative findings were coded line-by-line by one researcher and codes subsequently reviewed by another researcher. Codes were developed inductively and further refined as appropriate. Findings related to specific codes were brought together to identify cross-cutting themes and issues of potential relevance.

Patient and Public Involvement statement

Through Healthwatch York,[15] a member of the public was involved in the project steering group.

Results

Study selection

Electronic databases were searched in October 2019 and returned a total of 34,230 records; 148 studies examined interventions; of which 28 were included in the synthesis of quantitative data, 17 in the qualitative synthesis, and data from two studies were included in both (Figure 1). The remaining 101 studies were not synthesised further.

For peer review only

Study characteristics

Studies in the quantitative synthesis

Of the 30 studies included in the quantitative synthesis, 26 were conducted in the USA,[16-41] two in Canada,[42 43] one in Australia,[44] and one in Germany.[45] Twenty-three were single centre programmes,[16 17 19-22 24 26 27 29-41 45] and seven were national.[18 23 25 28 42-44]

No study included within the syntheses focused on clinical academic dentists. Otherwise, the populations studied were varied, in terms of grade, academic level and medical background of participants (see Appendix 1). Due to this high degree of variability, it was often difficult to determine the exact population investigated. Fourteen studies focused solely on participants who had completed their medical training,[17-20 22 24 28 30-32 35 38-40] 11 studied just trainees,[16 21 23 26 27 29 33 41-43 45] three included mixed populations,[25 34 36] and two were unclear/not reported.[37 44]

Studies encompassed a diverse range of interventions. The majority evaluated complex interventions involving elements such as mentoring, protected research time, leadership training, and teaching workshops. Academic training programmes tended to focus on advancing trainee academic skills, research productivity and interest, whilst career development programmes (called faculty development programmes in some studies) centred on enhancing junior/senior faculty workforce within clinical academia through promotion, retention, and recruitment.

Four interventions had a gender-focus and were tailored specifically towards women,[18 20 24 40] two towards ethnicity/underrepresented minority faculty in medicine,[19 25] and two towards historically underrepresented faculty including women and minority populations.[22 39]

Studies in the qualitative synthesis

Of the 19 studies included in the qualitative synthesis, 11 were conducted in the USA,[17 25 46-54] five in Canada,[55-59] and three in the UK.[60-62] Eight were from single institutions,[17 51 53-55 57-59] and 11 were national level initiatives.[25 46-50 52 56 60-62] As in the quantitative synthesis, there was considerable diversity in the populations studied and the interventions involved (see Appendix 1).

Two interventions were aimed at women,[49 52] one at individuals from ethnic groups underrepresented in medicine,[25] and another at “busy clinician educators”.[53] One study reported on an intervention targeted at participants who experienced substantial caregiving challenges.[50]

Quality assessment

Both quantitative and qualitative studies lacked methodological rigour and/or were hindered by incomplete reporting. Across all study types, intervention characteristics and population definitions were reported ambiguously. In most cohort studies there was minimal participant matching between intervention and control groups, and participant comparisons were often unadjusted or ill-defined. Group selection was equally problematic as many studies included pre-selected or highly motivated populations to receive the interventions, which may unintentionally bias results in favour of the intervention programmes. Nonetheless, the quantitative literature generally included appropriate follow-up methods and a large volume of outcome data suitable for analysis. The qualitative studies also provided rich data from relevant populations. However, comprehensive analysis plans and complete methodological reporting were not provided in most qualitative studies, and there was little reflexivity demonstrated. For further details on quality assessments, see appendix 2.

Synthesis of quantitative data

All relevant reported outcomes were extracted and then grouped under eight broad categories relating to clinical academic careers: aspiration, career satisfaction, skills & knowledge, research funding, research participation, recruitment, retention/promotion, and publication outcomes (full outcome data are provided in Appendix 3).

For peer review only

Aspiration

Only one study included a measure of aspiration. It found that significantly more participants attending a career development programme aspired to achieve a higher leadership position in academic medicine compared with non-attendees.[20]

Career satisfaction

Three studies reported outcomes relating to career satisfaction.[17 24 41] No single outcome demonstrated a statistically significant benefit for intervention participants versus controls, although results were favourable towards the intervention participants.

Skills and knowledge

Four studies reported outcomes relating to skills and knowledge.[17 29 41 45] Significantly higher clinical competence scores,[29] and improved research competence scores[45] were found among participants of two academic training programmes. Methodological research knowledge also significantly improved for intervention participants in one study.[45] Significantly more intervention participants learned how to give a presentation in the study by Winn et al;[41] yet, within the same study there was no significant difference in the percentage of participants who learned how to present a poster or felt prepared for scholarly work after post-graduate training, compared with controls. No significant benefits were found for the Academy for Collaborative Innovation and Transformation career development programme.[17]

Research Funding

Outcomes relating to research funding were reported in six studies.[23-25 31 44 45] Three studies found significant increases in the number of funded grants[25 31] or the percentage of people with successful grant applications[45] for participants in programmes which combined research training with mentorship. Similar findings were non-significant in two training and development programmes.[23 24] Another study found an increase in number of grant awards for intervention participants but it is unclear if this difference was statistically significant.[44] Two out of three studies measuring the amount of funding (both amount of money and number of grants) received found significantly greater amounts of funding awarded to participants attending career development programmes with mentorship compared with non-attendees.[25 31]

Research participation

Research participation outcomes were reported in eight studies.[16 23 24 26 30 32 44 45] Four studies included a measure of involvement in research activities;[16 23 30 44] however, only two studies showed a significant increase in research participation – both studies assessed multi-faceted academic training programmes.[16 23] Mandel et al.,[32] found similar benefits for graduates taking part in a research-tailored curriculum intervention but it is unclear if this finding was significant. Two out of three measures relating to grant applications did not show any difference between groups in two interventions that included comprehensive research training.[26 45] Löwe et al.,[45] also found no benefit of a training programme on the number of participants writing a "book article" during residency.

Recruitment

Nine studies reported outcomes relating to recruitment in academia and achievement of a specific academic rank.[16 20 22 23 27 28 39 40 44] Two studies found that participants attending academic training programmes were significantly more likely to obtain their first job in academic practice post-training compared with non-attendees.[16 27] Three studies showed that intervention participants were significantly more likely to be recruited at higher academic ranks,[20] and achieve higher ranks of assistant professor[23] or full professor[28] more quickly than participants not enrolled in academic training programmes. Female faculty recruitment increased in two intervention studies aimed at improving diversity.[39 40] Valantine et al.,[40] found significant increases in recruitment at full professor level but no significant increase was evident at assistant or associate professor level. Similarly, Emans et al.,[22] found that female faculty recruitment for intervention participants only significantly increased at associate professor level opposed to non-significant increases for other academic levels.

Retention and promotion

Ten studies reported outcomes relating to retention, or promotion to a higher position.[18 19 21 22 25 29 30 35 37 38] The retention of staff in clinical academia was significantly higher for participants in two career development programmes[37 38] but non-significant in another career development programme,[25] and two academic training programmes.[21 30] In one study, residents of an academic training programme with research experience were significantly more likely to choose academia as a future career than participants in the control group.[30] One study found significantly higher retention rates for assistant professors who took part in a career development programme tailored for women;[18] whilst a similar programme aiming to improve diversity found no difference in retention of underrepresented minority staff.[19] One mentorship programme found significantly higher rates of promotion to associate professor in the intervention group,[35] whilst another study found no increase in promotions for participants in the intervention group.[25]

Publications

Publication outcomes were reported in 14 studies,[16 21 23-27 30 33 34 36 42 43 45] all of which measured the number of publications during or within five years of receiving an intervention. Nine studies found a significant increase in publication productivity, of which, eight were academic training programmes[16 21 23 27 30 33 43 45] and one was a mentorship programme.[34] The other five studies found either non-significant differences[24-26 42] or differences with no statistical analyses reported.[36] The number of first-author publications was reported in five studies;[21 24 27 43 45] but only three studies, all evaluating an academic training programme, showed a significant increase for intervention participants.[21 27 43] Publication impact was also measured across four studies, but only two studies demonstrated significantly higher H-index scores[21] or impact factors scores[27] for intervention participants taking part in an academic training programme. The number of peer review journal articles[24] and books published[45] were not found to be significantly different between intervention and control groups.

Synthesis of qualitative data

We identified seven themes:

Developing knowledge, skills and confidence in research and scholarship

Participants in seven studies reported improvements in research/scholarship knowledge and skills, and their confidence to conduct research activities, from planned teaching or learning sessions.[17 46 49 53 55 57 59] Respondents across three studies mentioned specific programme components as being useful for developing research/scholarship knowledge and skills.[46 53 59] The didactic sessions considered most helpful in one study focused on literature reviews, survey methodology and reference management.[53] Participants in another programme reported an unmet need for skills development including negotiation, grant management, and work-life balance, alongside a need for advice about a lack of faculty diversity and unconscious bias.[25]

Leadership skills and opportunities

Four studies reported positive findings about the leadership skills or opportunities that participants gained.[17 25 49 52] Respondents in one study gained leadership experience with a women-focused national organisation at an earlier career stage than anticipated, and this helped some earn promotion.[52] Individuals in two studies pursued new leadership opportunities, or progressed their careers in other ways, after gaining self-confidence in career development programmes.[17 49]

Personal characteristics and behaviour of individuals

Personal characteristics, including participants' level of personal ambition, enthusiasm, motivation, self-direction, interest, and commitment to the programme, influenced the decision to initially apply to a programme and/or participants' programme experiences in six studies.[47 49 54 58 61 62] Some participants in the UK Academy of Medical Sciences mentoring scheme reported less successful relationships with mentors due, at least in part, to mentees not having been sufficiently proactive in arranging meetings and maintaining contact with mentors.[62] In the UK Academic Foundation Programme, good preparation by trainees facilitated success, including arranging early contact with supervisors and maintaining engagement at key stages of

For peer review only

training. Conversely, one supervisor thought a barrier to success was that some candidates did not appreciate the time and work involved.[61]

Interactions and relationships

Networking through participation in career development initiatives, especially with individuals from other institutions, was valued for the benefits to career advancement and opportunities to identify mentors, and develop relationships with peers.[25 49 52] One study indicated that sponsorship was of benefit to women in terms of career advancement, including nominations for promotion and/or writing supportive references.[52]

Intervention participants gained emotional support, encouragement, self-confidence, and other benefits from relationships with peers in 11 studies.[17 25 46 47 49 52-55 59 61] Interacting and engaging with peers reduced feelings of professional isolation[49 52 54 59] and fostered a sense of community and belonging.[17 52 59] Women valued peer interaction, and the opportunity to share experiences, with other women.[47 49 52] Respondents also appreciated the professional collaboration that arose from peer relationships.[17 46 47 49 52 55 61] Some findings related to interactions explicitly described as peer mentoring.[17 25 47 53 59] Notably, we identified multiple terms used to describe peer interaction which made it difficult to draw clear distinctions between such terms and how they represent different forms of peer support.

Participants spoke positively about mentorship from senior colleagues.[25 46-49 52-55 57 58 62] In addition to being a source of moral support, self-confidence and encouragement, mentors provided other assistance, including offering career-related advice; teaching research and scholarship skills; facilitating leadership opportunities; assisting with grant applications; suggesting ways of dealing with rejection and setbacks; and providing resources such as staff or equipment.[25 46-48 55 62]

> *"When the challenges of combining clinical training and research clouded my judgment about future career steps, my mentor proved to be indispensable in making the most objective and adequate choice"*.[62]

Respondents believed it was important to develop a 'network' or team of several mentors, drawing on the different strengths and areas of expertise of each mentor, and guarding against inadequate mentoring.[46 47 52] Having at least one female mentor was important to some women in four studies, particularly in terms of having a role model, and providing guidance on balancing a career and family life.[47-49 52] One study found mixed views on whether both mentor and mentee need to be from an ethnic group underrepresented in medicine.[25] A respondent in another study believed some individuals may experience difficulties in finding mentors of the same ethnicity.[47] Mentees in two studies believed they gained more objective and impartial advice from having mentors who were from a different institution.[47 62] Trainees in one study preferred physicians over non-physicians as mentors, viewing them as role models.[58]

Studies reported mixed findings on the benefits of formalised mentorship programmes,[58 62] and on the need for training of mentors and mentees.[25 46 61 62]

Time and competing demands in clinical academia

Issues related to the time pressures experienced by clinical academics formed a consistent narrative across studies. Protected time was an important feature of career development programmes and training fellowships in seven studies.[17 46 50 53 55 56 58]

> *"Really what I needed was dedicated time so I'd have relief time from clinic to work on the project…it gave me a chance to… really move the project forward a lot more than I would have without it"*.[53]

One funding award enabled participants with substantial care-giving demands to gain greater control, flexibility, and choice over their time through buying more protected research time and hiring staff to take over various research-related tasks.[50] Awardees reported greater research productivity and an improved work-life balance. The award also assisted with career progression and retention in academia at critical time points. In another study, a participant found protected time facilitated achieving more publications.[46]

For peer review only

Some participants found it difficult in practice to maintain dedicated research time due to lack of clinical cover, and found that administrative staff were not always supportive which made scheduling protected time difficult.[50 53] Time conflicts, particularly competing clinical demands, acted as a barrier to organising or participating in specific programme elements, research training or implementing training fellowships across four studies.[50 53 57 58] The impact of the Athena SWAN programme was potentially undermined by institutions holding meetings at times that could be difficult for staff with caring responsibilities to attend.[60]

The competing commitments of clinical supervisors were barriers to success in the UK Academic Foundation Programme,[61] whilst in Canada, several Clinician Investigator Program programme directors did not have protected time for managing the programme.[56] Similarly, competing demands on senior faculty staff detracted from their ability to be a good mentor in another study.[47]

Facilitating programme participation and success

The influence of programme and organisational level factors on intervention participation and impact was identified across studies.

Having committed and experienced programme staff facilitated success in six studies.[17 46 53 55 56 58] We interpreted 'programme staff' as individuals involved in programme delivery, including teachers, administrative staff, and programme leads.

Management support influenced programme participation in four studies.[17 49 56 60] In one study, the provision of financial support to women faculty had enabled them to attend a career development programme.[49] In contrast, postdoctoral researchers perceived a lack of support from project leads and expressed scepticism that they would be given time to participate in Athena SWAN activities.[60]

In one study, most respondents supported the Athena SWAN programme and believed it had positive institutional outcomes.[60] However, the application process increased the workload of the mostly female self-assessment team, reinforcing institutional gender inequity and undermining the programme's aims.[60] A perceived emphasis on only supporting women was considered to perpetuate existing gendered social norms related to childcare provision.[60]

Two studies identified issues related to the promotion of programmes to staff.[49 60] Participants had not accessed initiatives, as they were unaware of them or had ignored relevant information to prevent 'email overload'.[60] Elsewhere, a lack of institutional information promoting programmes meant faculty only learned about them from colleagues.[49]

Seven studies identified factors influencing success related to the delivery of learning sessions and training fellowships.[17 46 56-59 61] Involvement in planning and developing sessions was considered important.[59] Various teaching methods were influential including: diverse educational methods; mixed guided and independent learning approaches; experiential learning; tailored coursework; and utilising the expertise of staff from different institutions.[17 46 57 58] Other beneficial aspects of programmes included: a 'supportive' learning environment, high degree of autonomy in research training, flexibility, structure, and clear guidance.[56 57] Barriers included some didactic content being judged as too jargonistic and delivered using inaccessible language.[17] Supervisors in the UK Academic Foundation Programme considered the short period of academic time a barrier.[61] Challenges related to infrastructure and logistics, for example the lack of suitable desk space, were found in two programmes.[54 58]

At a national level, UK funding arrangements could undermine family-friendly policies implemented to support Athena SWAN, for example by not providing funding for maternity cover in grant awards.[60] Meanwhile, in Canada, inadequate funding for trainees was viewed as a barrier to Clinician Investigator Program entry.[56]

Funding and financial support

Funding for protected time and issues related to national policies are discussed earlier. Two studies suggested improvements in the intervention including funding administrative staff or research personnel[50 53] and one reported that lack of funding resulted in limited access to specialist statistical support.[61] Funded fellowship

For peer review only

programmes were reported to be more successful because they provided protected time for developing programme infrastructure.[58]

The need for bridge or seed funding was mentioned in some studies.[50] Funding requirements considering time worked rather than chronological time limits was also deemed important:

> *"[A]pplications [where] eligibility [is limited to] 3 years within starting your faculty position or 8 years within graduating [should] have this prorated…. so that the eligibility is based on time worked, not just a chronologic year, which may have a 3-month maternity leave… in it".*[50]

Mentorship could help address some of the financial challenges for junior clinical academics, through supporting grant application writing, helping with bridging funds, or providing research and administrative staff support.[48]

Discussion

Statement of principal findings

We identified few high quality, well-reported evaluations of interventions to improve recruitment or retention to clinical academia. Most studies were from North America and no controlled quantitative studies were from the UK. No studies included interventions for clinical academic dentists, and few included specific interventions for women or minority groups. No studies reported on outcomes related to patient benefit, or cost-effectiveness of interventions.

Most quantitative evidence derived from multi-faceted academic training programmes; such programmes may increase recruitment to academia among clinicians, but findings were less clear for retention or other outcomes related to participation in research and research funding. Qualitative studies reported benefits of supportive relationships for clinical academics, including peer and senior mentors. Formalised mentoring programmes were not universally considered useful. There was consistent evidence of the importance of having protected time, particularly to mitigate against the negative impact of competing clinical demands on research-related activity, though maintaining protected time could be difficult in practice. Across studies, committed and experienced programme staff were key facilitators of success.

Strengths and limitations of this review

This review used rigorous, systematic, and transparent methods conducted by a highly experienced research team. The broad focus provides insights for clinical academics, programme leads, and funders. We used machine learning methodology to facilitate very high-volume title and abstract screening to identify the most relevant records earlier, resulting in better resource management by reducing the number of records to be screened by 50%. Nonetheless, the high volume of potentially eligible records and limited time resource meant it was not possible to investigate all relevant primary studies in depth.

Limitations in the reporting of the existing literature made synthesis difficult. Many studies involved highly motivated participants compared with controls or participants who did not get accepted onto a programme as control groups. Such groups are not directly comparable leading to potentially biased results caused by baseline differences. Furthermore, it is unclear to what extent findings, which derived mostly from studies conducted in the USA, can be applied to other contexts. Multiple factors, including inter-country differences in organisational structures and practices, potentially limit the generalisability of findings. These challenges in extrapolating from literature limited by quality and geography hindered our ability to draw robust conclusions on the effectiveness of interventions designed to support clinical academic careers within the UK.

Our findings in relation to previous studies

This review includes a more substantial volume of literature than the previous review by Laver et al.[4] Our work confirms their findings that most studies are from the USA and that the quality of research in this field is generally poor. Compared with the previous systematic review, our review used a more comprehensive search strategy, and included interventions for all clinical academics, not just women. Our review had a broader focus

For peer review only

than solely interventions to promote gender equality, but also found most evidence for multi-faceted interventions and those with a mentoring component.

Implications of this review for clinical academia and policy makers

The key implication identified by this review is that multi-faceted interventions are most likely to be successful in promoting clinical academic careers, with components such as protected time, mentorship, and supportive staff. The contribution of each facet of the intervention was not always clear due to incomplete reporting, and thus future work may consider using methods such as realist evaluation to distinguish the role each component plays in a programme's ability to deliver key outcomes for clinical academics.

The lack of quantitative evidence of benefit should be carefully considered when planning future mentorship interventions, particularly the need for detailed evaluations. Whilst our qualitative synthesis showed mentoring of junior clinical academics by seniors was often considered beneficial, the quantitative data were less supportive of mentoring interventions, with some findings in favour, but others finding no evidence of benefit. Most evidence suggested mentorship improved funding received and supported programmes incorporating both peer and senior mentorship. Mentorship team composition may play a key role in the success of the relationship, including factors such as the number, gender, and location of mentors. Little evidence related to mentor ethnicity. Formalised mentorship schemes, including requiring specific mentor characteristics, were not clearly supported by the literature. Our findings may reflect the design of individual programmes, or the studies evaluating them, but the need for further evaluation of mentorship programmes is clear.

Some studies suggested personal attributes of individual participants, such as commitment, enthusiasm, and motivation, could be key influences on intervention success, thus placing responsibility for programme failures on the individuals involved, rather than institutional or cultural factors. The complex interplay of gender, ethnicity, parenthood, and other protected characteristics on these attributes has not been explored within the included literature. Indeed, there was a notable absence of intersectional focus within the qualitative literature overall, with just two studies highlighting the interactions between gender and ethnicity.[50 52] In the quantitative synthesis, few studies focused on interventions tailored towards women and/or underrepresented minority faculty, and none evaluated an intervention from an intersectional standpoint. Adopting an intersectionality perspective when developing and evaluating future strategies may address more effectively issues related to inequality within clinical academia.

Various factors at organisational or national levels had negative impacts on the success of interventions within this review. Such evidence indicates that the success of future initiatives will be limited unless action is taken to ensure that organisational practice and culture, as well as relevant national policies, support the recruitment, retention, and progression of clinical academics.

Implications of this review for research

From the identified evidence on interventions, it is clear that little benefit will be derived from conducting further small, single-centre cohort studies in this field. Future research should use more robust methods to evaluate the effectiveness of interventions over time, using a control group and outcomes relating to recruitment and retention for medical and dental academics. We recommend that research funders commit to establishing large scale national research infrastructure to facilitate this, spanning both clinical and academic environments.

Interventions evaluated through this infrastructure are most likely to be successful when embedded within comprehensive multi-faceted programmes, focused on developing relationships between clinical academics. Consideration should be given to evaluating support structures, including administration, personnel, and programme leadership, as well as the most effective timing of interventions along the clinical academic career path. Evaluations of structural and environmental factors should be prioritised over interventions targeting individual determinants.

For peer review only - http://bmjopen.bmj.com/site/about/guidelines.xhtml

Clear terminology for describing clinical academics, and co-produced core outcome sets for studies of clinical academic careers, would aid the synthesis of primary studies in future systematic reviews. To allow for a focus on equality and diversity, results should be open-access and transparently presented in disaggregated form, reporting gender and ethnicity differences. In addition, the intersectional perspective is notably absent from this field and further high-quality, reflexive, qualitative research should explore the interplay of multiple determinants of inequality.

Within this review, we identified an additional dataset of studies evaluating barriers and facilitators to clinical academic careers. Further exploration and synthesis of this dataset may facilitate a deeper understanding of the barriers and facilitators that clinical academics face and may inform the development of specifically targeted interventions. This systematic review formed part of a larger project focused on UK clinical academic careers, for which linked primary qualitative research was performed. A full and detailed report of the whole project is available online.[63 64]

For peer review only

Statements

Ethics statement

This study did not receive nor require ethics approval, as it is a systematic review of existing literature.

Funding statement

This systematic review, as well as the larger project it is part of, was funded jointly by the Medical Research Council (MRC), the National Institute for Health Research (NIHR), Wellcome, Health Education England (HEE), the Academy of Medical Sciences, and Cancer Research UK (CRUK) through an award administered by CRUK [C71037/A29824]. The funders identified the focus of the work and the use of systematic review methodology to address the research question. The protocol for and findings of the study were not further influenced by the funders.

Competing Interest Statement

All authors have completed the ICMJE uniform disclosure form (available on request from the corresponding author) and declare: no support from any organisation for the submitted work beyond that described in our funding statement, no financial relationships with any organisations that might have an interest in the submitted work in the previous three years, no other relationships or activities that could appear to have influenced the submitted work.

Data sharing statement

Data included within this review have been extracted from the included studies, in their published form. No additional data is available.

Contributorship statement

JEM, GF, PC and LAS designed the study in collaboration and obtained funding as detailed. Search strategies were developed, tested, and translated by an information specialist with input from JVEB and JEM. JVEB and JEM drafted the protocol and then revised it alongside the other authors. JVEB, JEM, CE, GR, EU, PC, AK and GF screened titles and abstracts for inclusion. JEM, JVEB, CE, GR, and EU screened full texts, performed data extraction, and quality assessment. JEM, CE, GR and EU performed the analyses. All authors were involved in the writing and editing of the manuscript. All authors have read and approved the final manuscript.

Figure legends:

Figure 1 : Flowsheet for study selection

References

1. Fitzpatrick S. A survey of staffing levels of medical clinical academics in UK medical schools as at 31 July 2011. London; 2012.
2. Penny M, Jeffries R, Grant J, Davies SC. Women and academic medicine: a review of the evidence on female representation. Journal of the Royal Society of Medicine. 2014;107(7):259-63.
3. Watson N, Tang P, Knight E. Survey of medical clinical academic staffing levels. London; 2018.

For peer review only

4. Laver KE, Prichard IJ, Cations M, Osenk I, Govin K, Coveney JD. A systematic review of interventions to support the careers of women in academic medicine and other disciplines. BMJ Open. 2018;8(3):e020380.

5. Brown JVE, Crampton PES, Finn GM, Morgan JE, on behalf of the project team. From the sticky floor to the glass ceiling and everything in between: protocol for a systematic review of barriers and facilitators to clinical academic careers and interventions to address these, with a focus on gender inequality. Systematic Reviews. 2020;9(1):26.

6. World Bank. World Bank country and lending groups. World Bank Group; 2021 [12th April 2021]; Available from: https://datahelpdesk.worldbank.org/knowledgebase/articles/906519-world-bank-country-and-lending-groups.

7. Ouzzani M, Hammady H, Fedorowicz Z, Elmagarmid A. Rayyan—a web and mobile app for systematic reviews. Systematic Reviews. 2016;5(1):210.

8. Brown J, Stewart L, Finn G, Morgan J. Optimising machine learning to support very high-volume title and abstract screening for systematic reviews. ; 2021. In submission.

9. Higgins JPT, Altman DG, Gøtzsche PC, Jüni P, Moher D, Oxman AD, et al. The Cochrane Collaboration's tool for assessing risk of bias in randomised trials. BMJ. 2011;343:d5928.

10. Wells G, Shea B, O'Connell D, Peterson J, Welch, Losos M, et al. The Newcastle-Ottawa Scale (NOS) for assessing the quality of nonrandomised studies in meta-analyses. 2014 [12th April 2021]; Available from: http://www.ohri.ca/programs/clinical_epidemiology/oxford.asp.

11. Joanna Briggs Institute. Joanna Briggs Institute reviewers' manual: 2014 edition. Australia: The Joanna Briggs Institute; 2014.

12. Hong QN, Fàbregues S, Bartlett G, Boardman F, Cargo M, Dagenais P, et al. The mixed methods appraisal tool (MMAT) version 2018 for information professionals and researchers. Education for Information. 2018;34:285-91.

For peer review only

13. Wong G, Westhorp G, Greenhalgh J, Manzano A, Jagosh J, Greenhalgh T. Quality and reporting standards, resources, training materials and information for realist evaluation: the RAMESES II project. Health Services and Delivery Research. 2017;5.

14. Braun V, Clarke V. Using thematic analysis in psychology. Qualitative Research in Psychology. 2006;3(2):77-101.

15. Healthwatch York. Healthwatch York Homepage. York: Healthwatch York; [12th April 2021]; Available from: https://www.healthwatchyork.co.uk/.

16. Brandt AM, Rettig SA, Kale NK, Zuckerman JD, Egol KA. Can a Clinician-Scientist Training Program Develop Academic Orthopaedic Surgeons? One Program's Thirty-Year Experience. Journal of Surgical Education. 2018 Jul - Aug;75(4):1039-44.

17. Campion MW, Bhasin RM, Beaudette DJ, Shann MH, Benjamin EJ. Mid-career faculty development in academic medicine: How does it impact faculty and institutional vitality? The Journal of Faculty Development. 2016 Sep;30(3):49-64.

18. Chang S, Morahan PS, Magrane D, Helitzer D, Lee HY, Newbill S, et al. Retaining Faculty in Academic Medicine: The Impact of Career Development Programs for Women. Journal of Women's Health. 2016 Jul;25(7):687-96.

19. Daley S, Wingard DL, Reznik V. Improving the retention of underrepresented minority faculty in academic medicine. Journal of the National Medical Association. 2006 Sep;98(9):1435-40.

20. Dannels SA, Yamagata H, McDade SA, Chuang YC, Gleason KA, McLaughlin JM, et al. Evaluating a leadership program: a comparative, longitudinal study to assess the impact of the Executive Leadership in Academic Medicine (ELAM) Program for Women. Academic Medicine. 2008 May;83(5):488-95.

21. Ehlers SL, Cornelius KE, Greenberg-Worisek AJ, Warner DO, Weavers KM, Thomson KR, et al. A matched cohort examination of publication rates

For peer review only

among clinical subspecialty fellows enrolled in a translational science training program. Journal Of Clinical And Translational Science. 2018 Oct;2(5):327-33.

22. Emans SJ, Goldberg CT, Milstein ME, Dobriner J. Creating a faculty development office in an academic pediatric hospital: challenges and successes. Pediatrics. 2008 Feb;121(2):390-401.

23. Goldenberg NA, Kruse-Jarres R, Frick N, Pipe SW, Leissinger CA, Kessler CM. Outcomes of mentored, grant-funded fellowship training in haemostasis /thrombosis: findings from a nested case-control survey study. Haemophilia. 2012 May;18(3):326-31.

24. Grisso JA, Sammel MD, Rubenstein AH, Speck RM, Conant EF, Scott P, et al. A Randomized Controlled Trial to Improve the Success of Women Assistant Professors. Journal of Women's Health. 2017 May;26(5):571-9.

25. Guevara JP, Wright M, Fishman NW, Krol DM, Johnson J. The Harold Amos Medical Faculty Development Program: Evaluation of a National Program to Promote Faculty Diversity and Health Equity. Health Equity. 2018 2018;2(1):7-14.

26. Harrison LM, Woods RJ, McCarthy MC, Parikh PP. Development and implementation of a sustainable research curriculum for general surgery residents: A foundation for developing a research culture. American Journal of Surgery. 2020 Jul;220(1):105-8.

27. Joshua Smith J, Patel RK, Chen X, Tarpley MJ, Terhune KP. Does intentional support of degree programs in general surgery residency affect research productivity or pursuit of academic surgery? Journal of Surgical Education. 2014 Jul-Aug;71(4):486-91.

28. Khot S, Park BS, Longstreth WT, Jr. The Vietnam War and medical research: untold legacy of the U.S. Doctor Draft and the NIH "Yellow Berets". Academic Medicine. 2011 Apr;86(4):502-8.

29. Kohlwes RJ, Shunk RL, Avins A, Garber J, Bent S, Shlipak MG. The PRIME curriculum. Clinical research training during residency. Journal of General Internal Medicine. 2006 May;21(5):506-9.

For peer review only

30. Kohlwes J, O'Brien B, Stanley M, Grant R, Shunk R, Connor D, et al. Does Research Training During Residency Promote Scholarship and Influence Career Choice? A Cross-Sectional Analysis of a 10-Year Cohort of the UCSF-PRIME Internal Medicine Residency Program. Teaching & Learning in Medicine. 2016 Jul-Sep;28(3):314-9.

31. Libby AM, Hosokawa PW, Fairclough DL, Prochazka AV, Jones PJ, Ginde AA. Grant Success for Early-Career Faculty in Patient-Oriented Research: Difference-in-Differences Evaluation of an Interdisciplinary Mentored Research Training Program. Academic Medicine. 2016 Dec;91(12):1666-75.

32. Mandel BA, Weber SM, Gutowski KA, Salyapongse AN, Bentz ML. What Influences a Plastic Surgery Resident to Pursue an Academic Career? Plastic and Reconstructive Surgery - Global Open. 2018 Oct;6(10):e1860.

33. Mills LS, Steiner AZ, Rodman AM, Donnell CL, Steiner MJ. Trainee participation in an annual research day is associated with future publications. Teaching & Learning in Medicine. 2011 Jan;23(1):62-7.

34. Nasab S, Rushing JS, Segars JH, Evers E, Handa VL, Lawson S, et al. A Mentorship Program for Academic Obstetrician Gynecologists that Improved Publication and Overall Confidence for Success. Seminars in Reproductive Medicine. 2019 Sep;37(5-06):257-64.

35. Ockene JK, Milner RJ, Thorndyke LE, Congdon J, Cain JM. Peers for Promotion: Achieving Academic Advancement through Facilitated Peer Mentoring. Journal of Faculty Development. 2017 2017;31(3):5-13.

36. Patel MS, Tomich D, Kent TS, Chaikof EL, Rodrigue JR. A Program for Promoting Clinical Scholarship in General Surgery. Journal of Surgical Education. 2018 Jul - Aug;75(4):854-60.

37. Ries A, Wingard D, Morgan C, Farrell E, Letter S, Reznik V. Retention of junior faculty in academic medicine at the University of California, San Diego. Academic Medicine. 2009 Jan;84(1):37-41.

For peer review only

38. Ries A, Wingard D, Gamst A, Larsen C, Farrell E, Reznik V. Measuring faculty retention and success in academic medicine. Academic Medicine. 2012 Aug;87(8):1046-51.
39. Sheridan JT, Fine E, Pribbenow CM, Handelsman J, Carnes M. Searching for excellence & diversity: increasing the hiring of women faculty at one academic medical center. Academic Medicine. 2010 Jun;85(6):999-1007.
40. Valantine HA, Grewal D, Ku MC, Moseley J, Shih MC, Stevenson D, et al. The gender gap in academic medicine: comparing results from a multifaceted intervention for stanford faculty to peer and national cohorts. Academic Medicine. 2014 Jun;89(6):904-11.
41. Winn AS, Emans SJ, Newman LR, Sandora TJ. Promoting Resident Professional Development Using Scholarly Academies. Academic Pediatrics. 2018 May - Jun;18(4):477-9.
42. Klimas J, Fernandes E, deBeck K, Hayashi K, Milloy MJ, Kerr T, et al. Preliminary Results and Publication Impact of a Dedicated Addiction Clinician Scientist Research Fellowship. Journal of Addiction Medicine. 2017 Jan/Feb;11(1):80-1.
43. Merani S, Switzer N, Kayssi A, Blitz M, Ahmed N, Shapiro AM. Research productivity of residents and surgeons with formal research training. Journal of Surgical Education. 2014 Nov-Dec;71(6):865-70.
44. Sweeny A, van den Berg L, Hocking J, Renaud J, Young S, Henshaw R, et al. A Queensland research support network in emergency healthcare. Journal of Health Organization & Management. 2019 Mar 18;33(1):93-109.
45. [illegible], Hartmann M, Wild B, Nikendei C, Kroenke K, Niehoff D, et al. Effectiveness of a 1-year resident training program in clinical research: a controlled before-and-after study. Journal of General Internal Medicine. 2008 Feb;23(2):122-8.
46. Comeau DL, Escoffery C, Freedman A, Ziegler TR, Blumberg HM. Improving clinical and translational research training: a qualitative evaluation of the Atlanta Clinical and Translational Science Institute KL2-mentored

For peer review only

research scholars program. Journal of Investigative Medicine. 2017 Jan;65(1):23-31.

47. DeCastro R, Sambuco D, Ubel PA, Stewart A, Jagsi R. Mentor networks in academic medicine: moving beyond a dyadic conception of mentoring for junior faculty researchers. Academic Medicine. 2013a Apr;88(4):488-96.

48. DeCastro R, Sambuco D, Ubel PA, Stewart A, Jagsi R. Batting 300 is good: perspectives of faculty researchers and their mentors on rejection, resilience, and persistence in academic medical careers. Academic Medicine. 2013b Apr;88(4):497-504.

49. Helitzer DL, Newbill SL, Cardinali G, Morahan PS, Chang S, Magrane D. Narratives of Participants in National Career Development Programs for Women in Academic Medicine: Identifying the Opportunities for Strategic Investment. Journal of Women's Health. 2016 Apr;25(4):360-70.

50. Jones RD, Miller J, Vitous CA, Krenz C, Brady KT, Brown AJ, et al. The Most Valuable Resource Is Time: Insights from a Novel National Program to Improve Retention of Physician-Scientists with Caregiving Responsibilities. Academic Medicine. 2019 Nov;94(11):1746-56.

51. Kraemer RR, Wakelee JF, Hites L, Frank SJ, Saag K, Rogers DA, et al. Moving Career Development Upstream: Evaluation of a Course for Internal Medicine Trainees Contemplating Career Pathways in Academic Medicine. Southern Medical Journal. 2018 Aug;111(8):471-5.

52. Lin MP, Lall MD, Samuels-Kalow M, Das D, Linden JA, Perman S, et al. Impact of a Women-focused Professional Organization on Academic Retention and Advancement: Perceptions From a Qualitative Study. Academic Emergency Medicine. 2019 Mar;26(3):303-16.

53. Reader S, Fornari A, Simon S, Townsend J. Promoting Faculty Scholarship - An evaluation of a program for busy clinician-educators. Canadian Medical Education Journal. 2015;6(1):e43-60.

For peer review only

54. Stubbe D, Martin A, Bloch M, Belitsky R, Carter D, Ebert M, et al. Model curriculum for academic child and adolescent psychiatry training. Academic Psychiatry. 2008 Sep-Oct;32(5):366-76.
55. Archibald D, Hogg W, Lemelin J, Dahrouge S, St Jean M, Boucher F. Building capacity for medical education research in family medicine: the Program for Innovation in Medical Education (PIME). Health Research Policy & Systems. 2017 Oct 23;15(1):91.
56. Hayward CP, Danoff D, Kennedy M, Lee AC, Brzezina S, Bond U. Clinician investigator training in Canada: a review. Clinical & Investigative Medicine. 2011 Aug 1;34(4):E192-E201.
57. Klimas J, McNeil R, Ahamad K, Mead A, Rieb L, Cullen W, et al. Two birds with one stone: experiences of combining clinical and research training in addiction medicine. BMC Medical Education. 2017a Jan 23;17(1):22.
58. Klimas J, Small W, Ahamad K, Cullen W, Mead A, Rieb L, et al. Barriers and facilitators to implementing addiction medicine fellowships: a qualitative study with fellows, medical students, residents and preceptors. Addiction Science & Clinical Practice. 2017b Sep 20;12(1):21.
59. Moss J, Teshima J, Leszcz M. Peer group mentoring of junior faculty. Academic Psychiatry. 2008 May-Jun;32(3):230-5.
60. Caffrey L, Wyatt D, Fudge N, Mattingley H, Williamson C, McKevitt C. Gender equity programmes in academic medicine: a realist evaluation approach to Athena SWAN processes. BMJ Open. 2016 Sep 8;6(9):e012090.
61. Darbyshire D, Baker P, Agius S, McAleer S. Trainee and supervisor experience of the Academic Foundation Programme. Journal of the Royal College of Physicians of Edinburgh. 2019 Mar;49(1):43-51.
62. Iversen AC, Eady NA, Wessely SC. The role of mentoring in academic career progression: a cross-sectional survey of the Academy of Medical Sciences mentoring scheme. Journal of the Royal Society of Medicine. 2014 Aug;107(8):308-17.

For peer review only

63. Finn G, Brown J, Evans C, Raine G, Stewart L, Uphoff E, et al. From the sticky floor to the glass ceiling and everything in between: A systematic review and qualitative study focusing on gender inequalities in Clinical Academic careers. Final Report. Commissioned by: NIHR Academy, Academy of Medical Sciences, Cancer Research UK, Health Education England, Medical Research Council, and Wellcome Trust. 2020; Available from: https://www.hyms.ac.uk/research/research-centres-and-groups/hpeu/gender-inequalities-in-clinical-academic-careers.

64. Finn G, Brown J, Evans C, Raine G, Stewart L, Uphoff E, et al. Inequalities in UK clinical academic careers: a systematic review and qualitative study. Short Report. Commissioned by: NIHR Academy, Academy of Medical Sciences, Cancer Research UK, Health Education England, Medical Research Council, and Wellcome Trust. 2021; Available from: https://www.hyms.ac.uk/research/research-centres-and-groups/hpeu/gender-inequalities-in-clinical-academic-careers.

For peer review only

For peer review only

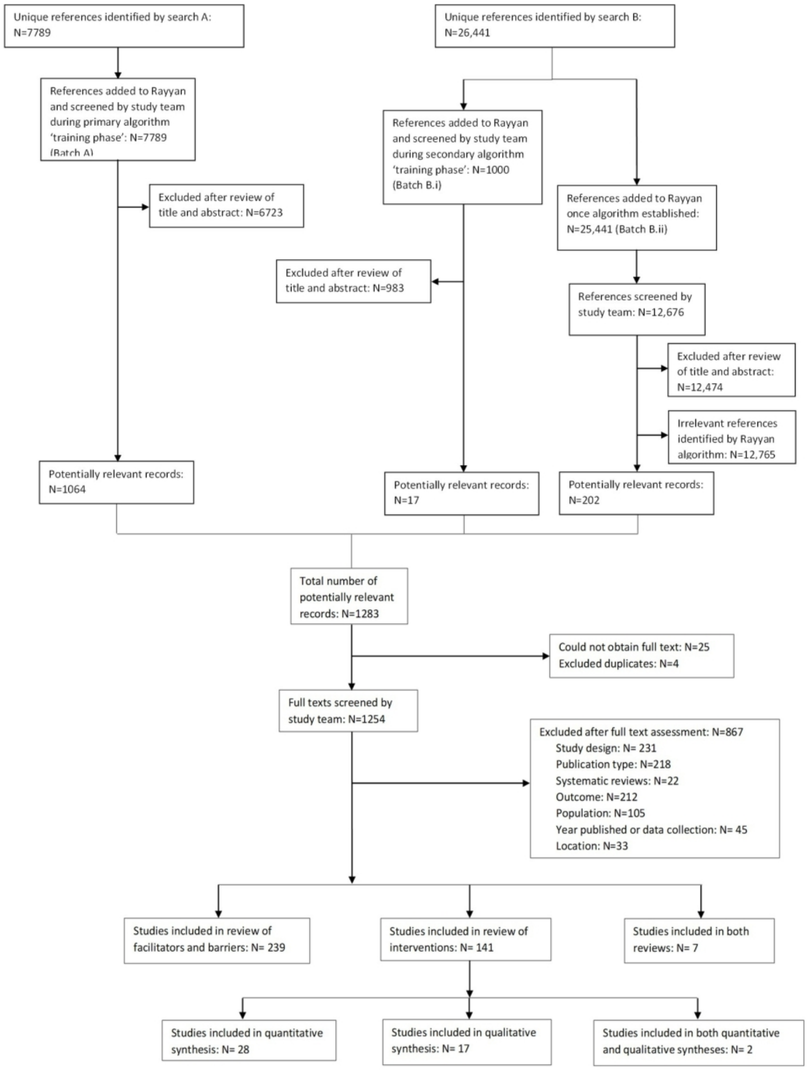

Flowsheet for study selection

153x194mm (300 x 300 DPI)

Appendix 1: Table of data for studies included in synthesis

First author (Year of publication; country)	Years of data collection	Study design	Grade/type of clinical academics included	Number of participants (Male: Female)	Ethnicity	Intervention components	Target population of intervention	Aimed at particular group	Reach
Studies included within quantitative analysis									
Brandt (2018; USA)[16]	NR- NR	Cohort study. Concurrent & historical control	Orthopaedic surgery residents, academic level NR	329 (NR)	NR	Mentorship programme - junior mentee/senior mentor, Protected research time	Trainees	No	Single centre
Chang (2016; USA)[18]	1988-2009	Cohort study. Concurrent control	Clinically mixed group, post-training, range of academic levels	65099, of which 3268 in intervention group	NR	Leadership training. Faculty career development program, not described in detail for individual programs. EWIM & MWIM programs focus on academic skills development; ELAM is leadership training	Junior Faculty, Managers/ leaders/ senior faculty	Gender	National
Daley (2006; USA)[19]	NR- 2005	Cohort study. Concurrent & historical control	Mixed group of faculty (medical, surgical, psychiatry, GP), mixed academic levels	112 (48:64)	15 URM	Mentorship programme - junior mentee/ senior mentor. Teaching sessions/ lectures, leadership training, faculty career development programme, networking/ collaboration/ social activities, academic performance counselling	Junior Faculty	Ethnicity, URM	Single centre
Dannels (2008; USA)[20]	2002-2006	Cohort study. Concurrent control	Post-training, post-doctoral. Speciality NR	250 (0:250)	NR	Leadership training	Managers/ leaders/ senior faculty	Gender	Single centre
Ehlers (2018; USA)[21]	2018-2018	Cohort study. Concurrent control	Pre-doctoral fellows in cardiovascular diseases, gastroenterology and hepatology/ oncology	140 (76:64)	NR	Mentorship programme - junior mentee/ senior mentor. Teaching sessions/ lectures, protected research time. Teaching is focused on research training. E.g. research methods, protocol development, grant writing and manuscript writing, statistics and epidemiology. Also complete a mentored research project	Trainees, Junior Faculty	No	Single centre
Emans (2008; USA)[22]	2006- NR	Cohort study. Historical control	Academic paediatric faculty. Academic level NR	238 (54%:46%)	NR	Mentorship programme - junior mentee/ senior mentor. Teaching sessions/ lectures, leadership training, networking/ collaboration/ social activities, 'Community	Junior Faculty	Gender, Ethnicity	Single centre

For peer review only

First author (Year of publication; country)	Years of data collection	Study design	Grade/type of clinical academics included	Number of participants (Male: Female)	Ethnicity	Intervention components	Target population of intervention	Aimed at particular group	Reach
						of mentors'. Work-life balance, cultural competency and diversity initiatives			
Goldenberg (2012; USA)[23]	NR- NR	Case control. Concurrent control	Trainees in academic haematology/ oncology. Academic level NR	20 (10 females)	NR	Mentorship programme - junior mentee/ senior mentor	Trainees	No	National
Grisso (2017; USA)[24]	2010- NR	Multifaceted cluster randomised intervention trial	Assistant professors, speciality NR	132 (0:132)	7.6% African American, 60.3% White, 27.5% Asian, 4.6% Hispanic/ other	Mentorship programme - junior mentee/ senior mentor. Mentorship programme - peers, grant writing programme, teaching sessions/ lectures, networking/ collaboration/ social activities, manuscript writing programme, also included a "Total Leadership Programme" which address work-life integration/ balance	Junior Faculty	Gender	Single centre
Harrison (2020; USA)[26]	2007-2017	Cohort study. Historical control	General surgery residents, academic level NR	203 (NR)	NR	Mentorship programme - junior mentee/ senior mentor. Teaching sessions/ lectures, annual milestones, research meetings and feedback on completing research projects	Trainees	No	Single centre
Joshua Smith (2014; USA)[27]	NR-NR	Cohort study. Concurrent control	General surgery trainees. Academic level NR	68 (NR)	NR	Research training; Protected research time	Trainees	No	Single centre
Khot (2011; USA)[28]	2007-2007	Cohort study. Concurrent control	Post-training, post-doctoral. Speciality not reported	1577 NIH associates (4 female) who entered academic medicine; 27821 non-associates	NR	Teaching sessions/ lectures, protected research time. Little detail reported	Trainees	No	National
Klimas (2017; Canada)[42]	2014-2014	Cohort study. Concurrent control	Trainees in internal medicine, family medicine, public health,	8 (3:5)	NR	Mentorship programme - junior mentee/ senior mentor. Teaching sessions/ lectures	Trainees	No	National

For peer review only

First author (Year of publication; country)	Years of data collection	Study design	Grade/type of clinical academics included	Number of participants (Male: Female)	Ethnicity	Intervention components	Target population of intervention	Aimed at particular group	Reach
			psychiatry, mix of academic levels						
Kohlwes (2006; USA)[29]	2001-2004	Cohort study. Concurrent control	Trainees in internal medicine. Academic level NR	32 (NR)	NR	Mentorship programme - junior mentee/senior mentor. Teaching sessions/ lectures, protected research time, small group journal clubs; work in progress sessions; conduct a clinical research project	Trainees	No	Single Centre
Kohlwes 2016; USA)[30]	NR- NR	Cohort study. Concurrent control	Post-training in internal medicine. Academic level NR	169 (88:81)	88 white, 1 black, 31 Asian Indian, 28 Asian Other, 12 Hispanic, 6 Other	Mentorship programme - junior mentee/ senior mentor. Teaching sessions/ lectures, protected research time, participants conduct research projects. Salary support provided for PRIME director and associate director	Trainees	No	Single Centre
Libby (2016; USA)[31]	2000-2011	Cohort study. Concurrent control	Faculty from many disciplines of medicine/surgery as well as disciplines outside medicine. Post-doctoral	25 CFSP scholars; 125 comparison faculty (NR)	NR	Mentorship programme - junior mentee/ senior mentor. Mentorship programme - peers, teaching sessions/ lectures, protected research time	Junior Faculty	No	Single centre
Löwe (2008; Western Europe)[45]	2005-2006	Cohort study. Concurrent control	Trainees from Internal Medicine, psychotherapy, psychosomatics, psychiatry, psychology. Academic level NR	44 (17:27)	NR	Mentorship programme - junior mentee/ senior mentor. Teaching sessions/ lectures, teaching sessions held as part of a 'clinical research methods' course. Participants also conducted an individual research project	Trainees	No	Single centre
Mandel (2018; USA)[32]	NR- NR	Cohort study. Historical control	Post-training plastic surgeons, academic level NR	28 (24:4)	NR	Teaching sessions/lectures, protected research time. 1-2 year research fellowship, senior mentorship	Trainees	No	Single centre
Merani (2014; Canada)[43]	1988-2012	Cohort study. Concurrent control	General surgery residents, mixed pre-doctoral and doctoral	323 (NR)	NR	Research training	Trainees	No	National

First author (Year of publication; country)	Years of data collection	Study design	Grade/type of clinical academics included	Number of participants (Male: Female)	Ethnicity	Intervention components	Target population of intervention	Aimed at particular group	Reach
Mills (2011; USA)[33]	Post 2007-NR	Cohort study. Concurrent control	Paediatric residents and fellows, academic level NR	526 (197:297)	NR	Departmental research day at which paediatrics trainees present submitted research and other scholarly work	Trainees	No	Single centre
Nasab (2019; USA)[34]	2018-2018	Cohort study. Concurrent control	Obstetricians & Gynecologists, trainees and post-training, range of academic levels	109 attended, 94 pre-course survey, 74 post course survey (NR)	63% white, 9% black, 5% Asian, 3% other, 20% did not disclose	Mentorship programme - junior mentee/ senior mentor. Teaching sessions/lectures, leadership training, networking/ collaboration/ social activities. Particular sessions included mock interviews, CV reviews, and grant writing support. 60-minute interactive sessions. Participants also given the opportunity to submit their grant proposals	Trainees, Junior Faculty	No	Single Centre
Ockene (2017; USA)[35]	NR- NR	Cohort study. Concurrent control	Post-doctoral, post-training, primary care physicians	32 (11:21)	NR	Mentorship programme - junior mentee/ senior mentor. Mentorship programme – peers. Teaching sessions/ lectures, networking/ collaboration/ social activities	Junior Faculty	No	Single centre
Patel (2018; USA)[36]	2011-2016	Cohort study. Historical control	General surgery residents and faculty mentors. Mentors post-doctoral	67 (NR)	NR	Mentorship programme - junior mentee/ senior mentor. Teaching sessions/ lectures, protected research time, completion of a defined clinical research project. Other didactic activities included: active participation in the department's weekly clinical research meeting; attend lab meetings with faculty research mentor. Webinars on research and grant writing	Trainees	No	Single centre
Ries (2009; USA)[37]	1988-2006	Cohort study. Concurrent control	Assistant professors in school of medicine. Speciality NR	839 (520-319)	52 URM	Mentorship programme - junior mentee/ senior mentor. Networking/ collaboration/ social activities, professional development workshops, performance counselling sessions	Junior Faculty	No	Single centre
Ries (2012; USA)[38]	Post 2005-2006	Case control. Concurrent control	Assistant professors from mixed specialities	315 (152:163)	26 URM	Mentorship programme - junior mentee/ senior mentor. Networking/ collaboration/ social activities, faculty career development programme, Performance counselling sessions	Junior Faculty	No	Single centre

For peer review only

First author (Year of publication; country)	Years of data collection	Study design	Grade/type of clinical academics included	Number of participants (Male: Female)	Ethnicity	Intervention components	Target population of intervention	Aimed at particular group	Reach
Sheridan (2010; USA)[39]	2000-2008	Cohort study. Concurrent control	Post-training. Speciality NR. Academic level NR	163 (NR)	NR	Recruitment training (e.g. to reduce bias in interviews)	Junior Faculty, Managers/ leaders/ senior faculty	Gender, Ethnicity, "Individuals from any group that has been historically under-represented on the faculties of academic health centres"	Single centre
Sweeny (2019; Australia/New Zealand)[44]	2015-2018	Cohort study. Historical control	Emergency Medicine, grade and academic level NR	33 Emergency departments (NR)	NR	Networking/ collaboration/ social activities, research support	Clinical researchers in hospital departments	No	National
Valantine (2014; USA)[40]	2011-2011	Cohort study. Concurrent control	Post-training, post-doctoral, speciality NR	Varying number of participants over several time periods	NR	Mentorship programme - junior mentee/ senior mentor. Funding award, teaching sessions/ lectures, leadership training, protected research time, networking/ collaboration/ social activities, McCormick faculty awards. Faculty Fellows leadership program - individualised career development planning, skill building workshops, and the Women's faculty networking program. Intervention involved a series of programmes	Junior Faculty, Managers/ leaders/ senior faculty	Gender	Single centre
Winn (2018; USA)[41]	2013-2016	Cohort study, case control. Historical control	Internal medicine and paediatrics trainees, academic level NR	48 (NR)	NR	Mentorship programme - junior mentee/senior mentor. Mentorship programme – peers. Teaching sessions/lectures, protected research time, "scholarly homes"	Trainees	No	Single centre
Studies included within qualitative analysis									

For peer review only

First author (Year of publication; country)	Years of data collection	Study design	Grade/type of clinical academics included	Number of participants (Male: Female)	Ethnicity	Intervention components	Target population of intervention	Aimed at particular group	Reach
Archibald (2017; Canada)[55]	2013-2013	Qualitative	Physicians in family medicine, academic level NR	14 (NR)	NR	Mentorship programme - junior mentee/ senior mentor. Funding award, protected research time. Assistants and coordinators provide resources and research support, networking/ collaboration/ social activities	All faculty staff	No	Single centre
Caffrey (2016; UK)[60]	2015-2015	Qualitative	Medical school staff, grade NR, mixed academic levels	31 (NR)	NR	Athena SWAN programme	Trainees, Junior Faculty, Managers/ leaders/ senior faculty, Relevant to whole of department	Gender	National
Comeau (2017; USA)[46]	2007-2014	Mixed methods. Quantitative work had no control	Trainees and post-training, mix of specialities, mix of academic levels	46 (16:30)	22 White, 24 Minority	Mentorship programme - junior mentee/ senior mentor. Funding award, teaching sessions/ lectures, protected research time	Junior Faculty	No	National
Darbyshire (2019; UK)[61]	2014-2014	Mixed methods. Quantitative work had no control	AFP Trainees (survey) and post-training (interviews), mix of specialities, pre-doctoral	34 survey; 7 interviews (NR)	NR	Research training programme; protected research time	Trainees	No	National
*DeCastro (2013a; USA)[47]	2010-2011	Qualitative	Post-training, mix of specialities, mixed academic levels	128 (52:76)	99 White, 7 Black, 3 Hispanic, 18 Asian	Mentorship programme - junior mentee/senior mentor. Protected research time, funding award	Junior Faculty	No	National
*DeCastro (2013b; USA)[48]	2010-2011	Qualitative	Post-training, mix of specialities, mixed academic levels	128 (52:76)	99 White, 7 Black, 3 Hispanic, 18 Asian	Mentorship programme - junior mentee/senior mentor. Protected research time, funding award	Junior Faculty	No	National

For peer review only

First author (Year of publication; country)	Years of data collection	Study design	Grade/type of clinical academics included	Number of participants (Male: Female)	Ethnicity	Intervention components	Target population of intervention	Aimed at particular group	Reach
Hayward (2011; Canada)[56]	2008-2008	Mixed methods. Quantitative work had no control	Trainees and post-training, mix of specialities, mix of academic levels	211 (survey); 13 (qualitative) (NR)	NR	Teaching sessions/ lectures, protected research time, mentorship programme - junior mentees/ senior mentors	Trainees	No	National
Helitzer (2016; USA)[49]	2011- NR	Qualitative (Part of a larger mixed methods study but only qualitative findings reported in this paper)	Post-training, mix of specialities, mixed academic levels	45 (0:45)	NR	Not described in detail for the three programmes separately. EWIM & MWIM held over three days and involved skills and confidence building. ELAM programme was leadership training. All three are faculty career development programmes	Junior Faculty, Managers/ leaders/ senior faculty	Gender	National
Iversen (2014; UK)[62]	2010-2010	Mixed methods. Quantitative work had no control	Post-training, mix of specialities, academic level unclear	147 mentees (67% male) & 77 mentors (81% male)	NR	Mentorship programme - junior mentee/ senior mentor	Junior Faculty	No	National
Jones (2019; USA)[50]	2018-2018	Qualitative study	Early-career or junior physician-scientists, range of specialities, academic level NR	28 (5:23)	11 Non-Hispanic white; 7 Asian; 7 Other; 3 NR	Funding award. Some institutions have also provided access to other forms of support, such as leadership development training, training in academic and career development skills, and/or networking.	Junior Faculty	No, but 85% of awardees are women and tailored more towards those with extra-professional caregiving demands	National
*Klimas (2017a; Canada)[57]	2015-2015	Qualitative	Fellowship in addiction medicine. Trainees from Psychiatry,	26 (12:14)	NR	Mentorship programme - junior mentee/ senior mentor. Funding award, teaching sessions/ lectures, specialty training	Trainees	No	Single centre

For peer review only - http://bmjopen.bmj.com/site/about/guidelines.xhtml

First author (Year of publication; country)	Years of data collection	Study design	Grade/type of clinical academics included	Number of participants (Male: Female)	Ethnicity	Intervention components	Target population of intervention	Aimed at particular group	Reach
			Internal Medicine, Family Medicine, Nursing & Social Work. Academic level NR						
*Klimas (2017b; Canada)[58]	2015-2015	Qualitative	Fellowship in addiction medicine. Trainees from Psychiatry, Internal Medicine, Family Medicine, Nursing & Social Work. Academic level NR	26 (12:14)	NR	Mentorship programme - junior mentee/ senior mentor. Funding award, teaching sessions/ lectures, specialty training	Trainees	No	Single centre
Kraemer (2018; USA)[51]	2014-2016	Mixed methods. Quantitative work had no control	Residents and fellows in medical specialities. Academic level NR	69 (41 residents, 28 fellows)	NR	Teaching sessions/ lectures. Faculty career development programme	Trainees	No	Single centre
Lin (2019; USA)[52]	Post 2009- NR	Qualitative	Post-training, post-doctoral, emergency medicine	17 (0:17)	12 Caucasian; 4 Asian American; 1 African American.	Mentorship programme - junior mentee/ senior mentor. Networking/ collaboration/ social activities, skill enhancement, e.g. negotiating skills	Junior Faculty, Managers/ leaders/ senior faculty	Gender	National
Moss (2008; Canada)[59]	2005-2005	Qualitative	Lecturers and assistant professors in Psychiatry	10 (6:4)	NR	Mentorship programme - peers	Junior Faculty	No	Single centre
Reader (2015; USA)[53]	2009-2009	Mixed methods. Quantitative work had no control	Post-training, speciality NR, mix of academic levels	10 (2:8)	NR	Mentorship programme - junior mentee/ senior mentor. Mentorship programme – peer. Teaching sessions/ lectures, protected research time. Faculty career development programme	Junior Faculty, Managers/ leaders/ senior faculty	Designed for “busy clinician educators”	Single centre

For peer review only

First author (Year of publication; country)	Years of data collection	Study design	Grade/type of clinical academics included	Number of participants (Male: Female)	Ethnicity	Intervention components	Target population of intervention	Aimed at particular group	Reach
Stubbe (2008; USA)[54]	NR- NR	Qualitative	Academic Child and Adolescent Psychiatry Residents. Academic level NR	5 (NR)	NR	Mentorship programme - junior mentee/ senior mentor, Teaching sessions/ lectures, Protected research time	Trainees	No	Single centre
Studies included within both quantitative and qualitative analysis									
Campion (2016; USA)[17]	2014-2014	Mixed methods. Quantitative work had concurrent control	Post-doctoral, clinical faculty and school of public health faculty	16 in intervention group (>60% women) 25 people in reference group	30% URM	Mentorship programme – peers. Teaching sessions/ lectures, protected research time, networking/ collaboration/ social activities	Junior faculty	No	Single centre
Guevara (2018; USA)[25]	NR- NR	Mixed methods. Quantitative work had concurrent control	Medical residents, junior staff and assistant profs. Academic level unclear	124 (65:59)	91 African American, 29 Hispanic Latino, 4 Native American	Funding award	Trainees, Junior Faculty	Ethnicity, URM	National

* Both papers by DeCastro et al.[47 48] and Klimas et al.[57 58] were based on data collected from a single sample of respondents.

Abbreviations: AFP – Academic Foundation Programme; CFSP – Clinical Faculty Scholars Program; CV – Curriculum Vitae; ELAM – Executive Leadership in Academic Medicine programme; EWIM – Early-career Women In Medicine program; GP – General Practitioner; MWIM – Mid-career Women In Medicine program; NR – Not reported; PRIME – Primary Medical Education program; RCT – Randomised control trial; UK – United Kingdom; URM – Underrepresented minority (study defined); USA – United States of America

For peer review only

For peer review only

Appendix 2: Quality assessment details

Randomised control trial

The single eligible randomised control trial had a low risk of bias for random sequence generation, blinding of outcome assessment, and incomplete outcome data items.[24] Participant blinding showed a high risk of bias as participants knew which groups they were assigned to. A risk of recruitment bias was also evident due to the cluster-randomised design. Allocation concealment and selective outcome reporting were unclear.[24]

Case-control studies

One of the two identified case-control study was rated medium to high quality for all items.[38] The other case-control study was of lower overall quality as it demonstrated a high risk of selection bias for intervention participant selection, and no methodological or analytical adjustments were made for comparing intervention and control groups.[23]

Cohort studies

The majority of the 25 included cohort studies had representative intervention populations,[16 18 19 21 22 26-32 34-37 40 41 43-45] appropriate control groups,[16 18-22 26 27 29-37 39 41-44] high quality outcome assessment methods,[16 18 21 26-28 31 33-35 37 42-44] and long follow-up periods.[16 18-22 26-29 31-33 35-37 39-41 43 44] However, comparability was mostly deemed poor as groups were often unmatched.[16 19 22 26 28 29 32 34-36 39 42-44] Only one study demonstrated high quality on all items,[21] whilst two studies scored positively on eight out of nine items, but failed to provide cohort follow-up information.[18 37] There were additional identified risks in group selection as several studies incorporated pre-selected or highly motivated populations for intervention groups (for example, Dannels et al.[20]).

Qualitative methodology studies

The majority of qualitative studies were of high quality for demonstrating congruity between the research methodology used and the: i) research question,[46-53 55-59 61] ii) data collection methods,[46-53 55-58 61 62] iii) data analysis and presentation,[46-53 55 57 58 61] and iv) interpretation of results.[46-53 55-59 61 62] However, inadequate reporting of important information was frequent with studies failing to provide statements relating to the relationship between the researchers and research itself.[46 49 51-58 61 62] Only one study demonstrated high quality on all items of the quality assessment tool.[50]

Realist evaluation

The realist evaluation study was rated medium to high quality on the majority of the criteria.[60] Only three aspects of the evaluation were deemed inadequate, which all related to incomplete methodological reporting, especially surrounding data analysis.[60]

Mixed methods studies

The two controlled mixed methods studies were deemed high quality on the majority of criteria.[17 25] Notably, Campion et al.,[17] demonstrated a lack of reporting for complete outcome data and failed to provide high quality methodological approaches for both the qualitative and quantitative components of the study. Guevara et al.,[25] failed to provide a rationale for using a mixed methods design. Finally, it was unclear whether the intervention was conducted as intended in both studies.[17 25]

For peer review only

For peer review only

Appendix 3: Table of outcome data for studies within quantitative analysis

First author (Year of publication)	Intervention group*	Control group*	Outcome	Sample size: intervention group	Sample size: control group	Estimate (95% CI/ p value/ SD)	Estimate adjusted for
Aspiration							
Dannels (2008)[20]	Programme participants	Two control groups: Applied but not accepted non-programme participants & general mid-career faculty women (also non-participants)	Aspiration to higher leadership position within an academic health centre (academic medicine)	55	27 & 178	76.4% vs 63.0% vs 49.4% (chi-squared= 12.903, p=0.002)	Unadjusted but faculty control group matched on academic rank, department chair status, race/ethnicity, discipline, degree type, basic sciences vs clinical department, age, medical school ownership, and awards ranking.
Career Satisfaction							
Campion (2016)[17]	Intervention participants	Reference group (non-participants)	Career satisfaction post-intervention	10	12	N=9 satisfied or very satisfied vs N=12 satisfied or very satisfied (NR)	Unadjusted
Grisso (2017)[24]	Intervention group	Control group (no intervention)	Work self-efficacy change scores	62	70	0.18 vs 0.24 (p=0.642)	Statistical tests were adjusted to account for correlation induced by the clustered design using generalized estimating equations. Both within-person factors (age, years in rank, race) and unit-level factors (intervention assignment) were modelled simultaneously
	Intervention group	Control group (no intervention)	Work-family conflict change (TWIF scores)	62	70	-0.13 vs -0.05 (p=0.541)	
	Intervention group	Control group (no intervention)	Work-family conflict change (SWIF scores)	62	70	-0.20 vs -0.23 (p=0.879)	
	Intervention group	Control group (no intervention)	CCWAS culture score change	Unclear; 109 in total	Unclear; 109 in total	0.03 vs 0.13 (p=0.274)	
Winn (2018)[41]	Academy participants	Pre-academy participants	Percentage of people who felt that the BCRP have been quite or extremely supportive in helping people to make career decisions	24	22	63% vs 45% (p=0.37)	Unadjusted
	Academy participants	Pre-academy participants	Percentage of people who agree or strongly	25	22	76% vs 63% (p=0.54)	

For peer review only

First author (Year of publication)	Intervention group*	Control group*	Outcome	Sample size: intervention group	Sample size: control group	Estimate (95% CI/ p value/ SD)	Estimate adjusted for
			agree that the BCRP guides and supports residents in pursuing and presenting scholarly projects				
Skills & Knowledge							
Campion (2016)[17]	Intervention participants	Reference group (non-participants)	Knowledge, Skills, and Attitudes Survey	9	11	No overall estimate. For 13 out of 32 items on the scale, participants showed greater gains than the reference group (NR)	Unadjusted
	Intervention participants	Reference group (non-participants)	Change in Sense of Community Index score (SCI-2 total score)	10	12	Mean change 10.6 vs 3.83 (p=0.2, SD=13.7 vs 9.40)	
Kohlwes (2006)[29]	Programme residents (PRIME)	Non-PRIME programme residents	Clinical competence score (average on a 9 point scale)	32	2294	8.23 vs 8.09 (p<0.001)	Unadjusted
Löwe (2008)[45]	Intervention residents	Control residents (from two different locations)	Mean change in total methodological research knowledge score from baseline	15	22	8.4 vs 1.2 (effect size =2.5, p<0.001, SD=2.9 vs 2.8)	Unadjusted
	Intervention residents	Control residents (from two different locations)	Mean change in total self-assessment research competence score from baseline	15	22	0.4 vs -0.4 (effect size=1.1, p=0.01, SD=0.9 vs 0.7)	

For peer review only

First author (Year of publication)	Intervention group*	Control group*	Outcome	Sample size: intervention group	Sample size: control group	Estimate (95% CI/ p value/ SD)	Estimate adjusted for
Winn (2018)[41]	Academy participants	Pre-academy participants	Percentage of people who agree or strongly agree that they have learned how to present a poster through BCRP activities	24	22	50% vs 29% (p=0.07)	Unadjusted
	Academy participants	Pre-academy participants	Percentage of people who agree or strongly agree that they have learned how to give a presentation/talk through BCRP activities	24	22	75% vs 36% (p=0.02)	
	Academy participants	Pre-academy participants	Percentage of people who agree or strongly agree with feeling prepared for non-clinical (scholarly) work after residency	24	22	63% vs 45% (p=0.37)	
Funding							
Goldenberg (2012)[23]	Programme trainees	Non-programme trainees	Annual grant dollars received (excluding fellowship award funding) at 3-4 years post-fellowship	11	9	Median $80,000 vs $23,000 (p=0.2)	Unadjusted
	Programme trainees	Non-programme trainees	Percentage of individuals who obtained K award within 5 years of beginning fellowship	9	8	33% vs 0% (p=0.21)	Unadjusted
Grisso (2017)[24]	Intervention group	Control group (no intervention)	Percentage of individuals with improved grants	62	70	Rate Ratio 0.75 (95% CI: 0.54-1.03, p=0.08)	Statistical tests were adjusted to account for correlation induced by the clustered design using generalized estimating equations.

First author (Year of publication)	Intervention group*	Control group*	Outcome	Sample size: intervention group	Sample size: control group	Estimate (95% CI/ p value/ SD)	Estimate adjusted for
							Both within-person factors (age, years in rank, race) and unit-level factors (intervention assignment) were modelled simultaneously
Guevara (2018)[25]	Interview applications scholars	Interview applications non-scholars	Total number of grants (mean)	76	48	2.0 vs 0.7 (p<0.001, SD=1.5 vs 1.0)	Adjusted for age, gender, race/ethnicity, application year, change of institutions, and rank at time of application
	Interview applications scholars	Interview applications non-scholars	Total grant dollars in thousands (mean)	76	48	$1,463 vs $567 (p=0.02, SD=$2,390 vs $1,507)	
Libby (2016)[31]	Programme participants (assistant professors)	Matched controls (non-programme participants, assistant professors)	Number of grant awards - change in mean before and after intervention	25	125	0.20 to 1.15 vs 0.30 to 0.49 (p<0.01)	Unadjusted but controls matched for key variables such as time in rank and dollars awarded
	Programme participants (assistant professors)	Matched controls (non-programme participants, assistant professors)	Amount of funding associated with grant awards - change in mean before and after intervention	25	125	$21,580 to $105,008 vs $26,742 to $53,716 (p<0.01)	
	Programme participants (assistant professors)	Matched controls (non-programme participants, assistant professors)	Percentage grant success rate (no. awards/no. proposals) - change before and after intervention	25	125	46.9% to 52.9% vs 42.5% to 51.7% (p=0.78)	
Löwe (2008)[45]	Intervention residents	Control residents (from two different locations)	Percentage of people who had grant applications accepted for funding	15	22	33% vs 0% (p=0.007)	Unadjusted
Sweeny (2019)[44]	After programme implementation participants	Before programme implementation participants	Number of grants awarded to new investigators	NR	NR	30 vs 20 (NR)	Unadjusted

First author (Year of publication)	Intervention group*	Control group*	Outcome	Sample size: intervention group	Sample size: control group	Estimate (95% CI/ p value/ SD)	Estimate adjusted for
Research Participation							
Brandt (2018)[16]	Research residents	Clinical residents	Percentage of participants actively engaged in research after intervention completion	43	284	42% vs 29% (p=0.04)	Unadjusted
Goldenberg (2012)[23]	Programme trainees	Non-programme trainees	Percentage of time spent in research post-fellowship	11	9	Median 80% vs 55% (p=0.01)	Unadjusted
Grisso (2017)[24]	Intervention group	Control group (no intervention)	Change in the number of total hours worked per week	62	70	-3.82 vs -1.39 (p=0.006)	Statistical tests were adjusted to account for correlation induced by the clustered design using generalized estimating equations. Both within-person factors (age, years in rank, race) and unit-level factors (intervention assignment) were modelled simultaneously
Harrison (2020)[26]	Participants after intervention implementation	Participants before intervention implementation	Total number of presentations (mean) - *just residents*	Unclear	Unclear	24.6 vs 3.0 (p=0.002, SD=10.24 vs 1.58)	Unadjusted
	Participants after intervention implementation	Participants before intervention implementation	Grant submissions (mean) - *just residents*	Unclear	Unclear	0.8 vs 0.0 (NR)	
	Participants after intervention implementation	Participants before intervention implementation	Total number of presentations (mean) - *just faculty*	Unclear	Unclear	40.0 vs 8.2 (p=0.025, SD=20.51 vs 1.30)	Unadjusted
Kohlwes (2016)[30]	Programme residents (PRIME)	Non-PRIME programme residents	Percentage of alumni who identified their primary role as a clinician investigator	71	98	35.2% vs 28.6% (chi-squared=0.843, p=0.358)	Unadjusted
	Programme residents (PRIME)	Non-PRIME programme residents	Mean percentage of professional time devoted	71	98	26.2% vs 20.6% (p=0.255)	

For Peer review only

First author (Year of publication)	Intervention group*	Control group*	Outcome	Sample size: intervention group	Sample size: control group	Estimate (95% CI/ p value/ SD)	Estimate adjusted for
			to clinical or translational research				
Löwe (2008)[45]	Intervention residents	Control residents (from two different locations)	Percentage of people currently writing a journal article during 1 year program	15	22	86.7% vs 36.4% (p=0.003)	Unadjusted
	Intervention residents	Control residents (from two different locations)	Percentage of people currently writing a book article during 1 year program	15	22	6.7% vs 13.6% (p=0.63)	
	Intervention residents	Control residents (from two different locations)	Percentage of people who did presentations at scientific meetings during the last year	15	22	80% vs 40.9% (p=0.04)	
	Intervention residents	Control residents (from two different locations)	Percentage of people who submitted one or more grant proposal with first authorship in the last year	15	22	6.7% vs 0% (p=0.41)	
	Intervention residents	Control residents (from two different locations)	Percentage of people who submitted one or more grant proposal with co-authorship in the last year	15	22	46.7% vs 4.6% (p=0.004)	
**Mandel (2018)[32]	Graduates after residency programme transition	Graduates before residency programme transition	Percentage of graduates whose research activities were dedicated to clinical research during residency	19	9	84% vs 33% (p=0.013)	Unadjusted
	Graduates after residency programme transition	Graduates before residency programme transition	Percentage of graduates whose research activities were dedicated to full time basic	19	9	33% vs 22% (p=0.68)	

For peer review only

First author (Year of publication)	Intervention group*	Control group*	Outcome	Sample size: intervention group	Sample size: control group	Estimate (95% CI/ p value/ SD)	Estimate adjusted for
			science/translational research during residency				
	Graduates after residency programme transition	Graduates before residency programme transition	Percentage of graduates whose current practice included clinical research	19	9	79% vs 0% (p<0.001)	
	Graduates after residency programme transition	Graduates before residency programme transition	Percentage of graduates whose current practice included laboratory research	19	9	5% vs 0% (p=1)	
	Graduates after residency programme transition	Graduates before residency programme transition	Percentage of graduates whose current practice included academic practice	19	9	44% vs 0% (p=0.026)	
Sweeny (2019)[44]	After programme implementation participants	Before programme implementation participants	Number of research active emergency healthcare providers	NR	NR	181 vs 23 (NR)	Unadjusted
	After programme implementation participants	Before programme implementation participants	Number of presentations by emergency clinicians	NR	NR	61 vs 6 (NR)	
Retention & Promotion							
Chang (2016)[18]	Attendants from 1 of 3 career development programmes (all female)	Matched non-participants (all female)	Retention as assistant professor role (since first year of appointment)	732	4962	Log rank chi-squared 640.95, df=1 (p<0.001)	Adjusted for years in academic medicine, age, tenure track status, degree type, department type
	Attendants from 1 of 3 career development programmes (all female)	Matched non-participants (all female)	Likelihood of departure from academic medicine - *just assistant professors*	Unclear	Unclear	HR 0.85 (95% CI: 0.74-0.98)	

First author (Year of publication)	Intervention group*	Control group*	Outcome	Sample size: intervention group	Sample size: control group	Estimate (95% CI/ p value/ SD)	Estimate adjusted for
	Attendants from 1 of 3 career development programmes (all female)	Matched non-participants (all female)	Likelihood of departure from academic medicine - *just associate professors*	Unclear	Unclear	HR 0.76 (95% CI: 0.64-0.93)	
	Attendants from 1 of 3 career development programmes (all female)	Matched non-participants (all female)	Likelihood of departure from academic medicine - *just full professors*	Unclear	Unclear	HR 0.68 (95% CI: 0.50-0.92)	
	Attendants from 1 of 3 career development programmes (all female)	Matched non-participants (all female)	Departure from academic medicine after 10 years - *just assistant professors*	732	4962	20% vs 33% (NR)	
	Attendants from 1 of 3 career development programmes (all female)	Matched non-participants (all female)	Departure from academic medicine after 10 years - *just associate professors*	429	884	10% vs 17% (NR)	
	Attendants from 1 of 3 career development programmes (all female)	Matched non-participants (all female)	Departure from academic medicine after 10 years - *just full professors*	215	172	8% vs 17% (NR)	
	Attendants from 1 of 3 career development programmes (all female)	Matched non-participants (all female)	Long term retention in academic medicine - *just assistant professors*	732	4962	Intervention participants were less likely to leave academia in the periods 0-13 years after their appointment and	

For peer review only

First author (Year of publication)	Intervention group*	Control group*	Outcome	Sample size: intervention group	Sample size: control group	Estimate (95% CI/ p value/ SD)	Estimate adjusted for
						22 years after or longer (NR).	
	Attendants from 1 of 3 career development programmes (all female)	Matched non-participants (all female)	Long term retention in academic medicine - *just associate professors*	429	884	Intervention participants were less likely to leave academia in the periods 0-10 years after their appointment (NR)	
	Attendants from 1 of 3 career development programmes (all female)	Matched non-participants (all female)	Long term retention in academic medicine - *just full professors*	215	172	Intervention participants were less likely to leave academia in any period after their initial appointment (NR)	
Daley (2006)[19]	Junior faculty after implementation - intervention participants	Junior faculty before implementation - non-intervention participants	Percentage of URM junior staff retained at the faculty	15	12	87% vs 58% (z statistic=1.69, p=0.091)	Unadjusted
	Junior faculty after implementation - intervention participants	Junior faculty before implementation - non-intervention participants	Percentage of URM junior staff retained in academic medicine	15	12	93% vs 75% (z statistic=1.33, p=0.184)	
Ehlers (2018)[21]	Programme participants	Matched non-programme participants	Enter private practice post-programme	70	70	40% vs 38.6% (p=0.862)	Time since matriculation into fellowship was included as a covariate. Matching criteria included fellowship program (CV or GI), gender, years of post-MD graduate training (±1 year for 65 pairs and ±3 years for 5 pairs), age at the time of starting fellowship
	Programme participants	Matched non-programme participants	Enter academic practice post-programme	70	70	60% vs 61.4% (p=0.862)	

First author (Year of publication)	Intervention group*	Control group*	Outcome	Sample size: intervention group	Sample size: control group	Estimate (95% CI/ p value/ SD)	Estimate adjusted for
							training (±3 years), and site of fellowship (2 pairs needed to be matched across sites)
Emans (2008)[22]	Faculty 3 years after implementation	Faculty 3 years before implementation	Number of promotions - *male faculty only*	Unclear	Unclear	17% increase (NR)	Unadjusted
	Faculty 3 years after implementation	Faculty 3 years before implementation	Number of promotions - *female faculty only*	Unclear	Unclear	56% increase (NR)	Unadjusted
	Faculty 3 years after implementation	Faculty 3 years before implementation	Number of promotions - *URM faculty only*	Unclear	Unclear	60% increase (NR)	Unadjusted
	Faculty 3 years after implementation	Faculty 3 years before implementation	Number of promotions - *Asian faculty only*	Unclear	Unclear	60% increase (NR)	Unadjusted
Guevara (2018)[25]	Interview applications scholars	Interview applications non-scholars	Percentage of people who received any kind of position promotion	76	48	67.1% vs 58.3% (p=0.32)	Adjusted for age, gender, race/ethnicity, application year, change of institutions, and rank at time of application
	Interview applications scholars	Interview applications non-scholars	Retention in academic position (% of people)	76	48	84.2% vs 75% (p=0.21)	
Kohlwes (2006)[29]	Programme residents (PRIME)	Other program residents (UCSF internal medicine programs)	Percentage of people asked to be a chief resident	32	185	21.8% vs 9.2% (p=0.03)	Unadjusted
Kohlwes (2016)[30]	Programme residents (PRIME)	Non-PRIME programme residents	Percentage of alumni with an academic appointment	71	98	71.4% vs 67% (chi-squared=0.370, p=0.5430	Unadjusted
	Programme residents (PRIME)	Non-PRIME programme residents	Influence of research experience during residency on career choice (% strongly agree	71	98	63.4% vs 46.4% (chi-squared=4.757, p=0.029)	

For peer review only

First author (Year of publication)	Intervention group*	Control group*	Outcome	Sample size: intervention group	Sample size: control group	Estimate (95% CI/ p value/ SD)	Estimate adjusted for
			or agree on questionnaire)				
	Programme residents (PRIME)	Non-PRIME programme residents	Influence of success with research during residency on career choice (% strongly agree or agree on questionnaire)	71	98	36.6% vs 23.5% (chi-squared=3.458, p=0.063)	
Ockene (2017)[35]	Programme participants	Non-programme participants	Percentage of people promoted to associate professor	29	188	72% vs 32% (p<0.001)	Adjusted for equivalent time in rank
Ries (2009)[37]	NCLAM programme participants	Non-NCLAM programme participants hired during same period	Faculty retention after 8-year probationary period (univariate analysis)	120	719	HR=1.77 (95% CI: 1.20-2.61, p=0.004)	Unadjusted
	NCLAM programme participants	Non-NCLAM programme participants hired during same period	Faculty retention after 8-year probationary period (multivariate analysis)	120	719	HR = 1.67 (95% CI: 1.11-2.50, p=0.01)	Date of hire, gender and URM faculty were included as covariates
Ries (2012)[38]	NCLAM programme participants (1999-2006)	Matched non-NCLAM programme participants (1999-2006)	8-year probationary period retention rate (% of people retained)	113	202	67% vs 56% (p=0.04)	Matched for: (1) gender, (2) academic series (research versus clinical primary job description), (3) initial academic rank/step (academic experience when hired), (4) hire date (within 12 months, to control for temporal changes in internal and external institutional variables), and (5) department
Recruitment							
Brandt (2018)[16]	Research residents	Clinical residents	Percentage of people who entered first job in academic practice	43	284	34% vs 20% (p=0.0001)	Unadjusted
	Research residents	Clinical residents (before	Percentage of people	43	87	20% vs 14% (NR)	Unadjusted

For peer review only

First author (Year of publication)	Intervention group*	Control group*	Outcome	Sample size: intervention group	Sample size: control group	Estimate (95% CI/ p value/ SD)	Estimate adjusted for
	(after implementing programme)	implementing programme)	who currently or once held a full-time academic appointment				
Dannels (2008)[20]	Programme participants	Two control groups: Applied but not accepted non-programme participants & general mid-career faculty women (also non-participants)	Highest rank/ position professor	53	25 & 172	69.8% vs 48% vs 68.6% (NR)	Unadjusted but faculty control group matched on academic rank, department chair status, race/ethnicity, discipline, degree type, basic sciences vs clinical department, age, medical school ownership, and awards ranking.
	Programme participants	Two control groups: Applied but not accepted non-programme participants & general mid-career faculty women (also non-participants)	Highest rank/ position department chair/ director	52	27 & 160	25% vs 18.5% vs 15.6% (NR)	
	Programme participants	Two control groups: Applied but not accepted non-programme participants & general mid-career faculty women (also non-participants)	Highest rank/ position dean and above	52	27 & 160	11.5% vs 11.1% vs 4.4% (NR)	
	Programme participants	Two control groups: Applied but not accepted non-programme participants & general mid-career faculty women (also non-participants)	Academic title of chair or above	52	27 & 160	63.5% vs 37.0% vs 22.5% (chi-squared=29.96, p<0.001)	
Emans (2008)[22]	Faculty 5 years	Faculty year of	Percentage of professors	Unclear	Unclear	12% vs 14% (NR)	Unadjusted

For peer review only

First author (Year of publication)	Intervention group*	Control group*	Outcome	Sample size: intervention group	Sample size: control group	Estimate (95% CI/ p value/ SD)	Estimate adjusted for
	after programme implementation	programme implementation	- *female only*				
	Faculty 5 years after programme implementation	Faculty year of programme implementation	Percentage of associate professors - *female only*	Unclear	Unclear	30% vs 21% (p=0.023)	
	Faculty 5 years after programme implementation	Faculty year of programme implementation	Percentage of assistant professors - *female only*	Unclear	Unclear	44% vs 34% (NR)	
	Faculty 5 years after programme implementation	Faculty year of programme implementation	Percentage of instructors - *female only*	Unclear	Unclear	58% vs 53% (NR)	
	Faculty 5 years after programme implementation	Faculty year of programme implementation	Percentage of professors - *URM only*	Unclear	Unclear	3% vs 3% (NR)	
	Faculty 5 years after programme implementation	Faculty year of programme implementation	Percentage of associate professors - *URM only*	Unclear	Unclear	3% vs 2% (NR)	
	Faculty 5 years after programme implementation	Faculty year of programme implementation	Percentage of assistant professors - *URM only*	Unclear	Unclear	4% vs 5% (NR)	
	Faculty 5 years after programme implementation	Faculty year of programme implementation	Percentage of instructors - *URM only*	Unclear	Unclear	8% vs 8% (NR)	
	Faculty 5 years after programme implementation	Faculty year of programme implementation	Percentage of professors - *Asian only*	Unclear	Unclear	2% vs 0% (NR)	
	Faculty 5 years after programme implementation	Faculty year of programme implementation	Percentage of associate professors - *Asian only*	Unclear	Unclear	6% vs 3% (p=0.023)	
	Faculty 5 years after programme	Faculty year of programme	Percentage of assistant professors - *Asian only*	Unclear	Unclear	16% vs 9% (p=0.001)	

For peer review only

First author (Year of publication)	Intervention group*	Control group*	Outcome	Sample size: intervention group	Sample size: control group	Estimate (95% CI/ p value/ SD)	Estimate adjusted for
	implementation	implementation					
	Faculty 5 years after programme implementation	Faculty year of programme implementation	Percentage of instructors - *Asian only*	Unclear	Unclear	12% vs 12% (NR)	
Goldenberg (2012)[23]	Programme trainees (academic practice physicians only)	Non-programme trainees	Time from fellowship entry to assistant professor	11	9	Median 3.5 years vs 7 years (p<0.001)	Unadjusted
Joshua Smith (2014)[27]	Two intervention groups: Residents with protected research time and completing degree, & residents with just protected research time	Residents without protected research time	Percentage of residents for which their first job is an academic appointment	15 & 24	23	93.3% vs 58.3% vs 30.4% (p=0.046 comparing research time, p<0.001 comparing no research time)	Unadjusted
Khot (2011)[28]	NIH associates who entered academic medicine	Non-associates who entered academic medicine (identified from faculty roster database)	Academic rank achieved - full professor	1577	27821	Ratio 1.57 (95% CI: 1.49-1.66, p<0.001)	Unadjusted
	NIH associates who entered academic medicine	Non-associates who entered academic medicine (identified from faculty roster database)	Academic rank achieved - chair	1577	27821	Ratio 2.0 (95% CI: 1.78-2.24, p<0.001)	
	NIH associates who entered academic medicine	Non-associates who entered academic medicine (identified from faculty roster database)	Academic rank achieved - dean	1577	27821	Ratio 2.97 (95% CI: 2.21-3.98, p<0.001)	

For peer review only

First author (Year of publication)	Intervention group*	Control group*	Outcome	Sample size: intervention group	Sample size: control group	Estimate (95% CI/ p value/ SD)	Estimate adjusted for
	NIH associates who entered academic medicine	Non-associates who entered academic medicine (identified from faculty roster database)	Active faculty appointments in the top 10 schools	626	6038	Ratio 1.34 (95% CI: 1.15-1.56)	
	NIH associates who entered academic medicine	Non-associates who entered academic medicine (identified from faculty roster database)	Active faculty appointments in the top 20 schools	626	6038	Ratio 1.47 (95% CI: 1.33-1.63)	
Sheridan (2010)[39]	Intervention-participating departments	Non-intervention participating departments	Percentage of female faculty hired before and after workshop implementation	17	9	Precise estimate not reported - see Figure 1 in paper for details (p<0.05)	Unadjusted
Sweeny (2019)[44]	After programme implementation participants	Before programme implementation participants	Number of new principal investigators recruited	NR	NR	55 vs 17 (NR)	Unadjusted
Valantine (2014)[40]	University faculty where programme was implemented	Faculty at 6 peer universities	Change in female faculty as a percentage of total faculty; *including assistant, associate, and full professors* (2004-2010)	1219	17000	5.8% vs 4.5% (p=0.011)	Unadjusted
	University faculty where programme was implemented	Faculty at 6 peer universities	Change in female faculty as a percentage of total faculty; *just assistant professors* (2004-2010)	420	7362	4.7% vs 5.0% (p=0.573)	
	University faculty where programme was implemented	Faculty at 6 peer universities	Change in female faculty as a percentage of total faculty; *just associate professors* (2004-2010)	370	4546	5.8% vs 5.1% (p=0.385)	

For peer review only

First author (Year of publication)	Intervention group*	Control group*	Outcome	Sample size: intervention group	Sample size: control group	Estimate (95% CI/ p value/ SD)	Estimate adjusted for
	University faculty where programme was implemented	Faculty at 6 peer universities	Change in female faculty as a percentage of total faculty; *just full professors* (2004-2010)	429	5092	5.2% vs 4.0% (p=0.003)	
	University faculty where programme was implemented	National faculty cohort	Change in female faculty as a percentage of total faculty; *including assistant, associate, and full professors* (2004-2010)	1219	116996	5.8% vs 4.0% (p=0.001)	Unadjusted
	University faculty where programme was implemented	National faculty cohort	Change in female faculty as a percentage of total faculty; *just assistant professors* (2004-2010)	420	56509	4.7% vs 3.6% (p=0.340)	
	University faculty where programme was implemented	National faculty cohort	Change in female faculty as a percentage of total faculty; *just associate professors* (2004-2010)	370	27842	5.8% vs 4.1% (p=0.868)	
	University faculty where programme was implemented	National faculty cohort	Change in female faculty as a percentage of total faculty; *just full professors* (2004-2010)	429	31645	5.2% vs 3.7% (p=0.001)	
Publications							
Brandt (2018)[16]	Research residents	Clinical residents	Publication productivity over career	43	284	Median 14 vs 4 (p<0.0001)	Unadjusted
	Research residents	Clinical residents	Publication productivity during residency	43	284	Median 10 vs 2.5 (p<0.0001)	
Ehlers (2018)[21]	Programme participants	Matched non-programme participants	Number of publications at 5-years post-fellowship	70	70	Median 8 vs 5 (p=0.041)	Matching criteria included fellowship program (CV or GI), gender, years of post-MD graduate training (±1 year for 65
	Programme participants	Matched non-programme participants	Number of first-author publications at 5-years	70	70	Median 4 vs 2 (p=0.002)	

For peer review only

First author (Year of publication)	Intervention group*	Control group*	Outcome	Sample size: intervention group	Sample size: control group	Estimate (95% CI/ p value/ SD)	Estimate adjusted for
			post-fellowship				pairs and ±3 years for 5 pairs), age at the time of starting fellowship training (±3 years), and site of fellowship (2 pairs needed to be matched across sites)
	Programme participants	Matched non-programme participants	H-Index (as of year when studies was conducted - January 2018)	70	70	Median 11 vs 7 (p=0.013)	Time since matriculation into fellowship was included as a covariate. Matching criteria included fellowship program (CV or GI), gender, years of post-MD graduate training (±1 year for 65 pairs and ±3 years for 5 pairs), age at the time of starting fellowship training (±3 years), and site of fellowship (2 pairs needed to be matched across sites)
Goldenberg (2012)[23]	Programme trainees	Non-programme trainees	Annual number of peer-reviewed publications 3-4 years post-fellowship	11	9	Median 3.5 vs 1 (p=0.01)	Unadjusted
Grisso (2017)[24]	Intervention group	Control group (no intervention)	Increase in total no. of publications 2009 to 2012	62	70	Rate Ratio 0.80 (95% CI: 0.63-1.02, p=0.07)	Statistical tests were adjusted to account for correlation induced by clustered design using generalized estimating equations. Both within-person factors (age, years in rank, race) and unit-level factors (intervention assignment) were modelled simultaneously
	Intervention group	Control group (no intervention)	Increase in first author publications 2009 to 2012	62	70	Rate Ratio 1.00 (95% CI: 0.67-1.50, p=0.99)	
	Intervention group	Control group (no intervention)	Increase in total no. of peer review publications 2009 to 2012	62	70	Rate Ratio 0.95 (95% CI: 0.68-1.33, p=0.78)	
	Intervention group	Control group (no intervention)	Increase in first author peer review publications 2009 to 2012	62	70	Rate Ratio 1.06 (95% CI: 0.58-1.95, p=0.85)	

For peer review only

First author (Year of publication)	Intervention group*	Control group*	Outcome	Sample size: intervention group	Sample size: control group	Estimate (95% CI/ p value/ SD)	Estimate adjusted for
Guevara (2018)[25]	Interview applications scholars	Interview applications non-scholars	Total no. of publications (mean) from point of application to December 2013	76	48	27.2 vs 33.0 (p=0.47, SD=30.0 vs 58.8)	Adjusted for age, gender, race/ethnicity, application year, change of institutions, and rank at time of application
	Interview applications scholars	Interview applications non-scholars	H-Index (as of December 2013)	76	48	12.5 vs 10.9 (p=0.32, SD=7.9 vs 10.2)	
Harrison (2020)[26]	Participants after intervention implementation	Participants before intervention implementation	Mean number of publications (in 5 years) - *residents only*	Unclear	Unclear	7.0 vs 4.4 (p=0.263, SD= 4.47 vs 1.82)	Unadjusted
	Participants after intervention implementation	Participants before intervention programme	Mean number of publications (in 5 years) - *faculty only*	Unclear	Unclear	16.6 vs 12.8 (p=0.197, SD=4.39 vs 4.15)	Unadjusted
Joshua Smith (2014)[27]	Two intervention groups: Residents with protected research time and completing degree, & residents with just protected research time	Residents without protected research time	Number of publications (mean)	17 & 27	24	10.3 vs 5.30 vs 1.29 (p=0.001 comparing to research time, p<0.001 comparing to no research time)	Unadjusted
	Two intervention groups: Residents with protected research time and completing degree, & residents with just protected research time	Residents without protected research time	Number of first-author publications (mean)	17 & 27	24	4.06 vs 2.30 vs 0.46 (p=0.017 comparing to research time, p<0.001 comparing to no research time)	
	Two intervention groups: Residents with protected	Residents without protected research time	Impact factor of publications (mean)	17 & 27	24	32.3 vs 17.8 vs 2.69 (p=0.001 comparing to	

For peer review only

First author (Year of publication)	Intervention group*	Control group*	Outcome	Sample size: intervention group	Sample size: control group	Estimate (95% CI/ p value/ SD)	Estimate adjusted for
	research time and completing degree, & residents with just protected research time					research time, p<0.001 comparing to no research time)	
	Two intervention groups: Residents with protected research time and completing degree, & residents with just protected research time	Residents without protected research time	Adjusted impact factor of publications (mean)	17 & 27	24	65.4 vs 36.1 vs 5.17 (p=0.005 comparing to research time, p<0.001 comparing to no research time)	Adjusted for level of authorship
Klimas (2017)[42]	Programme participants	Non-programme participants	Total number of published papers at the end of the one-year fellowship	4	4	7 vs 1 (p=0.1)	Unadjusted
Kohlwes (2016)[30]	Programme residents	Non-programme residents	Percentage of alumni who published research they had started during residency (first-author peer-review article)	71	92	64.3% vs 40.2% (chi-squared=9.213, p=0.002)	Unadjusted
Löwe (2008)[45]	Intervention residents	Control residents (from two different locations)	Percentage of people who completed one or more original publications with first authorship in the last year	15	22	46.7% vs 22.7% (p=0.13)	Unadjusted
	Intervention residents	Control residents (from two different locations)	Percentage of people who completed one or more original publications with co-authorship in the last year	15	22	60% vs 18.2% (p=0.01)	

For peer review only

First author (Year of publication)	Intervention group*	Control group*	Outcome	Sample size: intervention group	Sample size: control group	Estimate (95% CI/ p value/ SD)	Estimate adjusted for
	Intervention residents	Control residents (from two different locations)	Percentage of people who completed one or more review or meta-analysis with first authorship in the last year	15	22	0% vs 9.1% (p=0.5)	
	Intervention residents	Control residents (from two different locations)	Percentage of people who completed one or more review or meta-analysis with co-authorship in the last year	15	22	0% vs 9.1% (p=0.5)	
	Intervention residents	Control residents (from two different locations)	Percentage of people who completed one or more book article with first authorship in the last year	15	22	26.7% vs 18.2% (p=0.69)	
	Intervention residents	Control residents (from two different locations)	Percentage of people who completed one or more book article with co-authorship in the last year	15	22	20% vs 4.6% (p=0.28)	
Merani (2014)[43]	Additional degree trainees: Masters trainees, & PhD trainees	Clinical-only trainees	Percentage of surgeons involved in research publication during training with any authorship role	72 & 33	218	81.9% vs 100% vs 38.1% (p<0.05)	Unadjusted
	Additional degree trainees: Masters trainees, & PhD trainees	Clinical-only trainees	Percentage of surgeons involved in research publication during training with principal author role	72 & 33	218	58.3% vs 100% vs 27.1% (p<0.05)	
	Additional degree trainees: Masters	Clinical-only trainees	Percentage of surgeons involved in research	72 & 33	218	19.4% vs 12.1% vs 5% (p<0.05)	

First author (Year of publication)	Intervention group*	Control group*	Outcome	Sample size: intervention group	Sample size: control group	Estimate (95% CI/ p value/ SD)	Estimate adjusted for
	trainees, & PhD trainees		publication during training with senior author role				
	Additional degree trainees: Masters trainees, & PhD trainees	Clinical-only trainees	Percentage of surgeons involved in research publication post-training with any authorship role	69 & 32	201	81.2% vs 90.6% vs 44.3% (p<0.05)	
	Additional degree trainees: Masters trainees, & PhD trainees	Clinical-only trainees	Percentage of surgeons involved in research publication post-training with principal author role	69 & 32	201	56.5% vs 71.9% vs 26.4% (p<0.05)	
	Additional degree trainees: Masters trainees, & PhD trainees	Clinical-only trainees	Percentage of surgeons involved in research publications post-training with senior author role	69 & 32	201	37.7% vs 46.9% vs 14.9% (p<0.05)	
Mills (2011)[33]	Programme participants	Non-programme participants	Percentage of participants with publication after graduation	232	295	69% vs 34% (p<0.001)	Unadjusted
	Programme participants	Non-programme participants	Median number of publications after graduation	232	295	2 vs 0 (p<0.001)	
	Programme participants	Non-programme participants	Likelihood of publishing in the future	232	295	OR=3.7 (95% CI: 2.5-5.6)	Adjusted for gender and publications prior to programme
Nasab (2019)[34]	Programme participants	Non-programme participants (before implementation of programme)	Number of published papers during course period (2015-2018) - *just junior faculty members*	14	26	Mean 9.21 vs 4 (p=0.003, SD=8.1 vs. 5.21)	Unadjusted
	Programme participants	Non-programme participants (before implementation of	Number of published papers during course period (2015-2018) - *just*	35	11	Mean 4.31 vs 2 (p=0.008, SD=3.44 vs. 1.67)	

First author (Year of publication)	Intervention group*	Control group*	Outcome	Sample size: intervention group	Sample size: control group	Estimate (95% CI/ p value/ SD)	Estimate adjusted for
		programme)	*fellows*				
	Programme participants	Non-programme participants (before implementation of programme)	Journal impact factor during course period (2015-2018) - *just junior faculty members*	14	26	Mean 3.09 vs 4.57 (p=0.67, SD=1.54 vs. 5.03)	
	Programme participants	Non-programme participants (before implementation of programme)	Journal impact factor during course period (2015-2018) - *just fellows*	35	11	Mean 5.46 vs 3.03 (p=0.776, SD=9.81 vs. 1.72)	
Patel (2018)[36]	Programme cohorts (2011/2012 - 2014/2015)	Pre-programme cohorts (2007/2008 - 2010/2011)	Likelihood of publishing one or more clinical research articles (%)	35	36	77% vs 44% (chi-squared=7.9, p=0.005)	Unadjusted
	Programme cohorts (2011/2012 - 2014/2015)	Pre-programme cohorts (2007/2008 - 2010/2011)	Number of published clinical research articles during residency (mean)	35	36	2.09 vs 1.56 (NR)	

*Groups are reported as described by authors. **Inconsistent data reported and referred to in tables and text; data represented here is extracted from text.

Abbreviations: BCRP - Boston Combined Residency Programme; CCWAS - Culture that is conducive to women's academic success; NCLAM - National Center of Leadership in Academic Medicine; NR - Not Reported; PRIME - Primary Medical Education programme; SWIF - Strain-based work interference with family; TWIF - Time-based work interference with family; UCSF - University of California San Francisco; URM - Underrepresented Minorities.

For peer review only

PRISMA 2020 Checklist

Section and Topic	Item #	Checklist item	Location where item is reported
**TITLE**			
Title	1	Identify the report as a systematic review.	Page 1
**ABSTRACT**			
Abstract	2	See the PRISMA 2020 for Abstracts checklist.	Page 2
**INTRODUCTION**			
Rationale	3	Describe the rationale for the review in the context of existing knowledge.	Page 3
Objectives	4	Provide an explicit statement of the objective(s) or question(s) the review addresses.	Page 3
**METHODS**			
Eligibility criteria	5	Specify the inclusion and exclusion criteria for the review and how studies were grouped for the syntheses.	Page 3-4
Information sources	6	Specify all databases, registers, websites, organisations, reference lists and other sources searched or consulted to identify studies. Specify the date when each source was last searched or consulted.	Page 3
Search strategy	7	Present the full search strategies for all databases, registers and websites, including any filters and limits used.	Page 3 (and reported in full in published protocol)
Selection process	8	Specify the methods used to decide whether a study met the inclusion criteria of the review, including how many reviewers screened each record and each report retrieved, whether they worked independently, and if applicable, details of automation tools used in the process.	Page 4
Data collection process	9	Specify the methods used to collect data from reports, including how many reviewers collected data from each report, whether they worked independently, any processes for obtaining or confirming data from study investigators, and if applicable, details of automation tools used in the process.	Page 4
Data items	10a	List and define all outcomes for which data were sought. Specify whether all results that were compatible with each outcome domain in each study were sought (e.g. for all measures, time points, analyses), and if not, the methods used to decide which results to collect.	Page 4 (and reported in full in published protocol)
	10b	List and define all other variables for which data were sought (e.g. participant and intervention characteristics, funding sources). Describe any assumptions made about any missing or unclear information.	Page 4 (and reported in full in published protocol)
Study risk of bias assessment	11	Specify the methods used to assess risk of bias in the included studies, including details of the tool(s) used, how many reviewers assessed each study and whether they worked independently, and if applicable, details of automation tools used in the process.	Page 4
Effect measures	12	Specify for each outcome the effect measure(s) (e.g. risk ratio, mean difference) used in the synthesis or presentation of results.	Page 4, and Appendix 2
Synthesis methods	13a	Describe the processes used to decide which studies were eligible for each synthesis (e.g. tabulating the study intervention characteristics and comparing against the planned groups for each synthesis (item #5)).	Page 4
	13b	Describe any methods required to prepare the data for presentation or synthesis, such as handling of missing summary statistics, or data conversions.	Page 4

For peer review only - http://bmjopen.bmj.com/site/about/guidelines.xhtml

PRISMA 2020 Checklist

Section and Topic	Item #	Checklist item	Location where item is reported
	13c	Describe any methods used to tabulate or visually display results of individual studies and syntheses.	Page 4
	13d	Describe any methods used to synthesize results and provide a rationale for the choice(s). If meta-analysis was performed, describe the model(s), method(s) to identify the presence and extent of statistical heterogeneity, and software package(s) used.	Page 4
	13e	Describe any methods used to explore possible causes of heterogeneity among study results (e.g. subgroup analysis, meta-regression).	Page 4 (and reported in full in published protocol)
	13f	Describe any sensitivity analyses conducted to assess robustness of the synthesized results.	Page 4 (and reported in full in published protocol)
Reporting bias assessment	14	Describe any methods used to assess risk of bias due to missing results in a synthesis (arising from reporting biases).	Page 4
Certainty assessment	15	Describe any methods used to assess certainty (or confidence) in the body of evidence for an outcome.	N/A
**RESULTS**			
Study selection	16a	Describe the results of the search and selection process, from the number of records identified in the search to the number of studies included in the review, ideally using a flow diagram.	Page 4 and Figure 1
	16b	Cite studies that might appear to meet the inclusion criteria, but which were excluded, and explain why they were excluded.	N/A
Study characteristics	17	Cite each included study and present its characteristics.	Page 4-5 and Table 1
Risk of bias in studies	18	Present assessments of risk of bias for each included study.	Page 5 and appendix 1
Results of individual studies	19	For all outcomes, present, for each study: (a) summary statistics for each group (where appropriate) and (b) an effect estimate and its precision (e.g. confidence/credible interval), ideally using structured tables or plots.	Appendix 2
Results of syntheses	20a	For each synthesis, briefly summarise the characteristics and risk of bias among contributing studies.	Page 5-9
	20b	Present results of all statistical syntheses conducted. If meta-analysis was done, present for each the summary estimate and its precision (e.g. confidence/credible interval) and measures of statistical heterogeneity. If comparing groups, describe the direction of the effect.	N/A
	20c	Present results of all investigations of possible causes of heterogeneity among study results.	N/A
	20d	Present results of all sensitivity analyses conducted to assess the robustness of the synthesized results.	N/A
Reporting biases	21	Present assessments of risk of bias due to missing results (arising from reporting biases) for each synthesis assessed.	N/A
Certainty of evidence	22	Present assessments of certainty (or confidence) in the body of evidence for each outcome assessed.	N/A
**DISCUSSION**			
Discussion	23a	Provide a general interpretation of the results in the context of other evidence.	Page 10-11

For peer review only
For peer review only - http://bmjopen.bmj.com/site/about/guidelines.xhtml

PRISMA 2020 Checklist

Section and Topic	Item #	Checklist item	Location where item is reported
	23b	Discuss any limitations of the evidence included in the review.	Page 10-11
	23c	Discuss any limitations of the review processes used.	Page 10-11
	23d	Discuss implications of the results for practice, policy, and future research.	Page 10-11
**OTHER INFORMATION**			
Registration and protocol	24a	Provide registration information for the review, including register name and registration number, or state that the review was not registered.	Page 2 and 3
	24b	Indicate where the review protocol can be accessed, or state that a protocol was not prepared.	Page 3
	24c	Describe and explain any amendments to information provided at registration or in the protocol.	Page 3-4
Support	25	Describe sources of financial or non-financial support for the review, and the role of the funders or sponsors in the review.	Page 12
Competing interests	26	Declare any competing interests of review authors.	Page 12
Availability of data, code and other materials	27	Report which of the following are publicly available and where they can be found: template data collection forms; data extracted from included studies; data used for all analyses; analytic code; any other materials used in the review.	Page 12

From: Page MJ, McKenzie JE, Bossuyt PM, Boutron I, Hoffmann TC, Mulrow CD, et al. The PRISMA 2020 statement: an updated guideline for reporting systematic reviews. BMJ 2021;372:n71. doi: 10.1136/bmj.n71

For more information, visit: http://www.prisma-statement.org/

For peer review only

Strengthening the clinical academic pathway: a systematic review of interventions to support clinical academic careers for doctors and dentists

Journal:	*BMJ Open*
Manuscript ID	bmjopen-2021-060281.R1
Article Type:	Original research
Date Submitted by the Author:	21-Jun-2022
Complete List of Authors:	Raine, Gary; University of York, Centre for Reviews and Dissemination<br>Evans, Connor; University of York, Centre for Reviews and Dissemination<br>Uphoff, Eleonora; University of York, Centre for Reviews and Dissemination<br>Brown, Jennifer ; University of York, Centre for Reviews and Dissemination; University of York, Mental Health and Addiction Research Group, Department of Health Sciences,<br>Crampton, Paul; Hull York Medical School, Health Professions Education Unit<br>Kehoe, Amelia; Hull York Medical School, Health Professions Education Unit<br>Stewart, Lesley; University of York, Centre for Reviews and Dissemination<br>Finn, Gabrielle; Manchester University, School of Medical Sciences<br>Morgan, Jessica; University of York, Centre for Reviews and Dissemination; Leeds Children's Hospital, Department of Paediatric Haematology & Oncology
**Primary Subject Heading**:	Medical education and training
Secondary Subject Heading:	Medical education and training
Keywords:	EDUCATION & TRAINING (see Medical Education & Training), MEDICAL EDUCATION & TRAINING, HEALTH SERVICES ADMINISTRATION & MANAGEMENT

SCHOLARONE™
Manuscripts

I, the Submitting Author has the right to grant and does grant on behalf of all authors of the Work (as defined in the below author licence), an exclusive licence and/or a non-exclusive licence for contributions from authors who are: i) UK Crown employees; ii) where BMJ has agreed a CC-BY licence shall apply, and/or iii) in accordance with the terms applicable for US Federal Government officers or employees acting as part of their official duties; on a worldwide, perpetual, irrevocable, royalty-free basis to BMJ Publishing Group Ltd ("BMJ") its licensees and where the relevant Journal is co-owned by BMJ to the co-owners of the Journal, to publish the Work in this journal and any other BMJ products and to exploit all rights, as set out in our licence.

The Submitting Author accepts and understands that any supply made under these terms is made by BMJ to the Submitting Author unless you are acting as an employee on behalf of your employer or a postgraduate student of an affiliated institution which is paying any applicable article publishing charge ("APC") for Open Access articles. Where the Submitting Author wishes to make the Work available on an Open Access basis (and intends to pay the relevant APC), the terms of reuse of such Open Access shall be governed by a Creative Commons licence – details of these licences and which Creative Commons licence will apply to this Work are set out in our licence referred to above.

Other than as permitted in any relevant BMJ Author's Self Archiving Policies, I confirm this Work has not been accepted for publication elsewhere, is not being considered for publication elsewhere and does not duplicate material already published. I confirm all authors consent to publication of this Work and authorise the granting of this licence.

For peer review only

Strengthening the clinical academic pathway: a systematic review of interventions to support clinical academic careers for doctors and dentists

Gary Raine[1], Connor Evans[1], Eleonora Uphoff[1], Jennifer V E Brown[1,2], Paul E S Crampton[3], Amelia Kehoe[3], Lesley A Stewart[1], Gabrielle M Finn[4], and Jessica E Morgan[1,5]

Affiliations

1. Centre for Reviews and Dissemination, University of York, York, UK, YO10 5DD
2. Mental Health and Addiction Research Group, Department of Health Sciences, University of York, York, UK, YO10 5DD
3. Health Professions Education Unit, Hull York Medical School, University of York, York, UK, YO10 5DD
4. Division of Medical Education, School of Medical Sciences, Faculty of Biology, Medicine and Health, The University of Manchester, M13 9NT
5. Department of Paediatric Haematology & Oncology, Leeds Children's Hospital, Leeds, UK, LS1 3EX

Corresponding author: Jessica E Morgan, Centre for Reviews and Dissemination, University of York, York, UK, YO10 5DD, email: jess.morgan@york.ac.uk

Word count: 5787

For peer review only

Structured abstract

Objective:

Evaluate existing evidence on interventions intended to increase recruitment, retention and career progression within clinical academic (CA) careers, including a focus on addressing inequalities.

Design: Systematic review.

Data sources: MEDLINE, Embase, Cochrane Controlled Register of Trials (CENTRAL), PsycINFO, and Education Resource Information Center (ERIC) searched October 2019.

Study selection: Eligible studies included qualified doctors, dentists and/or those with a supervisory role. Outcomes were defined by studies and related to success rates of joining or continuing within a CA career.

Data extraction and synthesis: Abstract screening was supported by machine learning software. Full text screening was performed in duplicate, and study quality was assessed. Narrative synthesis of quantitative data was performed. Qualitative data were thematically analysed.

Results:

148 studies examined interventions; of which 28 were included in the quantitative synthesis, 17 in the qualitative synthesis, and two in both. Studies lacked methodological rigour and/or were hindered by incomplete reporting. Most were from North America. No study included in the syntheses evaluated interventions aimed at CA dentists.

Most quantitative evidence was from multi-faceted training programmes. These may increase recruitment, but findings were less clear for retention and other outcomes. Qualitative studies reported benefits of supportive relationships, including peers and senior mentors. Protected time for research helped manage competing demands on CAs. Committed and experienced staff were seen as key facilitators of programme success. Respondents identified several other factors at a programme, organisational or national level which acted as facilitators or barriers to success. Few studies reported on the effects of interventions specific to women or minority groups.

Conclusions:

Existing research is limited by rigour and reporting. Better evaluation of future interventions, particularly those intended to address inequalities, is required. Within the limits of the evidence, comprehensive multi-faceted programmes of training, including protected time, relational and support aspects, appear most successful in promoting CA careers.

Systematic Review Registration: Open Science Framework: https://osf.io/mfy7a

For peer review only

Strengths and limitations of this study

- This was a rigorous, systematic, and transparent review conducted by a highly experienced research team.
- Machine learning methodology facilitated very high-volume title and abstract screening to identify the most relevant records earlier than with traditional screening methods.
- Limitations in the reporting of the existing literature made synthesis challenging.
- It is unclear to what extent findings, which derived mostly from studies conducted in the USA, can be applied to other contexts. Multiple factors, including inter-country differences in organisational structures and practices, potentially limit the generalisability of findings.

Introduction

Clinical academics, individuals who work in both clinical and research roles, are a key part of the academic and healthcare workforce, combining expertise from both roles for the benefit of patients. Their work is diverse, including any health professional background, and varying amounts of research and teaching commitments, dependent on individual's career stage, role, and interests, as well as the healthcare, academic, and wider social systems in which they operate. Clinical academic roles can bring benefits to the individual (through variety of work and career satisfaction), to patients (who benefit from high quality research and research active institutions), and to their institutions (through their transferrable skills, funding income, and networks).[1-3]

The proportion of clinicians who choose a clinical academic career has fallen over time.[4] Furthermore, there are inequalities within this career path – with both gender and ethnicity differences being more pronounced than in either clinical or academic settings alone. [5] [6] In 2017, less than 20% of UK clinical academic professors were female, and less than 15% were of BME backgrounds. [6] Thus, interventions are needed which both increase the numbers of clinical academics and facilitate increased equality of opportunity for those who wish to pursue this career.

A previous systematic review (searches up to 2017) summarised quantitative evaluations of interventions to improve gender equality in any academic discipline. [7] It found that the evidence base was restricted by poor quality, and that interventions were limited to approaches that required time and effort from the women they intended to support ('bottom-up' approaches). [7] Interventions to address other inequalities within the clinical academic workforce have yet to be systematically explored and described. As such, there has been minimal synthesis of research into clinical academic careers to date, and this has been limited in both methodological approach (quantitative studies only) and scope, though has produced interesting findings.

Career pathways for clinical academics frequently separate doctors and dentists from other members of the healthcare workforce. Therefore, our systematic review aimed to identify, critically appraise, and synthesise research on existing interventions to increase recruitment, retention, and career progression in clinical academic medicine and dentistry. Whilst we evaluated interventions focused on gender equality, we also sought interventions to address inequalities related to characteristics other than gender. Our research aims to inform regulators and funders of the most effective interventions to support and promote clinical academic careers, with a view to increasing recruitment and retention within this group of healthcare professionals. Clinical academics may also use our findings to negotiate their roles and advocate for support from those providing oversight to their careers.

Methods

The systematic review protocol was registered with Open Science Framework (https://osf.io/mfy7a) and published. [8]

Search and information sources

The following databases were searched: MEDLINE (including MEDLINE Epub Ahead of Print, MEDLINE In-Process & Other Non-Indexed Citations, and MEDLINE Daily), Embase, Cochrane Controlled Register of Trials (CENTRAL), PsycINFO, and Education Resource Information Center (ERIC). Subject headings and free-text terms were used. Searches were limited to human studies published in English, from 2004 onwards (the introduction of the Athena SWAN initiative, a high-profile national programme aiming to improve gender equality across higher education).

We conducted two searches (see Appendix 1 for Medline strategy). One broad, sensitive search including terms relating to clinical academics. The other search was more specific and identified a subset of records from the broad search, by using terms relating to clinical academics and career development, recruitment, retention, and attrition. Full details of the search process are provided in the protocol. [8]

Inclusion and exclusion criteria

We included studies of qualified doctors, dentists and/or those with a supervisory role over these careers. [8] Studies of medical and dental students were not included. Studies of doctors and dentists who had completed their primary qualification but were undergoing further training (sometimes called specialty trainees, junior doctors, residents, fellows) were included, and are referred to as trainees within this manuscript. We included quantitative and qualitative studies evaluating interventions to increase recruitment to, and improve retention in, clinical academic careers, using study-defined outcome measures relating to rates of joining or continuing within clinical academia. Conference abstracts were excluded. Studies were limited to those performed in high-income countries, according to the World Bank classification, [9] in recognition of the cultural and organisational setting in which the research findings are to be applied.

Given the large numbers of potentially relevant studies identified during the screening process, additional exclusion criteria were introduced at the full-text screening stage to focus on the most relevant evidence. We excluded studies published before 2005, studies where the majority of the data were collected before 2004 (again to reflect the post-Athena SWAN era), and studies conducted in high-income countries with considerable differences in culture and/or health care provision compared with the UK, for example Singapore. The analysis plan was adjusted to enable a time and resource efficient reporting process. Only quantitative studies with a control group and qualitative studies using data derived predominantly from verbal data collection methods were included in a detailed synthesis of data.

Study selection and data extraction

We used the systematic review software Rayyan[10] to support the study selection process and employed our two-staged search process to train the built-in machine learning-based prioritisation function, with a view to more rapidly identifying potentially relevant records. Title and abstract screening took place in three stages, with all records from the most specific search and a randomly selected sample of the broader search screened in duplicate and used to train the Rayyan prioritisation function. The Rayyan prioritisation algorithm then supported the screening of the remaining records from the broader search. Screening stopped once the rate of potentially eligible records identified by the machine learning algorithm had fallen sufficiently from baseline.

Full-text screening was undertaken independently and in duplicate. At all stages, disagreements were resolved by a third reviewer or in team discussions.

Data were extracted by one researcher using standardised data extraction forms and independently checked by a second researcher. We extracted outcome data for only those studies that reported quantitative data and included a control group.

Quality assessment

Quality assessment of studies included in the synthesis used the Cochrane risk of bias tool for RCTs, [11] the Newcastle-Ottawa scale for non-randomised studies, [12] the Qualitative Assessment and Review Instrument Checklist for qualitative studies, [13] the Mixed Methods Appraisal Tool for mixed methods studies, [14] and the RAMESES II Quality Standards for Realist Evaluation. [15] Each study was individually assessed and checked by a second reviewer, and any conflicts resolved via discussion.

Data Synthesis

Data were summarised in narrative and tabular form. For accuracy of reporting, we retained the terminology used by included papers to describe their participants (which most often uses US terms). The frequently used American academic ranks of assistant professor and associate professor approximate those of lecturer and senior lecturer, respectively, in the UK.

Due to the heterogeneous nature of the studies, quantitative data were synthesised narratively, and qualitative data were synthesised based on the principles of thematic analysis. [16] All relevant qualitative findings were coded line-by-line by one researcher and codes subsequently reviewed by another researcher.

For peer review only

Codes were developed inductively and further refined as appropriate. Findings related to specific codes were brought together to identify cross-cutting themes and issues of potential relevance.

Patient and Public Involvement statement

Through Healthwatch York, [17] a member of the public was involved in the project steering group, influencing the inclusion criteria for the review to include international data, and informing the dissemination process.

Results

Study selection

Electronic databases were searched in October 2019 and returned a total of 34,230 records; 148 studies examined interventions; of which 28 were included in the synthesis of quantitative data, 17 in the qualitative synthesis, and data from two studies were included in both (Figure 1). The remaining 101 quantitative studies not including a control group were not synthesised further.

Study characteristics

Studies in the quantitative synthesis

Of the 30 studies included in the quantitative synthesis, 26 were conducted in the USA, [18-43] two in Canada, [44] [45] one in Australia, [46] and one in Germany. [47] Twenty-three were single centre programmes, [18] [19] [21-24] [26] [28] [29] [31-43] [47] and seven were national. [20] [25] [27] [30] [44-46]

No study included within the syntheses focused on clinical academic dentists. Otherwise, the populations studied were varied, in terms of grade, academic level and medical background of participants (see Appendix 2). Due to this high degree of variability, it was often difficult to determine the exact population investigated. Fourteen studies focused solely on participants who had completed their medical training, [19-22] [24] [26] [30] [32-34] [37] [40-42] 11 studied just trainees, [18] [23] [25] [28] [29] [31] [35] [43-45] [47] three included mixed populations, [27] [36] [38] and two were unclear/not reported. [39] [46]

Studies encompassed a diverse range of interventions. The majority evaluated complex interventions involving elements such as mentoring, protected research time, leadership training, and teaching workshops. Academic training programmes tended to focus on advancing trainee academic skills, research productivity and interest, whilst career development programmes (called faculty development programmes in some studies) centred on enhancing junior/senior faculty workforce within clinical academia through promotion, retention, and recruitment.

Study design also varied. There was one RCT, [26] two case-control designs, [25] [40] and 27 studies with a cohort design[18] [20-24] [28-39] [41-47] which included two studies using a mixed methods approach. [19] [27]

Four interventions had a gender-focus and were tailored specifically towards women, [20] [22] [26] [42] two towards ethnicity/underrepresented minority faculty in medicine, [21] [27] and two towards historically underrepresented faculty including women and minority populations. [24] [41]

Studies in the qualitative synthesis

Of the 19 studies included in the qualitative synthesis, 11 were conducted in the USA, [19] [27] [48-56] five in Canada, [57-61] and three in the UK. [62-64] Eight were from single institutions, [19] [53] [55-57] [59-61] and 11 were national level initiatives. [27] [48-52] [54] [58] [62-64] As in the quantitative synthesis, there was considerable diversity in the populations studied and the interventions involved (see Appendix 2). Ten of the included studies used qualitative methodology only, [49-52] [54] [56] [57] [59-61] eight studies used a mixed/multiple methods approach, [19] [27] [48] [53] [55] [58] [63] [64] and one study was described as a realist evaluation. [62]

Two interventions were aimed at women, [51] [54] one at individuals from ethnic groups underrepresented in medicine, [27] and another at “busy clinician educators”. [55] One study reported on an intervention targeted at participants who experienced substantial caregiving challenges. [52]

For peer review only

Quality assessment

Both quantitative and qualitative studies lacked methodological rigour and/or were hindered by incomplete reporting. Across all study types, intervention characteristics and population definitions were reported ambiguously. In most cohort studies there was minimal participant matching between intervention and control groups, and participant comparisons were often unadjusted or ill-defined. Group selection was equally problematic as many studies included pre-selected or highly motivated populations to receive the interventions, which may unintentionally bias results in favour of the intervention programmes. Nonetheless, the quantitative literature generally included appropriate follow-up methods and a large volume of outcome data suitable for analysis. The qualitative studies also provided rich data from relevant populations. However, comprehensive analysis plans and complete methodological reporting were not provided in most qualitative studies, and there was little reflexivity demonstrated. For further details on quality assessments, see Appendix 3.

Synthesis of quantitative data

All relevant reported outcomes were extracted and then grouped under eight broad categories relating to clinical academic careers (with categories created after the identification of outcome data). These categories were: aspiration, career satisfaction, skills & knowledge, research funding, research participation, recruitment, retention/promotion, and publication outcomes (full outcome data are provided in Appendix 4).

Aspiration

Only one study included a measure of aspiration. It found that significantly more participants attending a career development programme aspired to achieve a higher leadership position in academic medicine compared with non-attendees. [22]

Career satisfaction

Three studies reported outcomes relating to career satisfaction. [19] [26] [43] No single outcome demonstrated a statistically significant benefit for intervention participants versus controls, although results were favourable towards the intervention participants.

Skills and knowledge

Four studies reported outcomes relating to skills and knowledge. [19] [31] [43] [47] Significantly higher clinical competence scores, [31] and improved research competence scores[47] were found among participants of two academic training programmes. Methodological research knowledge also significantly improved for intervention participants in one study. [47] Significantly more intervention participants learned how to give a presentation in the study by Winn et al; [43] yet, within the same study there was no significant difference in the percentage of participants who learned how to present a poster or felt prepared for scholarly work after post-graduate training, compared with controls. No significant benefits were found for the Academy for Collaborative Innovation and Transformation career development programme. [19]

Research Funding

Outcomes relating to research funding were reported in six studies. [25-27] [33] [46] [47] Three studies found significant increases in the number of funded grants[27] [33] or the percentage of people with successful grant applications[47] for participants in programmes which combined research training with mentorship. Similar findings were non-significant in two training and development programmes. [25] [26] Another study found an increase in number of grant awards for intervention participants but it is unclear if this difference was statistically significant. [46] Two out of three studies measuring the amount of funding received (both amount of money and number of grants), found significantly greater amounts of funding awarded to participants attending career development programmes with mentorship compared with non-attendees. [27] [33]

For peer review only

Research participation

Research participation outcomes were reported in eight studies. [18] [25] [26] [28] [32] [34] [46] [47] Four studies included a measure of involvement in research activities; [18] [25] [32] [46] however, only two studies showed a significant increase in research participation – both studies assessed multi-faceted academic training programmes. [18] [25] Mandel et al., [34] found similar benefits for graduates taking part in a research-tailored curriculum intervention but it is unclear if this finding was significant. Two out of three measures relating to grant applications did not show any difference between groups in two interventions that included comprehensive research training. [28] [47] Löwe et al., [47] also found no benefit of a training programme on the number of participants writing a “book article” during residency.

Recruitment

Nine studies reported outcomes relating to recruitment in academia and achievement of a specific academic rank. [18] [22] [24] [25] [29] [30] [41] [42] [46] Two studies found that participants attending academic training programmes were significantly more likely to obtain their first job in academic practice post-training compared with non-attendees. [18] [29] Three studies showed that intervention participants were significantly more likely to be recruited at higher academic ranks, [22] and achieve higher ranks of assistant professor[25] or full professor[30] more quickly than participants not enrolled in academic training programmes. Female faculty recruitment increased in two intervention studies aimed at improving diversity. [41] [42] Valantine et al., [42] found significant increases in recruitment at full professor level but no significant increase was evident at assistant or associate professor level. Similarly, Emans et al., [24] found that female faculty recruitment for intervention participants only significantly increased at associate professor level opposed to non-significant increases for other academic levels.

Retention and promotion

Ten studies reported outcomes relating to retention, or promotion to a higher position. [20] [21] [23] [24] [27] [31] [32] [37] [39] [40] The retention of staff in clinical academia was significantly higher for participants in two career development programmes[39] [40] but non-significant in another career development programme, [27] and two academic training programmes. [23] [32] In one study, residents of an academic training programme with research experience were significantly more likely to choose academia as a future career than participants in the control group. [32] One study found significantly higher retention rates for assistant professors who took part in a career development programme tailored for women; [20] whilst a similar programme aiming to improve diversity found no difference in retention of underrepresented minority staff. [21] One mentorship programme found significantly higher rates of promotion to associate professor in the intervention group, [37] whilst another study found no increase in promotions for participants in the intervention group. [27]

Publications

Publication outcomes were reported in 14 studies, [18] [23] [25-29] [32] [35] [36] [38] [44] [45] [47] all of which measured the number of publications during or within five years of receiving an intervention. Nine studies found a significant increase in publication productivity, of which, eight were academic training programmes[18] [23] [25] [29] [32] [35] [45] [47] and one was a mentorship programme. [36] The other five studies found either non-significant differences[26-28] [44] or differences with no statistical analyses reported. [38] The number of first-author publications was reported in five studies; [23] [26] [29] [45] [47] but only three studies, all evaluating an academic training programme, showed a significant increase for intervention participants. [23] [29] [45] Publication impact was also measured across four studies, but only two studies demonstrated significantly higher H-index scores[23] or impact factors scores[29] for intervention participants taking part in an academic training programme. The number of peer review journal articles[26] and books published[47] were not found to be significantly different between intervention and control groups.

Synthesis of qualitative data

We identified seven themes:

For peer review only

Developing knowledge, skills and confidence in research and scholarship

Participants in seven studies reported improvements in research/scholarship knowledge and skills, and their confidence to conduct research activities, from planned teaching or learning sessions. [19] [48] [51] [55] [57] [59] [61] Respondents across three studies mentioned specific programme components as being useful for developing research/scholarship knowledge and skills. [48] [55] [61] The didactic sessions considered most helpful in one study focused on literature reviews, survey methodology and reference management. [55] Participants in another programme reported an unmet need for skills development including negotiation, grant management, and work-life balance, alongside a need for advice about a lack of faculty diversity and unconscious bias. [27]

Leadership skills and opportunities

Four studies reported positive findings about the leadership skills or opportunities that participants gained. [19] [27] [51] [54] Respondents in one study gained leadership experience with a women-focused national organisation at an earlier career stage than anticipated, and this helped some earn promotion. [54] Individuals in two studies pursued new leadership opportunities, or progressed their careers in other ways, after gaining self-confidence in career development programmes. [19] [51]

Personal characteristics and behaviour of individuals

Personal characteristics, including participants' level of personal ambition, enthusiasm, motivation, self-direction, interest, and commitment to the programme, influenced the decision to initially apply to a programme and/or participants' programme experiences in six studies. [49] [51] [56] [60] [63] [64] Some participants in the UK Academy of Medical Sciences mentoring scheme reported less successful relationships with mentors due, at least in part, to mentees not having been sufficiently proactive in arranging meetings and maintaining contact with mentors. [64] In the UK Academic Foundation Programme, good preparation by trainees facilitated success, including arranging early contact with supervisors and maintaining engagement at key stages of training. Conversely, one supervisor thought a barrier to success was that some candidates did not appreciate the time and work involved. [63]

Interactions and relationships

Networking through participation in career development initiatives, especially with individuals from other institutions, was valued for the benefits to career advancement and opportunities to identify mentors, and develop relationships with peers. [27] [51] [54] One study indicated that sponsorship was of benefit to women in terms of career advancement, including nominations for promotion and/or writing supportive references. [54]

Intervention participants gained emotional support, encouragement, self-confidence, and other benefits from relationships with peers in 11 studies. [19] [27] [48] [49] [51] [54-57] [61] [63] Interacting and engaging with peers reduced feelings of professional isolation[51] [54] [56] [61] and fostered a sense of community and belonging. [19] [54] [61] Women valued peer interaction, and the opportunity to share experiences, with other women. [49] [51] [54] Respondents also appreciated the professional collaboration that arose from peer relationships. [19] [48] [49] [51] [54] [57] [63] Some findings related to interactions explicitly described as peer mentoring. [19] [27] [49] [55] [61] Notably, we identified multiple terms used to describe peer interaction which made it difficult to draw clear distinctions between such terms and how they represent different forms of peer support.

Participants spoke positively about mentorship from senior colleagues. [27] [48-51] [54-57] [59] [60] [64] In addition to being a source of moral support, self-confidence and encouragement, mentors provided other assistance, including offering career-related advice; teaching research and scholarship skills; facilitating leadership opportunities; assisting with grant applications; suggesting ways of dealing with rejection and setbacks; and providing resources such as staff or equipment. [27] [48-50] [57] [64]

> "*When the challenges of combining clinical training and research clouded my judgment about future career steps, my mentor proved to be indispensable in making the most objective and adequate choice*". [64]

Respondents believed it was important to develop a 'network' or team of several mentors, drawing on the different strengths and areas of expertise of each mentor, and guarding against inadequate mentoring. [48] [49]

[54] Having at least one female mentor was important to some women in four studies, particularly in terms of having a role model, and providing guidance on balancing a career and family life. [49-51] [54] One study found mixed views on whether both mentor and mentee need to be from an ethnic group underrepresented in medicine. [27] A respondent in another study believed some individuals may experience difficulties in finding mentors of the same ethnicity. [49] Mentees in two studies believed they gained more objective and impartial advice from having mentors who were from a different institution. [49] [64] Trainees in one study preferred physicians over non-physicians as mentors, viewing them as role models. [60]

Studies reported mixed findings on the benefits of formalised mentorship programmes, [60] [64] and on the need for training of mentors and mentees. [27] [48] [63] [64]

Time and competing demands in clinical academia

Issues related to the time pressures experienced by clinical academics formed a consistent narrative across studies. Protected time was an important feature of career development programmes and training fellowships in seven studies. [19] [48] [52] [55] [57] [58] [60]

> *"Really what I needed was dedicated time so I'd have relief time from clinic to work on the project…it gave me a chance to… really move the project forward a lot more than I would have without it"*. [55]

One funding award enabled participants with substantial care-giving demands to gain greater control, flexibility, and choice over their time through buying more protected research time and hiring staff to take over various research-related tasks. [52] Awardees reported greater research productivity and an improved work-life balance. The award also assisted with career progression and retention in academia at critical time points. In another study, a participant found protected time facilitated achieving more publications. [48]

Some participants found it difficult in practice to maintain dedicated research time due to lack of clinical cover, and found that administrative staff were not always supportive which made scheduling protected time difficult. [52] [55] Time conflicts, particularly competing clinical demands, acted as a barrier to organising or participating in specific programme elements, research training or implementing training fellowships across four studies. [52] [55] [59] [60] The impact of the Athena SWAN programme was potentially undermined by institutions holding meetings at times that could be difficult for staff with caring responsibilities to attend. [62]

The competing commitments of clinical supervisors were barriers to success in the UK Academic Foundation Programme, [63] whilst in Canada, several Clinician Investigator Program programme directors did not have protected time for managing the programme. [58] Similarly, competing demands on senior faculty staff detracted from their ability to be a good mentor in another study. [49]

Facilitating programme participation and success

The influence of programme and organisational level factors on intervention participation and impact was identified across studies.

Having committed and experienced programme staff facilitated success in six studies. [19] [48] [55] [57] [58] [60] We interpreted 'programme staff' as individuals involved in programme delivery, including teachers, administrative staff, and programme leads.

Management support influenced programme participation in four studies. [19] [51] [58] [62] In one study, the provision of financial support to women faculty had enabled them to attend a career development programme. [51] In contrast, postdoctoral researchers perceived a lack of support from project leads and expressed scepticism that they would be given time to participate in Athena SWAN activities. [62]

In one study, most respondents supported the Athena SWAN programme and believed it had positive institutional outcomes. [62] However, the application process increased the workload of the mostly female self-assessment team, reinforcing institutional gender inequity and undermining the programme's aims. [62] A perceived emphasis on only supporting women was considered to perpetuate existing gendered social norms related to childcare provision. [62]

Two studies identified issues related to the promotion of programmes to staff. [51] [62] Participants had not accessed initiatives, as they were unaware of them or had ignored relevant information to prevent 'email overload'. [62] Elsewhere, a lack of institutional information promoting programmes meant faculty only learned about them from colleagues. [51]

Seven studies identified factors influencing success related to the delivery of learning sessions and training fellowships. [19] [48] [58-61] [63] Involvement in planning and developing sessions was considered important. [61] Various teaching methods were influential including: diverse educational methods; mixed guided and independent learning approaches; experiential learning; tailored coursework; and utilising the expertise of staff from different institutions. [19] [48] [59] [60] Other beneficial aspects of programmes included: a 'supportive' learning environment, high degree of autonomy in research training, flexibility, structure, and clear guidance. [58] [59] Barriers included some didactic content being judged as too jargonistic and delivered using inaccessible language. [19] Supervisors in the UK Academic Foundation Programme considered the short period of academic time a barrier. [63] Challenges related to infrastructure and logistics, for example the lack of suitable desk space, were found in two programmes. [56] [60]

At a national level, UK funding arrangements could undermine family-friendly policies implemented to support Athena SWAN, for example by not providing funding for maternity cover in grant awards. [62] Meanwhile, in Canada, inadequate funding for trainees was viewed as a barrier to Clinician Investigator Program entry. [58]

Funding and financial support

Funding for protected time and issues related to national policies are discussed earlier. Two studies suggested improvements in the intervention including funding administrative staff or research personnel[52] [55] and one reported that lack of funding resulted in limited access to specialist statistical support. [63] Funded fellowship programmes were reported to be more successful because they provided protected time for developing programme infrastructure. [60]

The need for bridge or seed funding was mentioned in some studies. [52] Funding requirements considering time worked rather than chronological time limits was also deemed important:

> "*[A]pplications [where] eligibility [is limited to] 3 years within starting your faculty position or 8 years within graduating [should] have this prorated…. so that the eligibility is based on time worked, not just a chronologic year, which may have a 3-month maternity leave… in it*". [52]

Mentorship could help address some of the financial challenges for junior clinical academics, through supporting grant application writing, helping with bridging funds, or providing research and administrative staff support. [50]

Discussion

Statement of principal findings

We identified few high quality, well-reported evaluations of interventions to improve recruitment or retention to clinical academia. Most studies were from North America and no controlled quantitative studies were from the UK. No studies included interventions for clinical academic dentists, and few included specific interventions for women or minority groups. No studies reported on outcomes related to patient benefit, or cost-effectiveness of interventions.

Most quantitative evidence derived from multi-faceted academic training programmes; such programmes may increase recruitment to academia among clinicians, but findings were less clear for retention or other outcomes related to participation in research and research funding. Qualitative studies reported benefits of supportive relationships for clinical academics, including peer and senior mentors. Formalised mentoring programmes were not universally considered useful. There was consistent evidence of the importance of having protected time, particularly to mitigate against the negative impact of competing clinical demands on research-related activity, though maintaining protected time could be difficult in practice. Across studies, committed and experienced programme staff were key facilitators of success. This study adds to the existing

literature by detailing more information about the evidence base of a broad range of interventions to increase recruitment and retention to CA careers, highlighting a clear gap in addressing inequalities.

Strengths and limitations of this review

This review used rigorous, systematic, and transparent methods conducted by a highly experienced research team. The broad focus provides insights for clinical academics, programme leads, and funders. We used machine learning methodology to facilitate very high-volume title and abstract screening to identify the most relevant records earlier, whilst resulting in better resource management by reducing the screening burden for the research team and reducing the time to review completion. As experience with machine learning grows, this may be further improved as confidence in the algorithms increases. We may have missed a small number of potentially relevant records (<1% of the total), which we do not anticipate to have impacted on the overall review results. Nonetheless, the high volume of potentially eligible records and limited time resource meant it was not possible to investigate all relevant primary studies in depth.

Limitations in the reporting of the existing literature made synthesis difficult. Many studies involved highly motivated participants compared with controls or participants who did not get accepted onto a programme as control groups. Such groups are not directly comparable leading to potentially biased results caused by baseline differences. Furthermore, it is unclear to what extent findings, which derived mostly from studies conducted in the USA, can be applied to other contexts. Multiple factors, including inter-country differences in organisational structures and practices, potentially limit the generalisability of findings. These challenges in extrapolating from literature limited by quality and geography hindered our ability to draw robust conclusions on the effectiveness of interventions designed to support clinical academic careers within the UK.

Our findings in relation to previous studies

This review includes a more substantial volume of literature than the previous review by Laver et al. [7] Our work confirms their findings that most studies are from the USA and that the quality of research in this field is generally poor. Compared with the previous systematic review, our review used a more comprehensive search strategy, and included interventions for all clinical academics, not just women. Our review had a broader focus than solely interventions to promote gender equality, but also found most evidence supported multi-faceted interventions and those with a mentoring component.

Our systematic review rigorously synthesises a previously disjointed body of evidence from a wide variety of methodologies and sources, thus presenting a coherent summary of the state of research landscape in this area. This goes beyond the previously available evidence about inequalities in clinical academic careers which often relied on small single centre studies, personal accounts, or routinely collected data. Policy and decision makers will be able to use our systematic review with confidence when planning and implementing future interventions.

Implications of this review for clinical academia and policy makers

The key implication identified by this review is that multi-faceted interventions are most likely to be successful in promoting clinical academic careers, with components such as protected time, mentorship, and supportive staff. The contribution of each facet of the intervention was not always clear due to incomplete reporting, and thus future work may consider using methods such as realist evaluation to distinguish the role each component plays in a programme’s ability to deliver key outcomes for clinical academics.

The lack of quantitative evidence of benefit should be carefully considered when planning future mentorship interventions, particularly the need for detailed evaluations. Whilst our qualitative synthesis showed mentoring of junior clinical academics by seniors was often considered beneficial, the quantitative data were less supportive of mentoring interventions, with some findings in favour, but others finding no evidence of benefit. Most evidence suggested mentorship improved funding received and supported programmes incorporating both peer and senior mentorship. Mentorship team composition may play a key role in the success of the relationship, including factors such as the number, gender, and location of mentors. Little evidence related to mentor ethnicity. Formalised mentorship schemes, including requiring specific mentor characteristics, were not clearly supported by the literature. Our findings may reflect the design of individual

For peer review only

programmes, or the studies evaluating them, but the need for further evaluation of mentorship programmes is clear.

Some studies suggested personal attributes of individual participants, such as commitment, enthusiasm, and motivation, could be key influences on intervention success, thus placing responsibility for programme failures on the individuals involved, rather than institutional or cultural factors. The complex interplay of gender, ethnicity, parenthood, and other protected characteristics on these attributes has not been explored within the included literature. Indeed, there was a notable absence of intersectional focus within the qualitative literature overall, with just two studies highlighting the interactions between gender and ethnicity. [52] [54] In the quantitative synthesis, few studies focused on interventions tailored towards women and/or underrepresented minority faculty, and none evaluated an intervention from an intersectional standpoint. Adopting an intersectionality perspective when developing and evaluating future strategies may address more effectively issues related to inequality within clinical academia.

Various factors at organisational or national levels had negative impacts on the success of interventions within this review. Such evidence indicates that the success of future initiatives will be limited unless action is taken to ensure that organisational practice and culture, as well as relevant national policies, support the recruitment, retention, and progression of clinical academics.

Implications of this review for research

From the identified evidence on interventions, it is clear that little benefit will be derived from conducting further small, single-centre cohort studies in this field. Future research should use more robust methods to evaluate the effectiveness of interventions over time, using a control group and outcomes relating to recruitment and retention for medical and dental academics. We recommend that research funders commit to establishing large scale national research infrastructure to facilitate this, spanning both clinical and academic environments.

Interventions evaluated through this infrastructure are most likely to be successful when embedded within comprehensive multi-faceted programmes, focused on developing relationships between clinical academics. Consideration should be given to evaluating support structures, including administration, personnel, and programme leadership, as well as the most effective timing of interventions along the clinical academic career path. Evaluations of structural and environmental factors should be prioritised over interventions targeting individual determinants.

Clear terminology for describing clinical academics, and co-produced core outcome sets for studies of clinical academic careers, would aid the synthesis of primary studies in future systematic reviews. To allow for a focus on equality and diversity, results should be open-access and transparently presented in disaggregated form, reporting gender and ethnicity differences. In addition, the intersectional perspective is notably absent from this field and further high-quality, reflexive, qualitative research should explore the interplay of multiple determinants of inequality.

Within this review, we identified an additional dataset of studies evaluating barriers and facilitators to clinical academic careers. Further exploration and synthesis of this dataset may facilitate a deeper understanding of the barriers and facilitators that clinical academics face and may inform the development of specifically targeted interventions. This systematic review formed part of a larger project focused on UK clinical academic careers, for which linked primary qualitative research was performed. [65] [66] A full and detailed report of the whole project is available online. [67] [68]

For peer review only

Statements

Ethics statement

This study did not receive nor require ethics approval, as it is a systematic review of existing literature.

Funding statement

This systematic review, as well as the larger project it is part of, was funded jointly by the Medical Research Council (MRC), the National Institute for Health Research (NIHR), Wellcome, Health Education England (HEE), the Academy of Medical Sciences, and Cancer Research UK (CRUK) through an award administered by CRUK [C71037/A29824]. The funders identified the focus of the work and the use of systematic review methodology to address the research question. The protocol for and findings of the study were not further influenced by the funders.

Competing Interest Statement

All authors have completed the ICMJE uniform disclosure form (available on request from the corresponding author) and declare: no support from any organisation for the submitted work beyond that described in our funding statement, no financial relationships with any organisations that might have an interest in the submitted work in the previous three years, no other relationships or activities that could appear to have influenced the submitted work.

Data sharing statement

Data included within this review have been extracted from the included studies, in their published form. No additional data is available.

Contributorship statement

JEM, GF, PC and LAS designed the study in collaboration and obtained funding as detailed. Search strategies were developed, tested, and translated by an information specialist with input from JVEB and JEM. JVEB and JEM drafted the protocol and then revised it alongside the other authors. JVEB, JEM, CE, GR, EU, PC, AK and GF screened titles and abstracts for inclusion. JEM, JVEB, CE, GR, and EU screened full texts, performed data extraction, and quality assessment. JEM, CE, GR and EU performed the analyses. All authors were involved in the writing and editing of the manuscript. All authors have read and approved the final manuscript.

Figure legends:

Figure 1: Flowsheet for study selection

References

1. Newington L, Alexander CM, Wells M. Impacts of clinical academic activity: qualitative interviews with healthcare managers and research-active nurses, midwives, allied health professionals and pharmacists. BMJ Open. 2021;11(10):e050679.

2. Boaz A, Hanney S, Jones T, Soper B. Does the engagement of clinicians and organisations in research improve healthcare performance: a three-stage review. BMJ Open. 2015;5(12):e009415.

For peer review only

3. Harding K, Lynch L, Porter J, Taylor NF. Organisational benefits of a strong research culture in a health service: a systematic review. Aust Health Rev. 2017;41(1):45-53.

4. Fitzpatrick S. A survey of staffing levels of medical clinical academics in UK medical schools as at 31 July 2011. London: Medical Schools Council; 2012.

5. Penny M, Jeffries R, Grant J, Davies SC. Women and academic medicine: A review of the evidence on female representation. J R Soc Med. 2014;107(7):259-63.

6. Watson N, Tang P, Knight E. Survey of medical clinical academic staffing levels. London: Medical Schools Council; 2018.

7. Laver KE, Prichard IJ, Cations M, Osenk I, Govin K, Coveney JD. A systematic review of interventions to support the careers of women in academic medicine and other disciplines. BMJ Open. 2018;8(3):e020380.

8. Brown JVE, Crampton PES, Finn GM, Morgan JE, on behalf of the project team. From the sticky floor to the glass ceiling and everything in between: Protocol for a systematic review of barriers and facilitators to clinical academic careers and interventions to address these, with a focus on gender inequality. Syst Rev. 2020;9(1):26.

9. World Bank. World Bank country and lending groups. World Bank Group; 2021 [27th September 2021]; Available from: https://datahelpdesk.worldbank.org/knowledgebase/articles/906519-world-bank-country-and-lending-groups.

10. Ouzzani M, Hammady H, Fedorowicz Z, Elmagarmid A. Rayyan—a web and mobile app for systematic reviews. Syst Rev. 2016;5(1):210.

11. Higgins JPT, Altman DG, Gøtzsche PC, Jüni P, Moher D, Oxman AD, et al. The Cochrane Collaboration's tool for assessing risk of bias in randomised trials. BMJ. 2011;343:d5928.

12. Wells G, Shea B, O'Connell D, Peterson J, Welch, Losos M, et al. The Newcastle-Ottawa Scale (NOS) for assessing the quality of nonrandomised

For peer review only

studies in meta-analyses. 2014 [27th September 2021]; Available from: http://www.ohri.ca/programs/clinical_epidemiology/oxford.asp.

13. Joanna Briggs Institute. Joanna Briggs Institute reviewers' manual: 2014 edition. Australia: The Joanna Briggs Institute; 2014.

14. Hong QN, Fàbregues S, Bartlett G, Boardman F, Cargo M, Dagenais P, et al. The mixed methods appraisal tool (MMAT) version 2018 for information professionals and researchers. Education for Information. 2018;34:285-91.

15. Wong G, Westhorp G, Greenhalgh J, Manzano A, Jagosh J, Greenhalgh T. Quality and reporting standards, resources, training materials and information for realist evaluation: The RAMESES II project. Health Services and Delivery Research. 2017;5.

16. Braun V, Clarke V. Using thematic analysis in psychology. Qual Res Psychol. 2006;3(2):77-101.

17. Healthwatch York. Healthwatch York Homepage. York: Healthwatch York; [27th September 2021]; Available from: https://www.healthwatchyork.co.uk/.

18. Brandt AM, Rettig SA, Kale NK, Zuckerman JD, Egol KA. Can a clinician-scientist training program develop academic orthopaedic surgeons? One program's thirty-year experience. J Surg Educ. 2018;75(4):1039-44.

19. Campion MW, Bhasin RM, Beaudette DJ, Shann MH, Benjamin EJ. Mid-career faculty development in academic medicine: How does it impact faculty and institutional vitality? J Fac Dev. 2016;30(3):49-64.

20. Chang S, Morahan PS, Magrane D, Helitzer D, Lee HY, Newbill S, et al. Retaining faculty in academic medicine: The impact of career development programs for women. J Womens Health (Larchmt). 2016;25(7):687-96.

21. Daley S, Wingard DL, Reznik V. Improving the retention of underrepresented minority faculty in academic medicine. J Natl Med Assoc. 2006;98(9):1435-40.

22. Dannels SA, Yamagata H, McDade SA, Chuang YC, Gleason KA, McLaughlin JM, et al. Evaluating a leadership program: a comparative,

For peer review only

longitudinal study to assess the impact of the Executive Leadership in Academic Medicine (ELAM) Program for Women. Acad Med. 2008;83(5):488-95.

23. Ehlers SL, Cornelius KE, Greenberg-Worisek AJ, Warner DO, Weavers KM, Thomson KR, et al. A matched cohort examination of publication rates among clinical subspecialty fellows enrolled in a translational science training program. J Clin Transl Sci. 2018;2(5):327-33.

24. Emans SJ, Goldberg CT, Milstein ME, Dobriner J. Creating a faculty development office in an academic pediatric hospital: Challenges and successes. Pediatrics. 2008;121(2):390-401.

25. Goldenberg NA, Kruse-Jarres R, Frick N, Pipe SW, Leissinger CA, Kessler CM. Outcomes of mentored, grant-funded fellowship training in haemostasis /thrombosis: Findings from a nested case-control survey study. Haemophilia. 2012;18(3):326-31.

26. Grisso JA, Sammel MD, Rubenstein AH, Speck RM, Conant EF, Scott P, et al. A randomized controlled trial to improve the success of women assistant professors. J Womens Health (Larchmt). 2017;26(5):571-9.

27. Guevara JP, Wright M, Fishman NW, Krol DM, Johnson J. The Harold Amos medical faculty development program: Evaluation of a national program to promote faculty diversity and health equity. Health Equity. 2018;2(1):7-14.

28. Harrison LM, Woods RJ, McCarthy MC, Parikh PP. Development and implementation of a sustainable research curriculum for general surgery residents: A foundation for developing a research culture. Am J Surg. 2020;220(1):105-8.

29. Joshua Smith J, Patel RK, Chen X, Tarpley MJ, Terhune KP. Does intentional support of degree programs in general surgery residency affect research productivity or pursuit of academic surgery? J Surg Educ. 2014;71(4):486-91.

For peer review only

30. Khot S, Park BS, Longstreth WT, Jr. The Vietnam war and medical research: untold legacy of the U.S. doctor draft and the NIH "Yellow Berets". Acad Med. 2011;86(4):502-8.

31. Kohlwes RJ, Shunk RL, Avins A, Garber J, Bent S, Shlipak MG. The PRIME curriculum. Clinical research training during residency. J Gen Intern Med. 2006;21(5):506-9.

32. Kohlwes J, O'Brien B, Stanley M, Grant R, Shunk R, Connor D, et al. Does research training during residency promote scholarship and influence career choice? A cross-sectional analysis of a 10-year cohort of the UCSF-PRIME internal medicine residency program. Teach Learn Med. 2016;28(3):314-9.

33. Libby AM, Hosokawa PW, Fairclough DL, Prochazka AV, Jones PJ, Ginde AA. Grant success for early-career faculty in patient-oriented research: Difference-in-differences evaluation of an interdisciplinary mentored research training program. Acad Med. 2016;91(12):1666-75.

34. Mandel BA, Weber SM, Gutowski KA, Salyapongse AN, Bentz ML. What influences a plastic surgery resident to pursue an academic career? Plast Reconstr Surg Glob Open. 2018;6(10):e1860.

35. Mills LS, Steiner AZ, Rodman AM, Donnell CL, Steiner MJ. Trainee participation in an annual research day is associated with future publications. Teach Learn Med. 2011;23(1):62-7.

36. Nasab S, Rushing JS, Segars JH, Evers E, Handa VL, Lawson S, et al. A mentorship program for academic obstetrician gynecologists that improved publication and overall confidence for success. Semin Reprod Med. 2019;37(5-06):257-64.

37. Ockene JK, Milner RJ, Thorndyke LE, Congdon J, Cain JM. Peers for promotion: Achieving academic advancement through facilitated peer mentoring. J Fac Dev. 2017;31(3):5-13.

For peer review only

38. Patel MS, Tomich D, Kent TS, Chaikof EL, Rodrigue JR. A program for promoting clinical scholarship in general surgery. J Surg Educ. 2018;75(4):854-60.
39. Ries A, Wingard D, Morgan C, Farrell E, Letter S, Reznik V. Retention of junior faculty in academic medicine at the University of California, San Diego. Acad Med. 2009;84(1):37-41.
40. Ries A, Wingard D, Gamst A, Larsen C, Farrell E, Reznik V. Measuring faculty retention and success in academic medicine. Acad Med. 2012;87(8):1046-51.
41. Sheridan JT, Fine E, Pribbenow CM, Handelsman J, Carnes M. Searching for excellence & diversity: Increasing the hiring of women faculty at one academic medical center. Acad Med. 2010;85(6):999-1007.
42. Valantine HA, Grewal D, Ku MC, Moseley J, Shih MC, Stevenson D, et al. The gender gap in academic medicine: comparing results from a multifaceted intervention for stanford faculty to peer and national cohorts. Acad Med. 2014;89(6):904-11.
43. Winn AS, Emans SJ, Newman LR, Sandora TJ. Promoting resident professional development using scholarly academies. Acad Pediatr. 2018;18(4):477-9.
44. Klimas J, Fernandes E, deBeck K, Hayashi K, Milloy MJ, Kerr T, et al. Preliminary results and publication impact of a dedicated addiction clinician scientist research fellowship. J Addict Med. 2017;11(1):80-1.
45. Merani S, Switzer N, Kayssi A, Blitz M, Ahmed N, Shapiro AM. Research productivity of residents and surgeons with formal research training. J Surg Educ. 2014;71(6):865-70.
46. Sweeny A, van den Berg L, Hocking J, Renaud J, Young S, Henshaw R, et al. A Queensland research support network in emergency healthcare. J Health Organ Manag. 2019;33(1):93-109.

For peer review only

47. Löwe B, Hartmann M, Wild B, Nikendei C, Kroenke K, Niehoff D, et al. Effectiveness of a 1-year resident training program in clinical research: A controlled before-and-after study. J Gen Intern Med. 2008;23(2):122-8.
48. Comeau DL, Escoffery C, Freedman A, Ziegler TR, Blumberg HM. Improving clinical and translational research training: A qualitative evaluation of the Atlanta Clinical and Translational Science Institute KL2-mentored research scholars program. J Investig Med. 2017;65(1):23-31.
49. DeCastro R, Sambuco D, Ubel PA, Stewart A, Jagsi R. Mentor networks in academic medicine: moving beyond a dyadic conception of mentoring for junior faculty researchers. Acad Med. 2013a;88(4):488-96.
50. DeCastro R, Sambuco D, Ubel PA, Stewart A, Jagsi R. Batting 300 is good: perspectives of faculty researchers and their mentors on rejection, resilience, and persistence in academic medical careers. Acad Med. 2013b;88(4):497-504.
51. Helitzer DL, Newbill SL, Cardinali G, Morahan PS, Chang S, Magrane D. Narratives of participants in national career development programs for women in academic medicine: identifying the opportunities for strategic investment. J Womens Health (Larchmt). 2016;25(4):360-70.
52. Jones RD, Miller J, Vitous CA, Krenz C, Brady KT, Brown AJ, et al. The most valuable resource is time: Insights from a novel national program to Improve retention of physician-scientists with caregiving responsibilities. Acad Med. 2019;94(11):1746-56.
53. Kraemer RR, Wakelee JF, Hites L, Frank SJ, Saag K, Rogers DA, et al. Moving career development upstream: Evaluation of a course for internal medicine trainees contemplating career pathways in academic medicine. South Med J. 2018;111(8):471-5.
54. Lin MP, Lall MD, Samuels-Kalow M, Das D, Linden JA, Perman S, et al. Impact of a women-focused professional organization on academic retention and advancement: Perceptions from a qualitative study. Acad Emerg Med. 2019;26(3):303-16.

For peer review only

55. Reader S, Fornari A, Simon S, Townsend J. Promoting faculty scholarship - An evaluation of a program for busy clinician-educators. Can Med Educ J. 2015;6(1):e43-60.
56. Stubbe D, Martin A, Bloch M, Belitsky R, Carter D, Ebert M, et al. Model curriculum for academic child and adolescent psychiatry training. Acad Psychiatry. 2008;32(5):366-76.
57. Archibald D, Hogg W, Lemelin J, Dahrouge S, St Jean M, Boucher F. Building capacity for medical education research in family medicine: The Program for Innovation in Medical Education (PIME). Health Res Policy Syst. 2017;15(1):91.
58. Hayward CP, Danoff D, Kennedy M, Lee AC, Brzezina S, Bond U. Clinician investigator training in Canada: a review. Clin Invest Med. 2011;34(4):E192-E201.
59. Klimas J, McNeil R, Ahamad K, Mead A, Rieb L, Cullen W, et al. Two birds with one stone: experiences of combining clinical and research training in addiction medicine. BMC Med Educ. 2017a;17(1):22.
60. Klimas J, Small W, Ahamad K, Cullen W, Mead A, Rieb L, et al. Barriers and facilitators to implementing addiction medicine fellowships: A qualitative study with fellows, medical students, residents and preceptors. Addict Sci Clin Pract. 2017b;12(1):21.
61. Moss J, Teshima J, Leszcz M. Peer group mentoring of junior faculty. Acad Psychiatry. 2008;32(3):230-5.
62. Caffrey L, Wyatt D, Fudge N, Mattingley H, Williamson C, McKevitt C. Gender equity programmes in academic medicine: a realist evaluation approach to Athena SWAN processes. BMJ Open. 2016;6(9):e012090.
63. Darbyshire D, Baker P, Agius S, McAleer S. Trainee and supervisor experience of the academic foundation programme. J R Coll Physicians Edinb. 2019;49(1):43-51.

For peer review only

64. Iversen AC, Eady NA, Wessely SC. The role of mentoring in academic career progression: A cross-sectional survey of the academy of medical sciences mentoring scheme. J R Soc Med. 2014;107(8):308-17.

65. Finn GM, Crampton P, Buchanan JAG, Balogun AO, Tiffin PA, Morgan JE, et al. The impact of the COVID-19 pandemic on the research activity and working experience of clinical academics, with a focus on gender and ethnicity: a qualitative study in the UK. BMJ Open. 2022;12(6):e057655.

66. Kehoe A, Crampton P, Buchanan J, Tiffin P, Balogun-Katung A, Morgan J, et al. Tips to support the recruitment, retention, and progression of clinical academics. Medical Science Educator. 2022;32(2):503-9.

67. Finn G, Brown J, Evans C, Raine G, Stewart L, Uphoff E, et al. From the sticky floor to the glass ceiling and everything in between: A systematic review and qualitative study focusing on gender inequalities in Clinical Academic careers. Final Report. Commissioned by: NIHR Academy, Academy of Medical Sciences, Cancer Research UK, Health Education England, Medical Research Council, and Wellcome Trust. 2020 [27th September 2021]; Available from: https://www.hyms.ac.uk/research/research-centres-and-groups/hpeu/gender-inequalities-in-clinical-academic-careers.

68. Finn G, Brown J, Evans C, Raine G, Stewart L, Uphoff E, et al. Inequalities in UK clinical academic careers: a systematic review and qualitative study. Short Report. Commissioned by: NIHR Academy, Academy of Medical Sciences, Cancer Research UK, Health Education England, Medical Research Council, and Wellcome Trust. 2021 [27th September 2021]; Available from: https://www.hyms.ac.uk/research/research-centres-and-groups/hpeu/gender-inequalities-in-clinical-academic-careers.

For peer review only

For peer review only

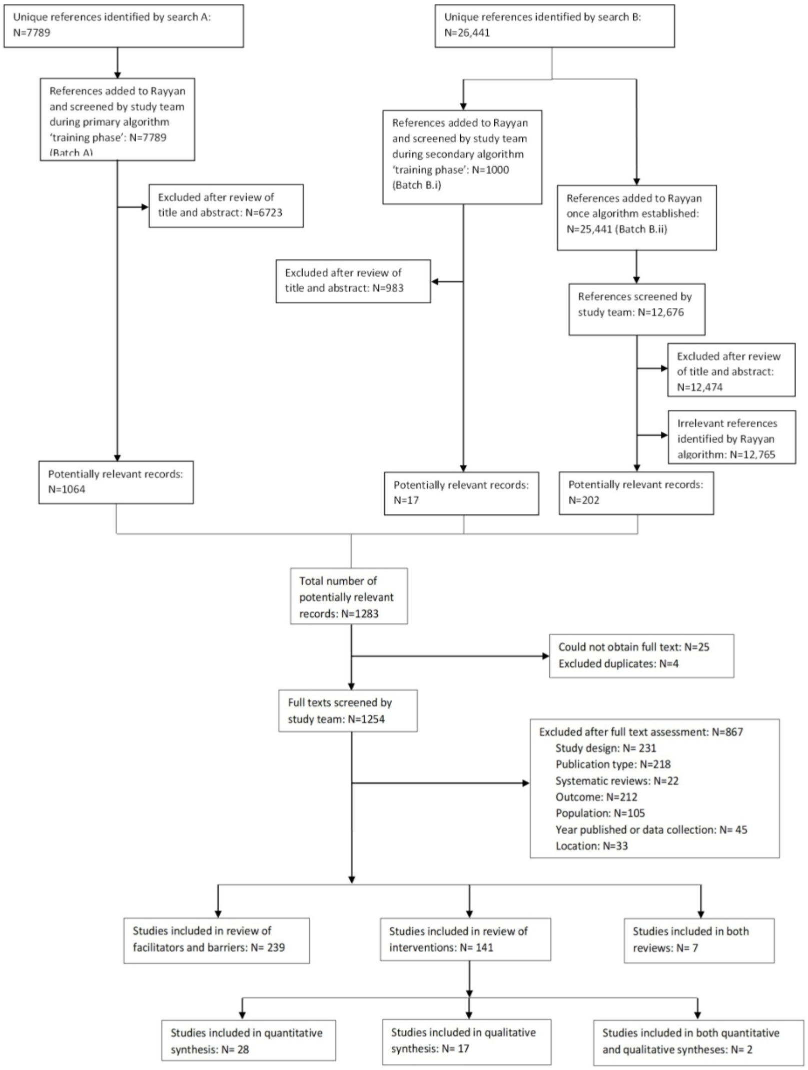

Flowsheet for study selection

153x194mm (330 x 330 DPI)

Appendix 1: Medline search strategy

MEDLINE(R) ALL

via Ovid http://ovidsp.ovid.com/
1946 to October 25, 2019
Searched on: 28th October 2019
Records retrieved search B: 13473
Records retrieved search A: 4241

1 ((doctor or doctors or physician$ or medic or medics) adj4 academi$).ti,ab. (1872)
2 ((doctor or doctors or physician$ or medic or medics) adj4 (professor$ or dean$ or program$ director$ or lecturer$ or research fellow$ or researcher$)).ti,ab. (1979)
3 ((doctor or doctors or physician$ or medic or medics) adj4 (doctora$ or predoctora$ or pre-doctora$ or postdoctora$ or post-doctora$ or postdoc or post-doc or postdocs or post-docs or PhD or PhDs)).ti,ab. (232)
4 ((doctor or doctors or physician$ or medic or medics) adj4 (universit$ or higher education or research institut$ or research centre$ or research center$)).ti,ab. (1435)
5 (medical adj (profession$ or practitioner$ or specialist$) adj4 academi$).ti,ab. (65)
6 (medical adj (profession$ or practitioner$ or specialist$) adj4 (professor$ or dean$ or program$ director$ or lecturer$ or research fellow$ or researcher$)).ti,ab. (154)
7 (medical adj (profession$ or practitioner$ or specialist$) adj4 (doctora$ or predoctora$ or pre-doctora$ or postdoctora$ or post-doctora$ or postdoc or post-doc or postdocs or post-docs or PhD or PhDs)).ti,ab. (5)
8 (medical adj (profession$ or practitioner$ or specialist$) adj4 (universit$ or higher education or research institut$ or research centre$ or research center$)).ti,ab. (59)
9 ((GP or GPs or general practioner$) adj4 academi$).ti,ab. (75)
10 ((GP or GPs or general practioner$) adj4 (professor$ or dean$ or program$ director$ or lecturer$ or research fellow$ or researcher$)).ti,ab. (96)
11 ((GP or GPs or general practioner$) adj4 (doctora$ or predoctora$ or pre-doctora$ or postdoctora$ or post-doctora$ or postdoc or post-doc or postdocs or post-docs or PhD or PhDs)).ti,ab. (4)
12 ((GP or GPs or general practioner$) adj4 (universit$ or higher education or research institut$ or research centre$ or research center$)).ti,ab. (59)
13 ((dentist or dentists) adj4 academi$).ti,ab. (63)
14 ((dentist or dentists) adj4 (professor$ or dean$ or program$ director$ or lecturer$ or research fellow$ or researcher$)).ti,ab. (73)
15 ((dentist or dentists) adj4 (doctora$ or predoctora$ or pre-doctora$ or postdoctora$ or post-doctora$ or postdoc or post-doc or postdocs or post-docs or PhD or PhDs)).ti,ab. (20)
16 ((dentist or dentists) adj4 (universit$ or higher education or research institut$ or research centre$ or research center$)).ti,ab. (72)
17 ((dental or dentistry) adj (profession$ or practitioner$ or specialist$) adj4 academi$).ti,ab. (24)
18 ((dental or dentistry) adj (profession$ or practitioner$ or specialist$) adj4 (professor$ or dean$ or program$ director$ or lecturer$ or research fellow$ or researcher$)).ti,ab. (22)
19 ((dental or dentistry) adj (profession$ or practitioner$ or specialist$) adj4 (doctora$ or predoctora$ or pre-doctora$ or postdoctora$ or post-doctora$ or postdoc or post-doc or postdocs or post-docs or PhD or PhDs)).ti,ab. (1)
20 ((dental or dentistry) adj (profession$ or practitioner$ or specialist$) adj4 (universit$ or higher education or research institut$ or research centre$ or research center$)).ti,ab. (23)
21 or/1-20 (6146)
22 ((consultant$ or registrar$ or associate specialist$ or staff grade$ or house officer$ or houseman or housemen or housestaff) adj4 academi$).ti,ab. (139)
23 ((consultant$ or registrar$ or associate specialist$ or staff grade$ or house officer$ or houseman or housemen or housestaff) adj4 (professor$ or dean$ or program$ director$ or lecturer$ or research fellow$ or researcher$)).ti,ab. (242)

24 ((consultant$ or registrar$ or associate specialist$ or staff grade$ or house officer$ or houseman or housemen or housestaff) adj4 (doctora$ or predoctora$ or pre-doctora$ or postdoctora$ or post-doctora$ or postdoc or post-doc or postdocs or post-docs or PhD or PhDs)).ti,ab. (18)
25 ((consultant$ or registrar$ or associate specialist$ or staff grade$ or house officer$ or houseman or housemen or housestaff) adj4 (universit$ or higher education or research institut$ or research centre$ or research center$)).ti,ab. (166)
26 ((medical or specialt$ or specialist$ or clinical or surgical) adj4 train$ adj4 academi$).ti,ab. (445)
27 ((medical or specialt$ or specialist$ or clinical or surgical) adj4 train$ adj4 (professor$ or dean$ or program$ director$ or lecturer$ or research fellow$ or researcher$)).ti,ab. (188)
28 ((medical or specialt$ or specialist$ or clinical or surgical) adj4 train$ adj4 (doctora$ or predoctora$ or pre-doctora$ or postdoctora$ or post-doctora$ or postdoc or post-doc or postdocs or post-docs or PhD or PhDs)).ti,ab. (116)
29 ((medical or specialt$ or specialist$ or clinical or surgical) adj4 train$ adj4 (universit$ or higher education or research institut$ or research centre$ or research center$)).ti,ab. (468)
30 ((FY1 or FY2 or SHO or JHO or FY train$ or CMT or CST) adj10 academi$).ti,ab. (4)
31 ((FY1 or FY2 or SHO or JHO or FY train$ or CMT or CST) adj10 (professor$ or dean$ or program$ director$ or lecturer$ or research fellow$ or researcher$)).ti,ab. (20)
32 ((FY1 or FY2 or SHO or JHO or FY train$ or CMT or CST) adj10 (doctora$ or predoctora$ or pre-doctora$ or postdoctora$ or post-doctora$ or postdoc or post-doc or postdocs or post-docs or PhD or PhDs)).ti,ab. (0)
33 ((FY1 or FY2 or SHO or JHO or FY train$ or CMT or CST) adj10 (universit$ or higher education or research institut$ or research centre$ or research center$)).ti,ab. (31)
34 or/22-33 (1774)
35 (facult$ adj5 (medical or medicine or dental or dentistry or clinical) adj5 (academi$ or research$ or scholar$)).ti,ab. (1313)
36 (facult$ adj5 (medical or medicine or dental or dentistry or clinical) adj5 (professor$ or dean$ or program$ director$ or lecturer$ or research fellow$ or researcher$)).ti,ab. (399)
37 (facult$ adj5 (medical or medicine or dental or dentistry or clinical) adj5 (doctora$ or predoctora$ or pre-doctora$ or postdoctora$ or post-doctora$ or postdoc or post-doc or postdocs or post-docs or PhD or PhDs)).ti,ab. (79)
38 or/35-37 (1705)
39 exp Physicians/ (133681)
40 exp Dentists/ (18486)
41 Faculty, Medical/ (12770)
42 Faculty, Dental/ (2398)
43 Academic Medical Centers/ (17857)
44 39 or 40 or 41 or 42 or 43 (180811)
45 Research Personnel/ (15675)
46 Universities/ (38946)
47 research/ or biomedical research/ or dental research/ (267864)
48 45 or 46 or 47 (313057)
49 44 and 48 (7101)
50 21 or 34 or 38 or 49 (15969)
51 (academic adj (medicine or dentistry or primary care)).ti,ab. (2568)
52 (academic adj2 (an?esthesi$ or an?estheti$ or oncolog$ or emergency medicine or radiolog$ or intensive care or intensivist$ or obstetric$ or gyn?ecolog$ or ophthalmolog$ or paediatric$ or pediatric$ or patholog$ or psychiatr$ or public health or surgery or surgeon$)).ti,ab. (4937)
53 51 or 52 (7395)
54 ((clinical or clinician$ or medical or dental or dentistry) adj academi$).ti,ab. (780)
55 ((clinical or clinician$ or medical or dental or dentistry) adj (lecturer$ or lectureship$)).ti,ab. (61)
56 ((clinical or clinician$ or medical or dental or dentistry) adj professor$).ti,ab. (177)

57 ((clinical or clinician$ or medical or dental or dentistry) adj fellow$).ti,ab. (325)
58 ((clinical or clinician$ or medical or dental or dentistry) adj research fellow$).ti,ab. (37)
59 in-practice fellow$.ti,ab. (8)
60 clinical research train$.ti,ab. (103)
61 physician$ scientist$.ti,ab. (854)
62 surgeon$ scientist$.ti,ab. (165)
63 ((clinical or clinician$) adj scientist$).ti,ab. (1193)
64 ((clinical or clinician$) adj scholar$).ti,ab. (175)
65 ((clinical or clinician$) adj researcher$).ti,ab. (2597)
66 ((clinical or clinician$) adj investigator$).ti,ab. (1649)
67 ((clinical or clinician$) adj educator$).ti,ab. (1011)
68 or/54-67 (8891)
69 50 or 53 or 68 (30102)
70 (integrated adj3 academic adj3 (train$ or career$ or path or paths or pathway$ or program$)).ti,ab. (33)
71 (IAT adj2 (career$ or path$ or program$)).ti,ab. (10)
72 Clinical Research Training Fellowship$.ti,ab. (10)
73 Academic Foundation Program$.ti,ab. (17)
74 (academi$ adj3 (clinical or clinician$ or medical or medicine or dental or dentistry) adj3 (career$ or path or paths or pathway$)).ti,ab. (383)
75 (research$ adj3 (clinical or clinician$ or medical or medicine or dental or dentistry) adj3 (career$ or path or paths or pathway$)).ti,ab. (330)
76 or/70-75 (751)
77 Career Choice/ (22485)
78 career mobility/ (11228)
79 Staff Development/ (9142)
80 (career$ or pathway$ or pipeline$).ti,ab. (1105747)
81 ((occupation$ or profession$ or job$ or staff or employee$ or personnel) adj3 (choice$ or choos$ or select$ or decid$ or decision$)).ti,ab. (7714)
82 ((occupation$ or profession$ or job$ or staff or employee$ or personnel) adj3 (mobility or ladder$ or route$ or trajector$ or structure$)).ti,ab. (2939)
83 ((occupation$ or profession$ or job$ or staff or employee$ or personnel) adj3 (progress$ or promot$ or develop$ or advanc$)).ti,ab. (26577)
84 or/77-83 (1166195)
85 69 and 84 (4314)
86 Personnel Selection/ (12625)
87 (recruit$ or hire$ or hiring).ti,ab. (355306)
88 86 or 87 (363489)
89 69 and 88 (1460)
90 Personnel Turnover/ (5027)
91 (retain$ or retention).ti,ab. (350726)
92 (resign$ or terminat$ or disenroll$ or withdraw$ or attrition).ti,ab. (247203)
93 90 or 91 or 92 (595449)
94 69 and 93 (887)
95 85 or 89 or 94 (5724)
96 76 or 95 (6051)
97 exp animals/ not humans/ (4637358)
98 96 not 97 (6040)
99 limit 98 to english language (5741)
100 limit 99 to yr="2004 -Current" (4471)
101 (editorial or letter).pt. (1554107)
102 100 not 101 (4241) **[Records downloaded for search A – clinical academics AND career]**
103 69 or 76 (30413)
104 103 not 97 (30248)

105 limit 104 to english language (27498)
106 limit 105 to yr="2004 -Current" (18930)
107 106 not 101 (17714)
108 107 not 102 (13473) **[Records downloaded for search B – clinical academics with search A results removed]**

For peer review only

Appendix 2: Table of data for studies included in synthesis

First author (Year of publication; country)	Years of data collection	Study design	Grade/type of clinical academics included	Number of participants (Male: Female)	Ethnicity	Intervention components	Target population of intervention	Aimed at particular group	Reach
Studies included within quantitative analysis									
Brandt (2018; USA)[19]	NR- NR	Cohort study. Concurrent & historical control	Orthopaedic surgery residents, academic level NR	329 (NR)	NR	Mentorship programme - junior mentee/senior mentor, Protected research time	Trainees	No	Single centre
Chang (2016; USA)[21]	1988-2009	Cohort study. Concurrent control	Clinically mixed group, post-training, range of academic levels	65099, of which 3268 in intervention group	NR	Leadership training. Faculty career development program, not described in detail for individual programs. EWIM & MWIM programs focus on academic skills development; ELAM is leadership training	Junior Faculty, Managers/ leaders/ senior faculty	Gender	National
Daley (2006; USA)[22]	NR- 2005	Cohort study. Concurrent & historical control	Mixed group of faculty (medical, surgical, psychiatry, GP), mixed academic levels	112 (48:64)	15 URM	Mentorship programme - junior mentee/ senior mentor. Teaching sessions/ lectures, leadership training, faculty career development programme, networking/ collaboration/ social activities, academic performance counselling	Junior Faculty	Ethnicity, URM	Single centre
Dannels (2008; USA)[23]	2002-2006	Cohort study. Concurrent control	Post-training, post-doctoral. Speciality NR	250 (0:250)	NR	Leadership training	Managers/ leaders/ senior faculty	Gender	Single centre
Ehlers (2018; USA)[24]	2018-2018	Cohort study. Concurrent control	Pre-doctoral fellows in cardiovascular diseases, gastroenterology and hepatology/ oncology	140 (76:64)	NR	Mentorship programme - junior mentee/ senior mentor. Teaching sessions/ lectures, protected research time. Teaching is focused on research training. E.g. research methods, protocol development, grant writing and manuscript writing, statistics and epidemiology. Also complete a mentored research project	Trainees, Junior Faculty	No	Single centre
Emans (2008; USA)[25]	2006- NR	Cohort study. Historical control	Academic paediatric faculty. Academic level NR	238 (54%:46%)	NR	Mentorship programme - junior mentee/ senior mentor. Teaching sessions/ lectures, leadership training, networking/ collaboration/ social activities, 'Community	Junior Faculty	Gender, Ethnicity	Single centre

For peer review only

First author (Year of publication; country)	Years of data collection	Study design	Grade/type of clinical academics included	Number of participants (Male: Female)	Ethnicity	Intervention components	Target population of intervention	Aimed at particular group	Reach
						of mentors'. Work-life balance, cultural competency and diversity initiatives			
Goldenberg (2012; USA)[26]	NR- NR	Case control. Concurrent control	Trainees in academic haematology/ oncology. Academic level NR	20 (10 females)	NR	Mentorship programme - junior mentee/ senior mentor	Trainees	No	National
Grisso (2017; USA)[27]	2010- NR	Multifaceted cluster randomised intervention trial	Assistant professors, speciality NR	132 (0:132)	7.6% African American, 60.3% White, 27.5% Asian, 4.6% Hispanic/ other	Mentorship programme - junior mentee/ senior mentor. Mentorship programme - peers, grant writing programme, teaching sessions/ lectures, networking/ collaboration/ social activities, manuscript writing programme, also included a "Total Leadership Programme" which address work-life integration/ balance	Junior Faculty	Gender	Single centre
Harrison (2020; USA)[29]	2007- 2017	Cohort study. Historical control	General surgery residents, academic level NR	203 (NR)	NR	Mentorship programme - junior mentee/ senior mentor. Teaching sessions/ lectures, annual milestones, research meetings and feedback on completing research projects	Trainees	No	Single centre
Joshua Smith (2014; USA)[30]	NR-NR	Cohort study. Concurrent control	General surgery trainees. Academic level NR	68 (NR)	NR	Research training; Protected research time	Trainees	No	Single centre
Khot (2011; USA)[31]	2007- 2007	Cohort study. Concurrent control	Post-training, post-doctoral. Speciality not reported	1577 NIH associates (4 female) who entered academic medicine; 27821 non-associates	NR	Teaching sessions/ lectures, protected research time. Little detail reported	Trainees	No	National
Klimas (2017; Canada)[45]	2014- 2014	Cohort study. Concurrent control	Trainees in internal medicine, family medicine, public health,	8 (3:5)	NR	Mentorship programme - junior mentee/ senior mentor. Teaching sessions/ lectures	Trainees	No	National

First author (Year of publication; country)	Years of data collection	Study design	Grade/type of clinical academics included	Number of participants (Male: Female)	Ethnicity	Intervention components	Target population of intervention	Aimed at particular group	Reach
			psychiatry, mix of academic levels						
Kohlwes (2006; USA)[32]	2001-2004	Cohort study. Concurrent control	Trainees in internal medicine. Academic level NR	32 (NR)	NR	Mentorship programme - junior mentee/senior mentor. Teaching sessions/ lectures, protected research time, small group journal clubs; work in progress sessions; conduct a clinical research project	Trainees	No	Single Centre
Kohlwes 2016; USA)[33]	NR- NR	Cohort study. Concurrent control	Post-training in internal medicine. Academic level NR	169 (88:81)	88 white, 1 black, 31 Asian Indian, 28 Asian Other, 12 Hispanic, 6 Other	Mentorship programme - junior mentee/ senior mentor. Teaching sessions/ lectures, protected research time, participants conduct research projects. Salary support provided for PRIME director and associate director	Trainees	No	Single Centre
Libby (2016; USA)[34]	2000-2011	Cohort study. Concurrent control	Faculty from many disciplines of medicine/surgery as well as disciplines outside medicine. Post-doctoral	25 CFSP scholars; 125 comparison faculty (NR)	NR	Mentorship programme - junior mentee/ senior mentor. Mentorship programme - peers, teaching sessions/ lectures, protected research time	Junior Faculty	No	Single centre
Löwe (2008; Western Europe)[48]	2005-2006	Cohort study. Concurrent control	Trainees from Internal Medicine, psychotherapy, psychosomatics, psychiatry, psychology. Academic level NR	44 (17:27)	NR	Mentorship programme - junior mentee/ senior mentor. Teaching sessions/ lectures, teaching sessions held as part of a 'clinical research methods' course. Participants also conducted an individual research project	Trainees	No	Single centre
Mandel (2018; USA)[35]	NR- NR	Cohort study. Historical control	Post-training plastic surgeons, academic level NR	28 (24:4)	NR	Teaching sessions/lectures, protected research time. 1-2 year research fellowship, senior mentorship	Trainees	No	Single centre
Merani (2014; Canada)[46]	1988-2012	Cohort study. Concurrent control	General surgery residents, mixed pre-doctoral and doctoral	323 (NR)	NR	Research training	Trainees	No	National

For peer review only - http://bmjopen.bmj.com/site/about/guidelines.xhtml

First author (Year of publication; country)	Years of data collection	Study design	Grade/type of clinical academics included	Number of participants (Male: Female)	Ethnicity	Intervention components	Target population of intervention	Aimed at particular group	Reach
Mills (2011; USA)[36]	Post 2007-NR	Cohort study. Concurrent control	Paediatric residents and fellows, academic level NR	526 (197:297)	NR	Departmental research day at which paediatrics trainees present submitted research and other scholarly work	Trainees	No	Single centre
Nasab (2019; USA)[37]	2018-2018	Cohort study. Concurrent control	Obstetricians & Gynecologists, trainees and post-training, range of academic levels	109 attended, 94 pre-course survey, 74 post course survey (NR)	63% white, 9% black, 5% Asian, 3% other, 20% did not disclose	Mentorship programme - junior mentee/ senior mentor. Teaching sessions/lectures, leadership training, networking/ collaboration/ social activities. Particular sessions included mock interviews, CV reviews, and grant writing support. 60-minute interactive sessions. Participants also given the opportunity to submit their grant proposals	Trainees, Junior Faculty	No	Single Centre
Ockene (2017; USA)[38]	NR- NR	Cohort study. Concurrent control	Post-doctoral, post-training, primary care physicians	32 (11:21)	NR	Mentorship programme - junior mentee/ senior mentor. Mentorship programme – peers. Teaching sessions/ lectures, networking/ collaboration/ social activities	Junior Faculty	No	Single centre
Patel (2018; USA)[39]	2011-2016	Cohort study. Historical control	General surgery residents and faculty mentors. Mentors post-doctoral	67 (NR)	NR	Mentorship programme - junior mentee/ senior mentor. Teaching sessions/ lectures, protected research time, completion of a defined clinical research project. Other didactic activities included: active participation in the department's weekly clinical research meeting; attend lab meetings with faculty research mentor. Webinars on research and grant writing	Trainees	No	Single centre
Ries (2009; USA)[40]	1988-2006	Cohort study. Concurrent control	Assistant professors in school of medicine. Speciality NR	839 (520-319)	52 URM	Mentorship programme - junior mentee/ senior mentor. Networking/ collaboration/ social activities, professional development workshops, performance counselling sessions	Junior Faculty	No	Single centre
Ries (2012; USA)[41]	Post 2005-2006	Case control. Concurrent control	Assistant professors from mixed specialities	315 (152:163)	26 URM	Mentorship programme - junior mentee/ senior mentor. Networking/ collaboration/ social activities, faculty career development programme, Performance counselling sessions	Junior Faculty	No	Single centre

For peer review only

First author (Year of publication; country)	Years of data collection	Study design	Grade/type of clinical academics included	Number of participants (Male: Female)	Ethnicity	Intervention components	Target population of intervention	Aimed at particular group	Reach
Sheridan (2010; USA)[42]	2000-2008	Cohort study. Concurrent control	Post-training. Speciality NR. Academic level NR	163 (NR)	NR	Recruitment training (e.g. to reduce bias in interviews)	Junior Faculty, Managers/ leaders/ senior faculty	Gender, Ethnicity, "Individuals from any group that has been historically under-represented on the faculties of academic health centres"	Single centre
Sweeny (2019; Australia/New Zealand)[47]	2015-2018	Cohort study. Historical control	Emergency Medicine, grade and academic level NR	33 Emergency departments (NR)	NR	Networking/ collaboration/ social activities, research support	Clinical researchers in hospital departments	No	National
Valantine (2014; USA)[43]	2011-2011	Cohort study. Concurrent control	Post-training, post-doctoral, speciality NR	Varying number of participants over several time periods	NR	Mentorship programme - junior mentee/ senior mentor. Funding award, teaching sessions/ lectures, leadership training, protected research time, networking/ collaboration/ social activities, McCormick faculty awards. Faculty Fellows leadership program - individualised career development planning, skill building workshops, and the Women's faculty networking program. Intervention involved a series of programmes	Junior Faculty, Managers/ leaders/ senior faculty	Gender	Single centre
Winn (2018; USA)[44]	2013-2016	Cohort study, case control. Historical control	Internal medicine and paediatrics trainees, academic level NR	48 (NR)	NR	Mentorship programme - junior mentee/senior mentor. Mentorship programme – peers. Teaching sessions/lectures, protected research time, "scholarly homes"	Trainees	No	Single centre
Studies included within qualitative analysis									

For peer review only

First author (Year of publication; country)	Years of data collection	Study design	Grade/type of clinical academics included	Number of participants (Male: Female)	Ethnicity	Intervention components	Target population of intervention	Aimed at particular group	Reach
Archibald (2017; Canada)[58]	2013-2013	Qualitative	Physicians in family medicine, academic level NR	14 (NR)	NR	Mentorship programme - junior mentee/ senior mentor. Funding award, protected research time. Assistants and coordinators provide resources and research support, networking/ collaboration/ social activities	All faculty staff	No	Single centre
Caffrey (2016; UK)[63]	2015-2015	Qualitative	Medical school staff, grade NR, mixed academic levels	31 (NR)	NR	Athena SWAN programme	Trainees, Junior Faculty, Managers/ leaders/ senior faculty, Relevant to whole of department	Gender	National
Comeau (2017; USA)[49]	2007-2014	Mixed methods. Quantitative work had no control	Trainees and post-training, mix of specialities, mix of academic levels	46 (16:30)	22 White, 24 Minority	Mentorship programme - junior mentee/ senior mentor. Funding award, teaching sessions/ lectures, protected research time	Junior Faculty	No	National
Darbyshire (2019; UK)[64]	2014-2014	Mixed methods. Quantitative work had no control	AFP Trainees (survey) and post-training (interviews), mix of specialities, pre-doctoral	34 survey; 7 interviews (NR)	NR	Research training programme; protected research time	Trainees	No	National
*DeCastro (2013a; USA)[50]	2010-2011	Qualitative	Post-training, mix of specialities, mixed academic levels	128 (52:76)	99 White, 7 Black, 3 Hispanic, 18 Asian	Mentorship programme - junior mentee/senior mentor. Protected research time, funding award	Junior Faculty	No	National
*DeCastro (2013b; USA)[51]	2010-2011	Qualitative	Post-training, mix of specialities, mixed academic levels	128 (52:76)	99 White, 7 Black, 3 Hispanic, 18 Asian	Mentorship programme - junior mentee/senior mentor. Protected research time, funding award	Junior Faculty	No	National

First author (Year of publication; country)	Years of data collection	Study design	Grade/type of clinical academics included	Number of participants (Male: Female)	Ethnicity	Intervention components	Target population of intervention	Aimed at particular group	Reach
Hayward (2011; Canada)[59]	2008-2008	Mixed methods. Quantitative work had no control	Trainees and post-training, mix of specialities, mix of academic levels	211 (survey); 13 (qualitative) (NR)	NR	Teaching sessions/ lectures, protected research time, mentorship programme - junior mentees/ senior mentors	Trainees	No	National
Helitzer (2016; USA)[52]	2011- NR	Qualitative (Part of a larger mixed methods study but only qualitative findings reported in this paper)	Post-training, mix of specialities, mixed academic levels	45 (0:45)	NR	Not described in detail for the three programmes separately. EWIM & MWIM held over three days and involved skills and confidence building. ELAM programme was leadership training. All three are faculty career development programmes	Junior Faculty, Managers/ leaders/ senior faculty	Gender	National
Iversen (2014; UK)[65]	2010-2010	Mixed methods. Quantitative work had no control	Post-training, mix of specialities, academic level unclear	147 mentees (67% male) & 77 mentors (81% male)	NR	Mentorship programme - junior mentee/ senior mentor	Junior Faculty	No	National
Jones (2019; USA)[53]	2018-2018	Qualitative study	Early-career or junior physician-scientists, range of specialities, academic level NR	28 (5:23)	11 Non-Hispanic white; 7 Asian; 7 Other; 3 NR	Funding award. Some institutions have also provided access to other forms of support, such as leadership development training, training in academic and career development skills, and/or networking.	Junior Faculty	No, but 85% of awardees are women and tailored more towards those with extra-professional caregiving demands	National
*Klimas (2017a; Canada)[60]	2015-2015	Qualitative	Fellowship in addiction medicine. Trainees from Psychiatry,	26 (12:14)	NR	Mentorship programme - junior mentee/ senior mentor. Funding award, teaching sessions/ lectures, specialty training	Trainees	No	Single centre

For peer review

First author (Year of publication; country)	Years of data collection	Study design	Grade/type of clinical academics included	Number of participants (Male: Female)	Ethnicity	Intervention components	Target population of intervention	Aimed at particular group	Reach
			Internal Medicine, Family Medicine, Nursing & Social Work. Academic level NR						
*Klimas (2017b; Canada)[61]	2015-2015	Qualitative	Fellowship in addiction medicine. Trainees from Psychiatry, Internal Medicine, Family Medicine, Nursing & Social Work. Academic level NR	26 (12:14)	NR	Mentorship programme - junior mentee/ senior mentor. Funding award, teaching sessions/ lectures, specialty training	Trainees	No	Single centre
Kraemer (2018; USA)[54]	2014-2016	Mixed methods. Quantitative work had no control	Residents and fellows in medical specialities. Academic level NR	69 (41 residents, 28 fellows)	NR	Teaching sessions/ lectures. Faculty career development programme	Trainees	No	Single centre
Lin (2019; USA)[55]	Post 2009- NR	Qualitative	Post-training, post-doctoral, emergency medicine	17 (0:17)	12 Caucasian; 4 Asian American; 1 African American.	Mentorship programme - junior mentee/ senior mentor. Networking/ collaboration/ social activities, skill enhancement, e.g. negotiating skills	Junior Faculty, Managers/ leaders/ senior faculty	Gender	National
Moss (2008; Canada)[62]	2005-2005	Qualitative	Lecturers and assistant professors in Psychiatry	10 (6:4)	NR	Mentorship programme - peers	Junior Faculty	No	Single centre
Reader (2015; USA)[56]	2009-2009	Mixed methods. Quantitative work had no control	Post-training, speciality NR, mix of academic levels	10 (2:8)	NR	Mentorship programme - junior mentee/ senior mentor. Mentorship programme – peer. Teaching sessions/ lectures, protected research time. Faculty career development programme	Junior Faculty, Managers/ leaders/ senior faculty	Designed for "busy clinician educators"	Single centre

First author (Year of publication; country)	Years of data collection	Study design	Grade/type of clinical academics included	Number of participants (Male: Female)	Ethnicity	Intervention components	Target population of intervention	Aimed at particular group	Reach
Stubbe (2008; USA)[57]	NR- NR	Qualitative	Academic Child and Adolescent Psychiatry Residents. Academic level NR	5 (NR)	NR	Mentorship programme - junior mentee/ senior mentor, Teaching sessions/ lectures, Protected research time	Trainees	No	Single centre
Studies included within both quantitative and qualitative analysis									
Campion (2016; USA)[20]	2014-2014	Mixed methods. Quantitative work had concurrent control	Post-doctoral, clinical faculty and school of public health faculty	16 in intervention group (>60% women) 25 people in reference group	30% URM	Mentorship programme – peers. Teaching sessions/ lectures, protected research time, networking/ collaboration/ social activities	Junior faculty	No	Single centre
Guevara (2018; USA)[28]	NR- NR	Mixed methods. Quantitative work had concurrent control	Medical residents, junior staff and assistant profs. Academic level unclear	124 (65:59)	91 African American, 29 Hispanic Latino, 4 Native American	Funding award	Trainees, Junior Faculty	Ethnicity, URM	National

* Both papers by DeCastro et al.[50 51] and Klimas et al.[60 61] were based on data collected from a single sample of respondents.

Abbreviations: AFP – Academic Foundation Programme; CFSP – Clinical Faculty Scholars Program; CV – Curriculum Vitae; ELAM – Executive Leadership in Academic Medicine programme; EWIM – Early-career Women In Medicine program; GP – General Practitioner; MWIM – Mid-career Women In Medicine program; NR – Not reported; PRIME – Primary Medical Education program; RCT – Randomised control trial; UK – United Kingdom; URM – Underrepresented minority (study defined); USA – United States of America

For peer review only

For peer review only

Appendix 3: Quality assessment details

Randomised control trial

The single eligible randomised control trial had a low risk of bias for random sequence generation, blinding of outcome assessment, and incomplete outcome data items.[27] Participant blinding showed a high risk of bias as participants knew which groups they were assigned to. A risk of recruitment bias was also evident due to the cluster-randomised design. Allocation concealment and selective outcome reporting were unclear.[27]

Case-control studies

One of the two identified case-control study was rated medium to high quality for all items.[41] The other case-control study was of lower overall quality as it demonstrated a high risk of selection bias for intervention participant selection, and no methodological or analytical adjustments were made for comparing intervention and control groups.[26]

Cohort studies

The majority of the 25 included cohort studies had representative intervention populations,[19 21 22 24 25 29-35 37-40 43 44 46-48] appropriate control groups,[19 21-25 29 30 32-40 42 44-47] high quality outcome assessment methods,[19 21 24 29-31 34 36-38 40 45-47] and long follow-up periods.[19 21-25 29-32 34-36 38-40 42-44 46 47] However, comparability was mostly deemed poor as groups were often unmatched.[19 22 25 29 31 32 35 37-39 42 45-47] Only one study demonstrated high quality on all items,[24] whilst two studies scored positively on eight out of nine items, but failed to provide cohort follow-up information.[21 40] There were additional identified risks in group selection as several studies incorporated pre-selected or highly motivated populations for intervention groups (for example, Dannels et al.[23]).

Qualitative methodology studies

The majority of qualitative studies were of high quality for demonstrating congruity between the research methodology used and the: i) research question,[49-56 58-62 64] ii) data collection methods,[49-56 58-61 64 65] iii) data analysis and presentation,[49-56 58 60 61 64] and iv) interpretation of results.[49-56 58-62 64 65] However, inadequate reporting of important information was frequent with studies failing to provide statements relating to the relationship between the researchers and research itself.[49 52 54-61 64 65] Only one study demonstrated high quality on all items of the quality assessment tool.[53]

Realist evaluation

The realist evaluation study was rated medium to high quality on the majority of the criteria.[63] Only three aspects of the evaluation were deemed inadequate, which all related to incomplete methodological reporting, especially surrounding data analysis.[63]

Mixed methods studies

The two controlled mixed methods studies were deemed high quality on the majority of criteria.[20 28] Notably, Campion et al.,[20] demonstrated a lack of reporting for complete outcome data and failed to provide high quality methodological approaches for both the qualitative and quantitative components of the study. Guevara et al.,[28] failed to provide a rationale for using a mixed methods design. Finally, it was unclear whether the intervention was conducted as intended in both studies.[20 28]

For peer review only

For peer review only

Appendix 4: Table of outcome data for studies within quantitative analysis

First author (Year of publication)	Intervention group*	Control group*	Outcome	Sample size: intervention group	Sample size: control group	Estimate (95% CI/ p value/ SD)	Estimate adjusted for
**Aspiration**							
Dannels (2008)[23]	Programme participants	Two control groups: Applied but not accepted non-programme participants & general mid-career faculty women (also non-participants)	Aspiration to higher leadership position within an academic health centre (academic medicine)	55	27 & 178	76.4% vs 63.0% vs 49.4% (chi-squared= 12.903, p=0.002)	Unadjusted but faculty control group matched on academic rank, department chair status, race/ethnicity, discipline, degree type, basic sciences vs clinical department, age, medical school ownership, and awards ranking.
**Career Satisfaction**							
Campion (2016)[20]	Intervention participants	Reference group (non-participants)	Career satisfaction post-intervention	10	12	N=9 satisfied or very satisfied vs N=12 satisfied or very satisfied (NR)	Unadjusted
Grisso (2017)[27]	Intervention group	Control group (no intervention)	Work self-efficacy change scores	62	70	0.18 vs 0.24 (p=0.642)	Statistical tests were adjusted to account for correlation induced by the clustered design using generalized estimating equations. Both within-person factors (age, years in rank, race) and unit-level factors (intervention assignment) were modelled simultaneously
	Intervention group	Control group (no intervention)	Work-family conflict change (TWIF scores)	62	70	-0.13 vs -0.05 (p=0.541)	
	Intervention group	Control group (no intervention)	Work-family conflict change (SWIF scores)	62	70	-0.20 vs -0.23 (p=0.879)	
	Intervention group	Control group (no intervention)	CCWAS culture score change	Unclear; 109 in total	Unclear; 109 in total	0.03 vs 0.13 (p=0.274)	
Winn (2018)[44]	Academy participants	Pre-academy participants	Percentage of people who felt that the BCRP have been quite or extremely supportive in helping people to make career decisions	24	22	63% vs 45% (p=0.37)	Unadjusted
	Academy participants	Pre-academy participants	Percentage of people who agree or strongly	25	22	76% vs 63% (p=0.54)	

First author (Year of publication)	Intervention group*	Control group*	Outcome	Sample size: intervention group	Sample size: control group	Estimate (95% CI/ p value/ SD)	Estimate adjusted for
			agree that the BCRP guides and supports residents in pursuing and presenting scholarly projects				
**Skills & Knowledge**							
Campion (2016)[20]	Intervention participants	Reference group (non-participants)	Knowledge, Skills, and Attitudes Survey	9	11	No overall estimate. For 13 out of 32 items on the scale, participants showed greater gains than the reference group (NR)	Unadjusted
	Intervention participants	Reference group (non-participants)	Change in Sense of Community Index score (SCI-2 total score)	10	12	Mean change 10.6 vs 3.83 (p=0.2, SD=13.7 vs 9.40)	
Kohlwes (2006)[32]	Programme residents (PRIME)	Non-PRIME programme residents	Clinical competence score (average on a 9 point scale)	32	2294	8.23 vs 8.09 (p<0.001)	Unadjusted
Löwe (2008)[48]	Intervention residents	Control residents (from two different locations)	Mean change in total methodological research knowledge score from baseline	15	22	8.4 vs 1.2 (effect size =2.5, p<0.001, SD=2.9 vs 2.8)	Unadjusted
	Intervention residents	Control residents (from two different locations)	Mean change in total self-assessment research competence score from baseline	15	22	0.4 vs -0.4 (effect size=1.1, p=0.01, SD=0.9 vs 0.7)	

First author (Year of publication)	Intervention group*	Control group*	Outcome	Sample size: intervention group	Sample size: control group	Estimate (95% CI/ p value/ SD)	Estimate adjusted for
Winn (2018)[44]	Academy participants	Pre-academy participants	Percentage of people who agree or strongly agree that they have learned how to present a poster through BCRP activities	24	22	50% vs 29% (p=0.07)	Unadjusted
	Academy participants	Pre-academy participants	Percentage of people who agree or strongly agree that they have learned how to give a presentation/talk through BCRP activities	24	22	75% vs 36% (p=0.02)	
	Academy participants	Pre-academy participants	Percentage of people who agree or strongly agree with feeling prepared for non-clinical (scholarly) work after residency	24	22	63% vs 45% (p=0.37)	
**Funding**							
Goldenberg (2012)[26]	Programme trainees	Non-programme trainees	Annual grant dollars received (excluding fellowship award funding) at 3-4 years post-fellowship	11	9	Median $80,000 vs $23,000 (p=0.2)	Unadjusted
	Programme trainees	Non-programme trainees	Percentage of individuals who obtained K award within 5 years of beginning fellowship	9	8	33% vs 0% (p=0.21)	Unadjusted
Grisso (2017)[27]	Intervention group	Control group (no intervention)	Percentage of individuals with improved grants	62	70	Rate Ratio 0.75 (95% CI: 0.54-1.03, p=0.08)	Statistical tests were adjusted to account for correlation induced by the clustered design using generalized estimating equations.

For peer review only

First author (Year of publication)	Intervention group*	Control group*	Outcome	Sample size: intervention group	Sample size: control group	Estimate (95% CI/ p value/ SD)	Estimate adjusted for
							Both within-person factors (age, years in rank, race) and unit-level factors (intervention assignment) were modelled simultaneously
Guevara (2018)[28]	Interview applications scholars	Interview applications non-scholars	Total number of grants (mean)	76	48	2.0 vs 0.7 (p<0.001, SD=1.5 vs 1.0)	Adjusted for age, gender, race/ethnicity, application year, change of institutions, and rank at time of application
	Interview applications scholars	Interview applications non-scholars	Total grant dollars in thousands (mean)	76	48	$1,463 vs $567 (p=0.02, SD=$2,390 vs $1,507)	
Libby (2016)[34]	Programme participants (assistant professors)	Matched controls (non-programme participants, assistant professors)	Number of grant awards - change in mean before and after intervention	25	125	0.20 to 1.15 vs 0.30 to 0.49 (p<0.01)	Unadjusted but controls matched for key variables such as time in rank and dollars awarded
	Programme participants (assistant professors)	Matched controls (non-programme participants, assistant professors)	Amount of funding associated with grant awards - change in mean before and after intervention	25	125	$21,580 to $105,008 vs $26,742 to $53,716 (p<0.01)	
	Programme participants (assistant professors)	Matched controls (non-programme participants, assistant professors)	Percentage grant success rate (no. awards/no. proposals) - change before and after intervention	25	125	46.9% to 52.9% vs 42.5% to 51.7% (p=0.78)	
Löwe (2008)[48]	Intervention residents	Control residents (from two different locations)	Percentage of people who had grant applications accepted for funding	15	22	33% vs 0% (p=0.007)	Unadjusted
Sweeny (2019)[47]	After programme implementation participants	Before programme implementation participants	Number of grants awarded to new investigators	NR	NR	30 vs 20 (NR)	Unadjusted

For peer review only

First author (Year of publication)	Intervention group*	Control group*	Outcome	Sample size: intervention group	Sample size: control group	Estimate (95% CI/ p value/ SD)	Estimate adjusted for
**Research Participation**							
Brandt (2018)[19]	Research residents	Clinical residents	Percentage of participants actively engaged in research after intervention completion	43	284	42% vs 29% (p=0.04)	Unadjusted
Goldenberg (2012)[26]	Programme trainees	Non-programme trainees	Percentage of time spent in research post-fellowship	11	9	Median 80% vs 55% (p=0.01)	Unadjusted
Grisso (2017)[27]	Intervention group	Control group (no intervention)	Change in the number of total hours worked per week	62	70	-3.82 vs -1.39 (p=0.006)	Statistical tests were adjusted to account for correlation induced by the clustered design using generalized estimating equations. Both within-person factors (age, years in rank, race) and unit-level factors (intervention assignment) were modelled simultaneously
Harrison (2020)[29]	Participants after intervention implementation	Participants before intervention implementation	Total number of presentations (mean) - *just residents*	Unclear	Unclear	24.6 vs 3.0 (p=0.002, SD=10.24 vs 1.58)	Unadjusted
	Participants after intervention implementation	Participants before intervention implementation	Grant submissions (mean) - *just residents*	Unclear	Unclear	0.8 vs 0.0 (NR)	
	Participants after intervention implementation	Participants before intervention implementation	Total number of presentations (mean) - *just faculty*	Unclear	Unclear	40.0 vs 8.2 (p=0.025, SD=20.51 vs 1.30)	Unadjusted
Kohlwes (2016)[33]	Programme residents (PRIME)	Non-PRIME programme residents	Percentage of alumni who identified their primary role as a clinician investigator	71	98	35.2% vs 28.6% (chi-squared=0.843, p=0.358)	Unadjusted
	Programme residents (PRIME)	Non-PRIME programme residents	Mean percentage of professional time devoted	71	98	26.2% vs 20.6% (p=0.255)	

For peer review only

First author (Year of publication)	Intervention group*	Control group*	Outcome	Sample size: intervention group	Sample size: control group	Estimate (95% CI/ p value/ SD)	Estimate adjusted for
			to clinical or translational research				
Löwe (2008)[48]	Intervention residents	Control residents (from two different locations)	Percentage of people currently writing a journal article during 1 year program	15	22	86.7% vs 36.4% (p=0.003)	Unadjusted
	Intervention residents	Control residents (from two different locations)	Percentage of people currently writing a book article during 1 year program	15	22	6.7% vs 13.6% (p=0.63)	
	Intervention residents	Control residents (from two different locations)	Percentage of people who did presentations at scientific meetings during the last year	15	22	80% vs 40.9% (p=0.04)	
	Intervention residents	Control residents (from two different locations)	Percentage of people who submitted one or more grant proposal with first authorship in the last year	15	22	6.7% vs 0% (p=0.41)	
	Intervention residents	Control residents (from two different locations)	Percentage of people who submitted one or more grant proposal with co-authorship in the last year	15	22	46.7% vs 4.6% (p=0.004)	
**Mandel (2018)[35]	Graduates after residency programme transition	Graduates before residency programme transition	Percentage of graduates whose research activities were dedicated to clinical research during residency	19	9	84% vs 33% (p=0.013)	Unadjusted
	Graduates after residency programme transition	Graduates before residency programme transition	Percentage of graduates whose research activities were dedicated to full time basic	19	9	33% vs 22% (p=0.68)	

For peer review only

First author (Year of publication)	Intervention group*	Control group*	Outcome	Sample size: intervention group	Sample size: control group	Estimate (95% CI/ p value/ SD)	Estimate adjusted for
			science/translational research during residency				
	Graduates after residency programme transition	Graduates before residency programme transition	Percentage of graduates whose current practice included clinical research	19	9	79% vs 0% (p<0.001)	
	Graduates after residency programme transition	Graduates before residency programme transition	Percentage of graduates whose current practice included laboratory research	19	9	5% vs 0% (p=1)	
	Graduates after residency programme transition	Graduates before residency programme transition	Percentage of graduates whose current practice included academic practice	19	9	44% vs 0% (p=0.026)	
Sweeny (2019)[47]	After programme implementation participants	Before programme implementation participants	Number of research active emergency healthcare providers	NR	NR	181 vs 23 (NR)	Unadjusted
	After programme implementation participants	Before programme implementation participants	Number of presentations by emergency clinicians	NR	NR	61 vs 6 (NR)	
**Retention & Promotion**							
Chang (2016)[21]	Attendants from 1 of 3 career development programmes (all female)	Matched non-participants (all female)	Retention as assistant professor role (since first year of appointment)	732	4962	Log rank chi-squared 640.95, df=1 (p<0.001)	Adjusted for years in academic medicine, age, tenure track status, degree type, department type
	Attendants from 1 of 3 career development programmes (all female)	Matched non-participants (all female)	Likelihood of departure from academic medicine - *just assistant professors*	Unclear	Unclear	HR 0.85 (95% CI: 0.74-0.98)	

For peer review only - http://bmjopen.bmj.com/site/about/guidelines.xhtml

First author (Year of publication)	Intervention group*	Control group*	Outcome	Sample size: intervention group	Sample size: control group	Estimate (95% CI/ p value/ SD)	Estimate adjusted for
	Attendants from 1 of 3 career development programmes (all female)	Matched non-participants (all female)	Likelihood of departure from academic medicine - *just associate professors*	Unclear	Unclear	HR 0.76 (95% CI: 0.64-0.93)	
	Attendants from 1 of 3 career development programmes (all female)	Matched non-participants (all female)	Likelihood of departure from academic medicine - *just full professors*	Unclear	Unclear	HR 0.68 (95% CI: 0.50-0.92)	
	Attendants from 1 of 3 career development programmes (all female)	Matched non-participants (all female)	Departure from academic medicine after 10 years - *just assistant professors*	732	4962	20% vs 33% (NR)	
	Attendants from 1 of 3 career development programmes (all female)	Matched non-participants (all female)	Departure from academic medicine after 10 years - *just associate professors*	429	884	10% vs 17% (NR)	
	Attendants from 1 of 3 career development programmes (all female)	Matched non-participants (all female)	Departure from academic medicine after 10 years - *just full professors*	215	172	8% vs 17% (NR)	
	Attendants from 1 of 3 career development programmes (all female)	Matched non-participants (all female)	Long term retention in academic medicine - *just assistant professors*	732	4962	Intervention participants were less likely to leave academia in the periods 0-13 years after their appointment and	

For peer review only

First author (Year of publication)	Intervention group*	Control group*	Outcome	Sample size: intervention group	Sample size: control group	Estimate (95% CI/ p value/ SD)	Estimate adjusted for
						22 years after or longer (NR).	
	Attendants from 1 of 3 career development programmes (all female)	Matched non-participants (all female)	Long term retention in academic medicine - *just associate professors*	429	884	Intervention participants were less likely to leave academia in the periods 0-10 years after their appointment (NR)	
	Attendants from 1 of 3 career development programmes (all female)	Matched non-participants (all female)	Long term retention in academic medicine - *just full professors*	215	172	Intervention participants were less likely to leave academia in any period after their initial appointment (NR)	
Daley (2006)[22]	Junior faculty after implementation - intervention participants	Junior faculty before implementation - non-intervention participants	Percentage of URM junior staff retained at the faculty	15	12	87% vs 58% (z statistic=1.69, p=0.091)	Unadjusted
	Junior faculty after implementation - intervention participants	Junior faculty before implementation - non-intervention participants	Percentage of URM junior staff retained in academic medicine	15	12	93% vs 75% (z statistic=1.33, p=0.184)	
Ehlers (2018)[24]	Programme participants	Matched non-programme participants	Enter private practice post-programme	70	70	40% vs 38.6% (p=0.862)	Time since matriculation into fellowship was included as a covariate. Matching criteria included fellowship program (CV or GI), gender, years of post-MD graduate training (±1 year for 65 pairs and ±3 years for 5 pairs), age at the time of starting fellowship
	Programme participants	Matched non-programme participants	Enter academic practice post-programme	70	70	60% vs 61.4% (p=0.862)	

First author (Year of publication)	Intervention group*	Control group*	Outcome	Sample size: intervention group	Sample size: control group	Estimate (95% CI/ p value/ SD)	Estimate adjusted for
							training (±3 years), and site of fellowship (2 pairs needed to be matched across sites)
Emans (2008)[25]	Faculty 3 years after implementation	Faculty 3 years before implementation	Number of promotions - *male faculty only*	Unclear	Unclear	17% increase (NR)	Unadjusted
	Faculty 3 years after implementation	Faculty 3 years before implementation	Number of promotions - *female faculty only*	Unclear	Unclear	56% increase (NR)	Unadjusted
	Faculty 3 years after implementation	Faculty 3 years before implementation	Number of promotions - *URM faculty only*	Unclear	Unclear	60% increase (NR)	Unadjusted
	Faculty 3 years after implementation	Faculty 3 years before implementation	Number of promotions - *Asian faculty only*	Unclear	Unclear	60% increase (NR)	Unadjusted
Guevara (2018)[28]	Interview applications scholars	Interview applications non-scholars	Percentage of people who received any kind of position promotion	76	48	67.1% vs 58.3% (p=0.32)	Adjusted for age, gender, race/ethnicity, application year, change of institutions, and rank at time of application
	Interview applications scholars	Interview applications non-scholars	Retention in academic position (% of people)	76	48	84.2% vs 75% (p=0.21)	
Kohlwes (2006)[32]	Programme residents (PRIME)	Other program residents (UCSF internal medicine programs)	Percentage of people asked to be a chief resident	32	185	21.8% vs 9.2% (p=0.03)	Unadjusted
Kohlwes (2016)[33]	Programme residents (PRIME)	Non-PRIME programme residents	Percentage of alumni with an academic appointment	71	98	71.4% vs 67% (chi-squared=0.370, p=0.5430	Unadjusted
	Programme residents (PRIME)	Non-PRIME programme residents	Influence of research experience during residency on career choice (% strongly agree	71	98	63.4% vs 46.4% (chi-squared=4.757, p=0.029)	

First author (Year of publication)	Intervention group*	Control group*	Outcome	Sample size: intervention group	Sample size: control group	Estimate (95% CI/ p value/ SD)	Estimate adjusted for
			or agree on questionnaire)				
	Programme residents (PRIME)	Non-PRIME programme residents	Influence of success with research during residency on career choice (% strongly agree or agree on questionnaire)	71	98	36.6% vs 23.5% (chi-squared=3.458, p=0.063)	
Ockene (2017)[38]	Programme participants	Non-programme participants	Percentage of people promoted to associate professor	29	188	72% vs 32% (p<0.001)	Adjusted for equivalent time in rank
Ries (2009)[40]	NCLAM programme participants	Non-NCLAM programme participants hired during same period	Faculty retention after 8-year probationary period (univariate analysis)	120	719	HR=1.77 (95% CI: 1.20-2.61, p=0.004)	Unadjusted
	NCLAM programme participants	Non-NCLAM programme participants hired during same period	Faculty retention after 8-year probationary period (multivariate analysis)	120	719	HR = 1.67 (95% CI: 1.11-2.50, p=0.01)	Date of hire, gender and URM faculty were included as covariates
Ries (2012)[41]	NCLAM programme participants (1999-2006)	Matched non-NCLAM programme participants (1999-2006)	8-year probationary period retention rate (% of people retained)	113	202	67% vs 56% (p=0.04)	Matched for: (1) gender, (2) academic series (research versus clinical primary job description), (3) initial academic rank/step (academic experience when hired), (4) hire date (within 12 months, to control for temporal changes in internal and external institutional variables), and (5) department
**Recruitment**							
Brandt (2018)[19]	Research residents	Clinical residents	Percentage of people who entered first job in academic practice	43	284	34% vs 20% (p=0.0001)	Unadjusted
	Research residents	Clinical residents (before	Percentage of people	43	87	20% vs 14% (NR)	Unadjusted

For peer review only

First author (Year of publication)	Intervention group*	Control group*	Outcome	Sample size: intervention group	Sample size: control group	Estimate (95% CI/ p value/ SD)	Estimate adjusted for
	(after implementing programme)	implementing programme)	who currently or once held a full-time academic appointment				
Dannels (2008)[23]	Programme participants	Two control groups: Applied but not accepted non-programme participants & general mid-career faculty women (also non-participants)	Highest rank/ position professor	53	25 & 172	69.8% vs 48% vs 68.6% (NR)	Unadjusted but faculty control group matched on academic rank, department chair status, race/ethnicity, discipline, degree type, basic sciences vs clinical department, age, medical school ownership, and awards ranking.
	Programme participants	Two control groups: Applied but not accepted non-programme participants & general mid-career faculty women (also non-participants)	Highest rank/ position department chair/ director	52	27 & 160	25% vs 18.5% vs 15.6% (NR)	
	Programme participants	Two control groups: Applied but not accepted non-programme participants & general mid-career faculty women (also non-participants)	Highest rank/ position dean and above	52	27 & 160	11.5% vs 11.1% vs 4.4% (NR)	
	Programme participants	Two control groups: Applied but not accepted non-programme participants & general mid-career faculty women (also non-participants)	Academic title of chair or above	52	27 & 160	63.5% vs 37.0% vs 22.5% (chi-squared=29.96, p<0.001)	
Emans (2008)[25]	Faculty 5 years	Faculty year of	Percentage of professors	Unclear	Unclear	12% vs 14% (NR)	Unadjusted

For peer review only

First author (Year of publication)	Intervention group*	Control group*	Outcome	Sample size: intervention group	Sample size: control group	Estimate (95% CI/ p value/ SD)	Estimate adjusted for
	after programme implementation	programme implementation	- *female only*				
	Faculty 5 years after programme implementation	Faculty year of programme implementation	Percentage of associate professors - *female only*	Unclear	Unclear	30% vs 21% (p=0.023)	
	Faculty 5 years after programme implementation	Faculty year of programme implementation	Percentage of assistant professors - *female only*	Unclear	Unclear	44% vs 34% (NR)	
	Faculty 5 years after programme implementation	Faculty year of programme implementation	Percentage of instructors - *female only*	Unclear	Unclear	58% vs 53% (NR)	
	Faculty 5 years after programme implementation	Faculty year of programme implementation	Percentage of professors - *URM only*	Unclear	Unclear	3% vs 3% (NR)	
	Faculty 5 years after programme implementation	Faculty year of programme implementation	Percentage of associate professors - *URM only*	Unclear	Unclear	3% vs 2% (NR)	
	Faculty 5 years after programme implementation	Faculty year of programme implementation	Percentage of assistant professors - *URM only*	Unclear	Unclear	4% vs 5% (NR)	
	Faculty 5 years after programme implementation	Faculty year of programme implementation	Percentage of instructors - *URM only*	Unclear	Unclear	8% vs 8% (NR)	
	Faculty 5 years after programme implementation	Faculty year of programme implementation	Percentage of professors - *Asian only*	Unclear	Unclear	2% vs 0% (NR)	
	Faculty 5 years after programme implementation	Faculty year of programme implementation	Percentage of associate professors - *Asian only*	Unclear	Unclear	6% vs 3% (p=0.023)	
	Faculty 5 years after programme	Faculty year of programme	Percentage of assistant professors - *Asian only*	Unclear	Unclear	16% vs 9% (p=0.001)	

First author (Year of publication)	Intervention group*	Control group*	Outcome	Sample size: intervention group	Sample size: control group	Estimate (95% CI/ p value/ SD)	Estimate adjusted for
	implementation	implementation					
	Faculty 5 years after programme implementation	Faculty year of programme implementation	Percentage of instructors - *Asian only*	Unclear	Unclear	12% vs 12% (NR)	
Goldenberg (2012)[26]	Programme trainees (academic practice physicians only)	Non-programme trainees	Time from fellowship entry to assistant professor	11	9	Median 3.5 years vs 7 years (p<0.001)	Unadjusted
Joshua Smith (2014)[30]	Two intervention groups: Residents with protected research time and completing degree, & residents with just protected research time	Residents without protected research time	Percentage of residents for which their first job is an academic appointment	15 & 24	23	93.3% vs 58.3% vs 30.4% (p=0.046 comparing research time, p<0.001 comparing no research time)	Unadjusted
Khot (2011)[31]	NIH associates who entered academic medicine	Non-associates who entered academic medicine (identified from faculty roster database)	Academic rank achieved - full professor	1577	27821	Ratio 1.57 (95% CI: 1.49-1.66, p<0.001)	Unadjusted
	NIH associates who entered academic medicine	Non-associates who entered academic medicine (identified from faculty roster database)	Academic rank achieved - chair	1577	27821	Ratio 2.0 (95% CI: 1.78-2.24, p<0.001)	
	NIH associates who entered academic medicine	Non-associates who entered academic medicine (identified from faculty roster database)	Academic rank achieved - dean	1577	27821	Ratio 2.97 (95% CI: 2.21-3.98, p<0.001)	

First author (Year of publication)	Intervention group*	Control group*	Outcome	Sample size: intervention group	Sample size: control group	Estimate (95% CI/ p value/ SD)	Estimate adjusted for
	NIH associates who entered academic medicine	Non-associates who entered academic medicine (identified from faculty roster database)	Active faculty appointments in the top 10 schools	626	6038	Ratio 1.34 (95% CI: 1.15-1.56)	
	NIH associates who entered academic medicine	Non-associates who entered academic medicine (identified from faculty roster database)	Active faculty appointments in the top 20 schools	626	6038	Ratio 1.47 (95% CI: 1.33-1.63)	
Sheridan (2010)[42]	Intervention-participating departments	Non-intervention participating departments	Percentage of female faculty hired before and after workshop implementation	17	9	Precise estimate not reported - see Figure 1 in paper for details (p<0.05)	Unadjusted
Sweeny (2019)[47]	After programme implementation participants	Before programme implementation participants	Number of new principal investigators recruited	NR	NR	55 vs 17 (NR)	Unadjusted
Valantine (2014)[43]	University faculty where programme was implemented	Faculty at 6 peer universities	Change in female faculty as a percentage of total faculty; *including assistant, associate, and full professors* (2004-2010)	1219	17000	5.8% vs 4.5% (p=0.011)	Unadjusted
	University faculty where programme was implemented	Faculty at 6 peer universities	Change in female faculty as a percentage of total faculty; *just assistant professors* (2004-2010)	420	7362	4.7% vs 5.0% (p=0.573)	
	University faculty where programme was implemented	Faculty at 6 peer universities	Change in female faculty as a percentage of total faculty; *just associate professors* (2004-2010)	370	4546	5.8% vs 5.1% (p=0.385)	

For peer review only

First author (Year of publication)	Intervention group*	Control group*	Outcome	Sample size: intervention group	Sample size: control group	Estimate (95% CI/ p value/ SD)	Estimate adjusted for
	University faculty where programme was implemented	Faculty at 6 peer universities	Change in female faculty as a percentage of total faculty; *just full professors* (2004-2010)	429	5092	5.2% vs 4.0% (p=0.003)	
	University faculty where programme was implemented	National faculty cohort	Change in female faculty as a percentage of total faculty; *including assistant, associate, and full professors* (2004-2010)	1219	116996	5.8% vs 4.0% (p=0.001)	Unadjusted
	University faculty where programme was implemented	National faculty cohort	Change in female faculty as a percentage of total faculty; *just assistant professors* (2004-2010)	420	56509	4.7% vs 3.6% (p=0.340)	
	University faculty where programme was implemented	National faculty cohort	Change in female faculty as a percentage of total faculty; *just associate professors* (2004-2010)	370	27842	5.8% vs 4.1% (p=0.868)	
	University faculty where programme was implemented	National faculty cohort	Change in female faculty as a percentage of total faculty; *just full professors* (2004-2010)	429	31645	5.2% vs 3.7% (p=0.001)	
**Publications**							
Brandt (2018)[19]	Research residents	Clinical residents	Publication productivity over career	43	284	Median 14 vs 4 (p<0.0001)	Unadjusted
	Research residents	Clinical residents	Publication productivity during residency	43	284	Median 10 vs 2.5 (p<0.0001)	
Ehlers (2018)[24]	Programme participants	Matched non-programme participants	Number of publications at 5-years post-fellowship	70	70	Median 8 vs 5 (p=0.041)	Matching criteria included fellowship program (CV or GI), gender, years of post-MD graduate training (±1 year for 65
	Programme participants	Matched non-programme participants	Number of first-author publications at 5-years	70	70	Median 4 vs 2 (p=0.002)	

For peer review only

First author (Year of publication)	Intervention group*	Control group*	Outcome	Sample size: intervention group	Sample size: control group	Estimate (95% CI/ p value/ SD)	Estimate adjusted for
			post-fellowship				pairs and ±3 years for 5 pairs), age at the time of starting fellowship training (±3 years), and site of fellowship (2 pairs needed to be matched across sites)
	Programme participants	Matched non-programme participants	H-Index (as of year when studies was conducted - January 2018)	70	70	Median 11 vs 7 (p=0.013)	Time since matriculation into fellowship was included as a covariate. Matching criteria included fellowship program (CV or GI), gender, years of post-MD graduate training (±1 year for 65 pairs and ±3 years for 5 pairs), age at the time of starting fellowship training (±3 years), and site of fellowship (2 pairs needed to be matched across sites)
Goldenberg (2012)[26]	Programme trainees	Non-programme trainees	Annual number of peer-reviewed publications 3-4 years post-fellowship	11	9	Median 3.5 vs 1 (p=0.01)	Unadjusted
Grisso (2017)[27]	Intervention group	Control group (no intervention)	Increase in total no. of publications 2009 to 2012	62	70	Rate Ratio 0.80 (95% CI: 0.63-1.02, p=0.07)	Statistical tests were adjusted to account for correlation induced by clustered design using generalized estimating equations. Both within-person factors (age, years in rank, race) and unit-level factors (intervention assignment) were modelled simultaneously
	Intervention group	Control group (no intervention)	Increase in first author publications 2009 to 2012	62	70	Rate Ratio 1.00 (95% CI: 0.67-1.50, p=0.99)	
	Intervention group	Control group (no intervention)	Increase in total no. of peer review publications 2009 to 2012	62	70	Rate Ratio 0.95 (95% CI: 0.68-1.33, p=0.78)	
	Intervention group	Control group (no intervention)	Increase in first author peer review publications 2009 to 2012	62	70	Rate Ratio 1.06 (95% CI: 0.58-1.95, p=0.85)	

For peer review only

First author (Year of publication)	Intervention group*	Control group*	Outcome	Sample size: intervention group	Sample size: control group	Estimate (95% CI/ p value/ SD)	Estimate adjusted for
Guevara (2018)[28]	Interview applications scholars	Interview applications non-scholars	Total no. of publications (mean) from point of application to December 2013	76	48	27.2 vs 33.0 (p=0.47, SD=30.0 vs 58.8)	Adjusted for age, gender, race/ethnicity, application year, change of institutions, and rank at time of application
	Interview applications scholars	Interview applications non-scholars	H-Index (as of December 2013)	76	48	12.5 vs 10.9 (p=0.32, SD=7.9 vs 10.2)	
Harrison (2020)[29]	Participants after intervention implementation	Participants before intervention implementation	Mean number of publications (in 5 years) - *residents only*	Unclear	Unclear	7.0 vs 4.4 (p=0.263, SD= 4.47 vs 1.82)	Unadjusted
	Participants after intervention implementation	Participants before intervention programme	Mean number of publications (in 5 years) - *faculty only*	Unclear	Unclear	16.6 vs 12.8 (p=0.197, SD=4.39 vs 4.15)	Unadjusted
Joshua Smith (2014)[30]	Two intervention groups: Residents with protected research time and completing degree, & residents with just protected research time	Residents without protected research time	Number of publications (mean)	17 & 27	24	10.3 vs 5.30 vs 1.29 (p=0.001 comparing to research time, p<0.001 comparing to no research time)	Unadjusted
	Two intervention groups: Residents with protected research time and completing degree, & residents with just protected research time	Residents without protected research time	Number of first-author publications (mean)	17 & 27	24	4.06 vs 2.30 vs 0.46 (p=0.017 comparing to research time, p<0.001 comparing to no research time)	
	Two intervention groups: Residents with protected	Residents without protected research time	Impact factor of publications (mean)	17 & 27	24	32.3 vs 17.8 vs 2.69 (p=0.001 comparing to	

First author (Year of publication)	Intervention group*	Control group*	Outcome	Sample size: intervention group	Sample size: control group	Estimate (95% CI/ p value/ SD)	Estimate adjusted for
	research time and completing degree, & residents with just protected research time					research time, p<0.001 comparing to no research time)	
	Two intervention groups: Residents with protected research time and completing degree, & residents with just protected research time	Residents without protected research time	Adjusted impact factor of publications (mean)	17 & 27	24	65.4 vs 36.1 vs 5.17 (p=0.005 comparing to research time, p<0.001 comparing to no research time)	Adjusted for level of authorship
Klimas (2017)[45]	Programme participants	Non-programme participants	Total number of published papers at the end of the one-year fellowship	4	4	7 vs 1 (p=0.1)	Unadjusted
Kohlwes (2016)[33]	Programme residents	Non-programme residents	Percentage of alumni who published research they had started during residency (first-author peer-review article)	71	92	64.3% vs 40.2% (chi-squared=9.213, p=0.002)	Unadjusted
Löwe (2008)[48]	Intervention residents	Control residents (from two different locations)	Percentage of people who completed one or more original publications with first authorship in the last year	15	22	46.7% vs 22.7% (p=0.13)	Unadjusted
	Intervention residents	Control residents (from two different locations)	Percentage of people who completed one or more original publications with co-authorship in the last year	15	22	60% vs 18.2% (p=0.01)	

For peer review only

First author (Year of publication)	Intervention group*	Control group*	Outcome	Sample size: intervention group	Sample size: control group	Estimate (95% CI/ p value/ SD)	Estimate adjusted for
	Intervention residents	Control residents (from two different locations)	Percentage of people who completed one or more review or meta-analysis with first authorship in the last year	15	22	0% vs 9.1% (p=0.5)	
	Intervention residents	Control residents (from two different locations)	Percentage of people who completed one or more review or meta-analysis with co-authorship in the last year	15	22	0% vs 9.1% (p=0.5)	
	Intervention residents	Control residents (from two different locations)	Percentage of people who completed one or more book article with first authorship in the last year	15	22	26.7% vs 18.2% (p=0.69)	
	Intervention residents	Control residents (from two different locations)	Percentage of people who completed one or more book article with co-authorship in the last year	15	22	20% vs 4.6% (p=0.28)	
Merani (2014)[46]	Additional degree trainees: Masters trainees, & PhD trainees	Clinical-only trainees	Percentage of surgeons involved in research publication during training with any authorship role	72 & 33	218	81.9% vs 100% vs 38.1% (p<0.05)	Unadjusted
	Additional degree trainees: Masters trainees, & PhD trainees	Clinical-only trainees	Percentage of surgeons involved in research publication during training with principal author role	72 & 33	218	58.3% vs 100% vs 27.1% (p<0.05)	
	Additional degree trainees: Masters	Clinical-only trainees	Percentage of surgeons involved in research	72 & 33	218	19.4% vs 12.1% vs 5% (p<0.05)	

First author (Year of publication)	Intervention group*	Control group*	Outcome	Sample size: intervention group	Sample size: control group	Estimate (95% CI/ p value/ SD)	Estimate adjusted for
	trainees, & PhD trainees		publication during training with senior author role				
	Additional degree trainees: Masters trainees, & PhD trainees	Clinical-only trainees	Percentage of surgeons involved in research publication post-training with any authorship role	69 & 32	201	81.2% vs 90.6% vs 44.3% (p<0.05)	
	Additional degree trainees: Masters trainees, & PhD trainees	Clinical-only trainees	Percentage of surgeons involved in research publication post-training with principal author role	69 & 32	201	56.5% vs 71.9% vs 26.4% (p<0.05)	
	Additional degree trainees: Masters trainees, & PhD trainees	Clinical-only trainees	Percentage of surgeons involved in research publications post-training with senior author role	69 & 32	201	37.7% vs 46.9% vs 14.9% (p<0.05)	
Mills (2011)[36]	Programme participants	Non-programme participants	Percentage of participants with publication after graduation	232	295	69% vs 34% (p<0.001)	Unadjusted
	Programme participants	Non-programme participants	Median number of publications after graduation	232	295	2 vs 0 (p<0.001)	
	Programme participants	Non-programme participants	Likelihood of publishing in the future	232	295	OR=3.7 (95% CI: 2.5-5.6)	Adjusted for gender and publications prior to programme
Nasab (2019)[37]	Programme participants	Non-programme participants (before implementation of programme)	Number of published papers during course period (2015-2018) - *just junior faculty members*	14	26	Mean 9.21 vs 4 (p=0.003, SD=8.1 vs. 5.21)	Unadjusted
	Programme participants	Non-programme participants (before implementation of	Number of published papers during course period (2015-2018) - *just*	35	11	Mean 4.31 vs 2 (p=0.008, SD=3.44 vs. 1.67)	

First author (Year of publication)	Intervention group*	Control group*	Outcome	Sample size: intervention group	Sample size: control group	Estimate (95% CI/ p value/ SD)	Estimate adjusted for
		programme)	*fellows*				
	Programme participants	Non-programme participants (before implementation of programme)	Journal impact factor during course period (2015-2018) - *just junior faculty members*	14	26	Mean 3.09 vs 4.57 (p=0.67, SD=1.54 vs. 5.03)	
	Programme participants	Non-programme participants (before implementation of programme)	Journal impact factor during course period (2015-2018) - *just fellows*	35	11	Mean 5.46 vs 3.03 (p=0.776, SD=9.81 vs. 1.72)	
Patel (2018)[39]	Programme cohorts (2011/2012 - 2014/2015)	Pre-programme cohorts (2007/2008 - 2010/2011)	Likelihood of publishing one or more clinical research articles (%)	35	36	77% vs 44% (chi-squared=7.9, p=0.005)	Unadjusted
	Programme cohorts (2011/2012 - 2014/2015)	Pre-programme cohorts (2007/2008 - 2010/2011)	Number of published clinical research articles during residency (mean)	35	36	2.09 vs 1.56 (NR)	

*Groups are reported as described by authors. **Inconsistent data reported and referred to in tables and text; data represented here is extracted from text.

Abbreviations: BCRP - Boston Combined Residency Programme; CCWAS - Culture that is conducive to women's academic success; NCLAM - National Center of Leadership in Academic Medicine; NR - Not Reported; PRIME - Primary Medical Education programme; SWIF - Strain-based work interference with family; TWIF - Time-based work interference with family; UCSF - University of California San Francisco; URM - Underrepresented Minorities.

For peer review only

PRISMA 2020 Checklist

Section and Topic	Item #	Checklist item	Location where item is reported
**TITLE**			
Title	1	Identify the report as a systematic review.	Page 1
**ABSTRACT**			
Abstract	2	See the PRISMA 2020 for Abstracts checklist.	Page 2
**INTRODUCTION**			
Rationale	3	Describe the rationale for the review in the context of existing knowledge.	Page 3
Objectives	4	Provide an explicit statement of the objective(s) or question(s) the review addresses.	Page 3
**METHODS**			
Eligibility criteria	5	Specify the inclusion and exclusion criteria for the review and how studies were grouped for the syntheses.	Page 3-4
Information sources	6	Specify all databases, registers, websites, organisations, reference lists and other sources searched or consulted to identify studies. Specify the date when each source was last searched or consulted.	Page 3
Search strategy	7	Present the full search strategies for all databases, registers and websites, including any filters and limits used.	Page 3 (and reported in full in published protocol)
Selection process	8	Specify the methods used to decide whether a study met the inclusion criteria of the review, including how many reviewers screened each record and each report retrieved, whether they worked independently, and if applicable, details of automation tools used in the process.	Page 4
Data collection process	9	Specify the methods used to collect data from reports, including how many reviewers collected data from each report, whether they worked independently, any processes for obtaining or confirming data from study investigators, and if applicable, details of automation tools used in the process.	Page 4
Data items	10a	List and define all outcomes for which data were sought. Specify whether all results that were compatible with each outcome domain in each study were sought (e.g. for all measures, time points, analyses), and if not, the methods used to decide which results to collect.	Page 4 (and reported in full in published protocol)
	10b	List and define all other variables for which data were sought (e.g. participant and intervention characteristics, funding sources). Describe any assumptions made about any missing or unclear information.	Page 4 (and reported in full in published protocol)
Study risk of bias assessment	11	Specify the methods used to assess risk of bias in the included studies, including details of the tool(s) used, how many reviewers assessed each study and whether they worked independently, and if applicable, details of automation tools used in the process.	Page 4
Effect measures	12	Specify for each outcome the effect measure(s) (e.g. risk ratio, mean difference) used in the synthesis or presentation of results.	Page 4, and Appendix 2
Synthesis methods	13a	Describe the processes used to decide which studies were eligible for each synthesis (e.g. tabulating the study intervention characteristics and comparing against the planned groups for each synthesis (item #5)).	Page 4
	13b	Describe any methods required to prepare the data for presentation or synthesis, such as handling of missing summary statistics, or data conversions.	Page 4

For peer review only

PRISMA 2020 Checklist

Section and Topic	Item #	Checklist item	Location where item is reported
	13c	Describe any methods used to tabulate or visually display results of individual studies and syntheses.	Page 4
	13d	Describe any methods used to synthesize results and provide a rationale for the choice(s). If meta-analysis was performed, describe the model(s), method(s) to identify the presence and extent of statistical heterogeneity, and software package(s) used.	Page 4
	13e	Describe any methods used to explore possible causes of heterogeneity among study results (e.g. subgroup analysis, meta-regression).	Page 4 (and reported in full in published protocol)
	13f	Describe any sensitivity analyses conducted to assess robustness of the synthesized results.	Page 4 (and reported in full in published protocol)
Reporting bias assessment	14	Describe any methods used to assess risk of bias due to missing results in a synthesis (arising from reporting biases).	Page 4
Certainty assessment	15	Describe any methods used to assess certainty (or confidence) in the body of evidence for an outcome.	N/A
**RESULTS**			
Study selection	16a	Describe the results of the search and selection process, from the number of records identified in the search to the number of studies included in the review, ideally using a flow diagram.	Page 4 and Figure 1
	16b	Cite studies that might appear to meet the inclusion criteria, but which were excluded, and explain why they were excluded.	N/A
Study characteristics	17	Cite each included study and present its characteristics.	Page 4-5 and Table 1
Risk of bias in studies	18	Present assessments of risk of bias for each included study.	Page 5 and appendix 1
Results of individual studies	19	For all outcomes, present, for each study: (a) summary statistics for each group (where appropriate) and (b) an effect estimate and its precision (e.g. confidence/credible interval), ideally using structured tables or plots.	Appendix 2
Results of syntheses	20a	For each synthesis, briefly summarise the characteristics and risk of bias among contributing studies.	Page 5-9
	20b	Present results of all statistical syntheses conducted. If meta-analysis was done, present for each the summary estimate and its precision (e.g. confidence/credible interval) and measures of statistical heterogeneity. If comparing groups, describe the direction of the effect.	N/A
	20c	Present results of all investigations of possible causes of heterogeneity among study results.	N/A
	20d	Present results of all sensitivity analyses conducted to assess the robustness of the synthesized results.	N/A
Reporting biases	21	Present assessments of risk of bias due to missing results (arising from reporting biases) for each synthesis assessed.	N/A
Certainty of evidence	22	Present assessments of certainty (or confidence) in the body of evidence for each outcome assessed.	N/A
**DISCUSSION**			
Discussion	23a	Provide a general interpretation of the results in the context of other evidence.	Page 10-11

For peer review only
For peer review only - http://bmjopen.bmj.com/site/about/guidelines.xhtml

PRISMA 2020 Checklist

Section and Topic	Item #	Checklist item	Location where item is reported
	23b	Discuss any limitations of the evidence included in the review.	Page 10-11
	23c	Discuss any limitations of the review processes used.	Page 10-11
	23d	Discuss implications of the results for practice, policy, and future research.	Page 10-11
**OTHER INFORMATION**			
Registration and protocol	24a	Provide registration information for the review, including register name and registration number, or state that the review was not registered.	Page 2 and 3
	24b	Indicate where the review protocol can be accessed, or state that a protocol was not prepared.	Page 3
	24c	Describe and explain any amendments to information provided at registration or in the protocol.	Page 3-4
Support	25	Describe sources of financial or non-financial support for the review, and the role of the funders or sponsors in the review.	Page 12
Competing interests	26	Declare any competing interests of review authors.	Page 12
Availability of data, code and other materials	27	Report which of the following are publicly available and where they can be found: template data collection forms; data extracted from included studies; data used for all analyses; analytic code; any other materials used in the review.	Page 12

From: Page MJ, McKenzie JE, Bossuyt PM, Boutron I, Hoffmann TC, Mulrow CD, et al. The PRISMA 2020 statement: an updated guideline for reporting systematic reviews. BMJ 2021;372:n71. doi: 10.1136/bmj.n71

For more information, visit: http://www.prisma-statement.org/

For peer review only
